# Smoking trends and health equity in Switzerland between 1992 and 2017: dependence of smoking prevalence on educational level and social determinants

**DOI:** 10.3389/fpsyt.2023.1258272

**Published:** 2023-11-23

**Authors:** Daniel Wehrli, Hans Gilljam, Dow Mu Koh, Simon Matoori, Thomas Sartoretti, Stefan Boes, Martin Hartmann, Katharina Roser, Alexander Ort, Philippe Wanner, Dorothee Harder, Rasmus Bech-Hohenberger, Johannes M. Froehlich, Georg Marcus Fröhlich, Jochen Mutschler, Tino Plümecke, Andreas Gutzeit

**Affiliations:** ^1^Faculty of Medicine, University of Zurich, Zurich, Switzerland; ^2^Faculty of Health Sciences and Medicine, University of Lucerne, Lucerne, Switzerland; ^3^Department of Global Public Health, Karolinska Institutet, Stockholm, Sweden; ^4^Cancer Research UK Clinical Magnetic Resonance Research Group, Institute of Cancer Research, Sutton, Surrey, United Kingdom; ^5^Faculté de Pharmacie, Université de Montréal, Montreal, QC, Canada; ^6^Institute of Philosophy, University of Lucerne, Lucerne, Switzerland; ^7^Institute of Demography and Socioeconomics, University of Geneva, Geneva, Switzerland; ^8^Department of Radiology, Clinic of Radiology and Nuclear Medicine, University Hospital Basel, Basel, Switzerland; ^9^Heart Clinic Lucerne, Lucerne, Switzerland; ^10^Psychiatric Services Lucerne, Lucerne, Switzerland; ^11^Institute of Sociology, University of Freiburg, Freiburg im Breisgau, Germany; ^12^Department of Radiology, Paracelsus Medical University, Salzburg, Austria; ^13^Department of Chemistry and Applied Biosciences, Institute of Pharmaceutical Sciences, ETH Zurich, Zurich, Switzerland; ^14^Department of Radiology and Nuclear Medicine, Cantonal Hospital Schaffhausen, Schaffhausen, Switzerland; ^15^Department of Health Sciences and Medicine, Universität Luzern, Lucerne, Switzerland

**Keywords:** health equity, nicotine consumption, education level, addiction, health policy

## Abstract

**Background:**

Switzerland ranks among the top three healthcare systems in the world with regards to healthcare access, suggesting a high degree of health equity. However, Switzerland has few preventive strategies against smoking abuse. The aim of this study is to clarify whether educational level and citizenship status have an influence on the prevalence of smoking in Switzerland and whether there is health inequity related to a lack of preventive strategies.

**Methods:**

We based our analysis on publicly available health data published in the Swiss government's Swiss health survey (1992–2017). We compared the prevalence of smoking across the years and correlated these data with levels of educational attainment, citizenship status and age.

**Results:**

A continuous significant decline in smokers is observed in the highest education group (TERT). Over time, prevalence was reduced from 29% in 1992 to 23% in 2017 (*p* < 0.001). The intermediate-level educational group (SEK 2) showed smaller but also significant decline on a 0.05 sigificance level over the same period, from 31% to 29% (*p* = 0.003). The lowest educational group showed a nonsignificant decline from 28% to 27% (*p* = 0.6). The population who holds Swiss citizenship showed a decrease in smoking from 28% to 26% within the time frame (*p* < 0.001). People without Swiss citizenship had a much higher prevalence of smokers, at 38% in 1992 and declining to 32% in 2017 (*p* < 0.001). All cohorts from age 15 to age 64 have a far higher prevalence of smokers than cohorts at an older age, with the highest prevalence in the 25–34 age group.

**Conclusion:**

In Switzerland, individuals with lower levels of education and non-Swiss populations are more susceptible to health risk of smoking. This is despite the existence of a high-quality healthcare system that has nevertheless failed to negated health inequities.

## 1 Introduction

Smoking is the greatest avoidable health risk in Switzerland. It is estimated that around 10,000 people die every year from smoking-related diseases. This corresponds to around 27 deaths per day, representing almost 15% of all deaths and leading to costs of around 5 billion Swiss francs per year (1, 2). Fortunately, as several reports in recent years have shown, there has been a shift in the Swiss population, with a reduction in the prevalence of smoking (3, 4).

For decades, there has been a global debate on how to protect people from harms of smoking. Policy interventions, such as limiting access to tobacco and implementing educational programs, are frequently proposed as preventive instruments (5, 6). Although 50 of 53 countries in the WHO European Region are parties to the WHO Framework Convention for Tobacco Control (FCTC), smoking prevalence varies tremendously between European countries (7). Switzerland is one of the few countries that has so far refused to implement the FCTC (8). Despite the lack of FCTC ratification, the Global Tobacco Industry Interference Index was derived in Switzerland. The score describes the lack of restriction for the tobacco lobby (9). With 92 out of a possible 100 points, Switzerland is in 79th place in 2021 (out of 80 states). Among the 16 European states surveyed, Switzerland scored the worst and thus may be considered very tobacco industry-friendly (10).

According to the WHO, health equity is the absence of health disadvantages depending on social factors such as age, education, nationality or socioeconomic status. Conversely, social inequalities in the health system are referred to as health inequity (11). The prevalence of smoking is unfortunately not a universally accepted marker of health equity. In addition, the determination of prevalence of nicotine use across the whole population does not reveal any difference in prevalence in subpopulations. According to previous research, individuals who continue to smoke are more likely to have limited formal education and lack access to educational opportunities, and they tend to belong to marginalized or underprivileged groups of the population (12–15). Therefore, the prevalence of smoking serves as a marker of health equity across different subpopulations.

Switzerland ranks among the top three healthcare systems in the world with regards to access to healthcare and successful treatment of diseases, indicating a high degree of equity (16). The high standard of healthcare in Switzerland is also reflected in life expectancy. With a mean life expectancy of 82.8 years, this is one of the highest in Europe and well above the EU average (17).

The high standard of healthcare on the one hand and the comparatively high prevalence of smoking on the other result in a contradictory situation: while Switzerland has made remarkable achievements in social and health care, independently of individual socioeconomic background, it also has one of the lowest standards in Europe for smoking prevention (9, 10).

The purpose of this study is to analyze whether the decreasing trend in smoking prevalence observed in recent years in Switzerland is supported by the latest data. Furthermore, we aim to clarify whether the relationship between socioeconomic status, as indicated by educational level and immigration status, and the prevalence of nicotine consumption is a valid one. These questions are of particular relevance in Switzerland, a country with one of the lowest smoking prevention standards in Europe.

## 2 Methods

### 2.1 Swiss health survey

This study is a descriptive analysis of publicly available anonymous statistical data from the Federal Statistical Office in Switzerland (BFS) and does not require an ethics application. Our analysis is based on the data published by the Swiss government's health survey over the past three decades. This survey is part of the federal government's multiyear statistical program and has been conducted every five years since 1992 (1992, 1997, 2002, 2007, 2012, 2017). The represented population includes all persons aged 15 and over who live in private households, including people without Swiss citizenship. The net sample includes 10,000 people from the Swiss population and changes each time the survey is conducted. In addition, the individual cantons had the option of increasing the sample size in their canton, in order to be able to carry out representative evaluations at cantonal level. Eighteen cantons and the city of Zurich availed themselves of this opportunity in 2017 and financed additional interviews for their areas. The net sample of the Swiss Health Survey 2017 thus includes 22,134 telephone interviews. Following the telephone survey, a written questionnaire was also sent to the participants, which was returned by 18,832 people. The questionnaire is attached here as an Appendix 1. The objective of the survey is to measure the health status of the population, determine the development over time, and observe the impact of health policy measures. In this survey, an inhaled tobacco product is interpreted as smoking. Among the smokers, most smoke every day. A smaller number are occasional smokers, but this group is heterogeneous and not clearly defined. To improve clarity, daily smokers and occasional smokers are combined into one group of smokers from Swiss Federal Statistical Office.

Substitutes, such as oral or transcutaneous nicotine administration, are listed under a separate heading, but were practically nonexistent in Switzerland during this period. Newer forms of inhaled products such as electronic cigarettes are also counted as smoking. These products were also scarcely available during this period. In Switzerland there are no official figures on sales statistics for tobacco products.

### 2.2 Education level

For comparison with smoking prevalence, the BFS classified the education level attained as obligatory school (OS), secondary education (SEK 2), and tertiary level (TERT). Obligatory schooling (OS) includes two years of kindergarten and nine years of school. Secondary education (SEK 2) includes further education with practical training. Tertiary levels of education (TERT)include universities, universities of applied sciences, and higher vocational examinations. The educational profile of the Swiss population in 2017 may be broken down as follows: 34.7% with a tertiary qualification (TERT), 45.0% with a secondary qualification (SEK 2), and 20.3% with a maximum compulsory (OS) school qualification (https://www.bfs.admin.ch/asset/de/23965915, accessed: July 6, 2023).

### 2.3 Citizenship

Switzerland has the highest proportion of people with an immigrant status in Europe. More than 39% of the permanent resident population 15 years and older has a migrant background, and about 25% of permanent residents do not have a Swiss passport (18). We correlated smoking prevalence between people with and without Swiss citizenship. To the best of our knowledge, this has never been analyzed or published in detail.

### 2.4 Statistical analysis

The analysis is based on the original data file of the BFS “su-d-14.02-ESS-TABAC3_CH.xlsx” The data were evaluated with the software R (Version 4.2.1) by a statistician (kaufmann@biostatistics.ch). The plots (produced with the package “ggplot2”) show the trends in smoking prevalence stratified by educational level, citizenship, and age, respectively, for the years 1992, 1997, 2002, 2007, 2012, and 2017 stratified by education, citizenship, and age, respectively. The error bars with the colored shadings reflect the 95% confidence intervals [+/- 95%-CI]. *P*-values as a quantitative measure to confirm the qualitative observations have been calculated using Chi-squared test (confidence leve l.05) by comparing the frequencies of smokers and nonsmokers between 1992 and 2017 for education and citizenship, as well as by comparing the frequencies of smokers with and without Swiss citizenship for each year. Smokers include daily smokers and occasional smokers.

## 3 Results

### 3.1 Smoking behavior in correlation to level of education

**Figure 1:**
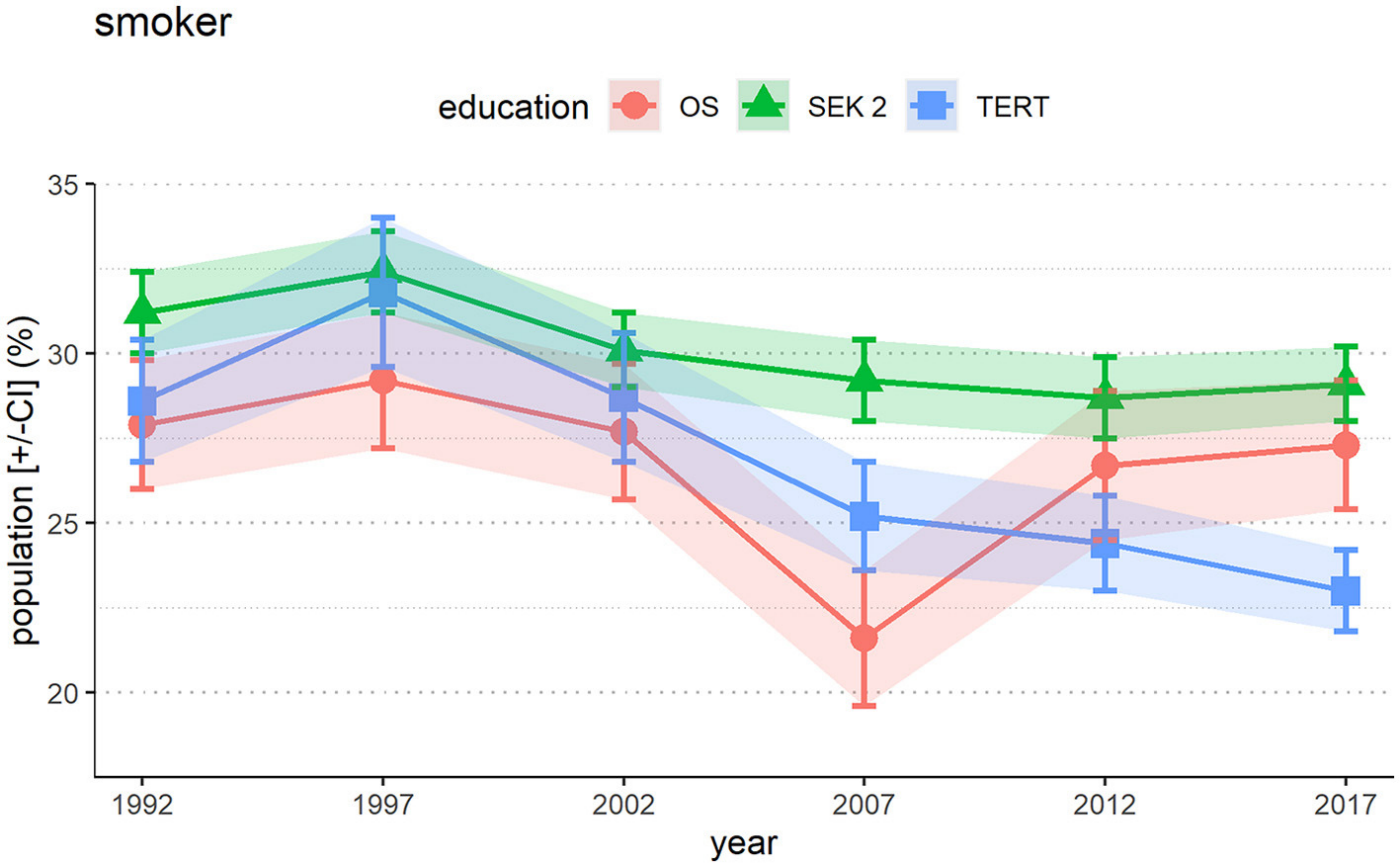
Prevalence of smokers in Switzerland depending on education level. The data show a strongly significant drop in the group with the highest level of education (TERT), while the drop in the group with SEK 2 is less pronounced but significant, and for obligatory school (OS) the frequency stays at a high level. Daily smokers and occasional smokers are summarized into one group of smokers. The error bars [+/-CI] with the colored shadings reflect the 95% confidence intervals.

Figure 1 summarizes smoking behavior in Switzerland since 1992. Values fluctuate, but there is a continuous decline evident in smokers in the highest education qualifications group (TERT). There is statistically significant drop in smoking prevalence from 28.6% in 1992 to 23.0% in 2017 (*p* < 0.001). The middle education group (SEK 2) shows a slight but significant decline over the years, with a prevalence between 31.2 and 29.1% (*p* = 0.003). At the lowest level of educational qualifications (OS), the prevalence does not significantly change, with values between 27.9 and 27.3% (*p* = 0.6). The outlying low value of smoking prevalence in the year 2007, with 21.6%, is hard to explain. According to statements by the Federal Statistical Office, the educational profile of the Swiss population is summarized in the Material and Methods section.

### 3.2 Smoking prevalence in Switzerland according to citizenship

**Figure 2:**
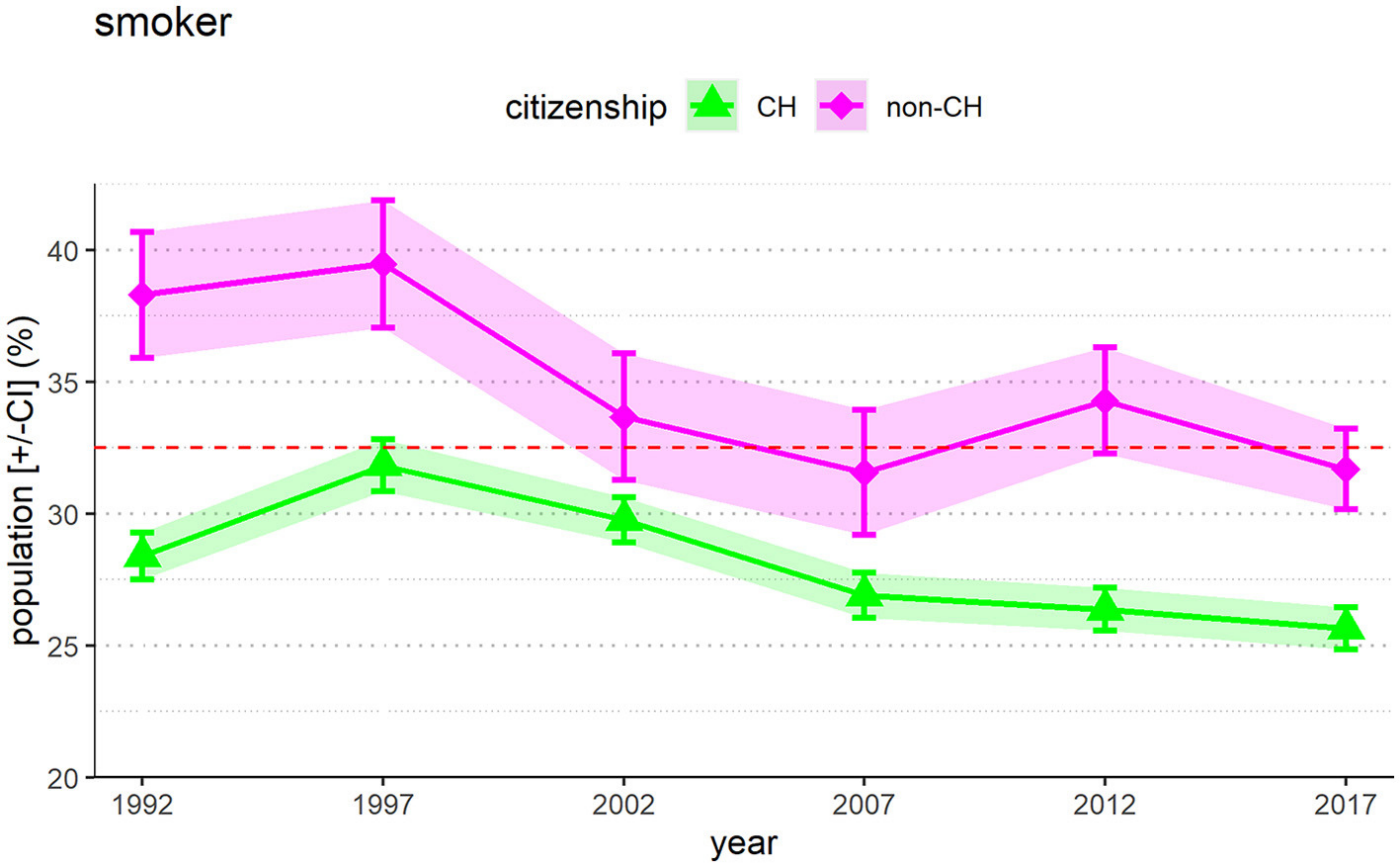
People in Switzerland without Swiss citizenship show a higher prevalence of smokers than people with a Swiss citizenship. It should be kept in mind that the group of people without a Swiss passport makes up only 25% of the population while 75% hold a Swiss passport. The error bars [+/-CI] with the colored shadings reflect the 95% confidence intervals.

Figure 2 summarizes smoking prevalence in Switzerland according to citizenship. People without a Swiss passport (non-CH) smoke statistically significantly more frequently than people with a Swiss passport (CH) (*p* < 0.001 for every year). For both categories, there is a strong statistically significant tendency for smokers to decrease over time. Swiss citizens show a decrease of the overall prevalence of current smokers of 2.7% (from 28.4% in 1992 to 25.7% in 2017, *p* < 0.001). Non-Swiss show a more marked decrease of 6.6% (from 38.3% in 1992 to 31.7% in 2017, *p* < 0.001), but the rate of smokers is still massively higher than among people with Swiss citizenship.

### 3.3 Smoking rate by age

**Figure 3:**
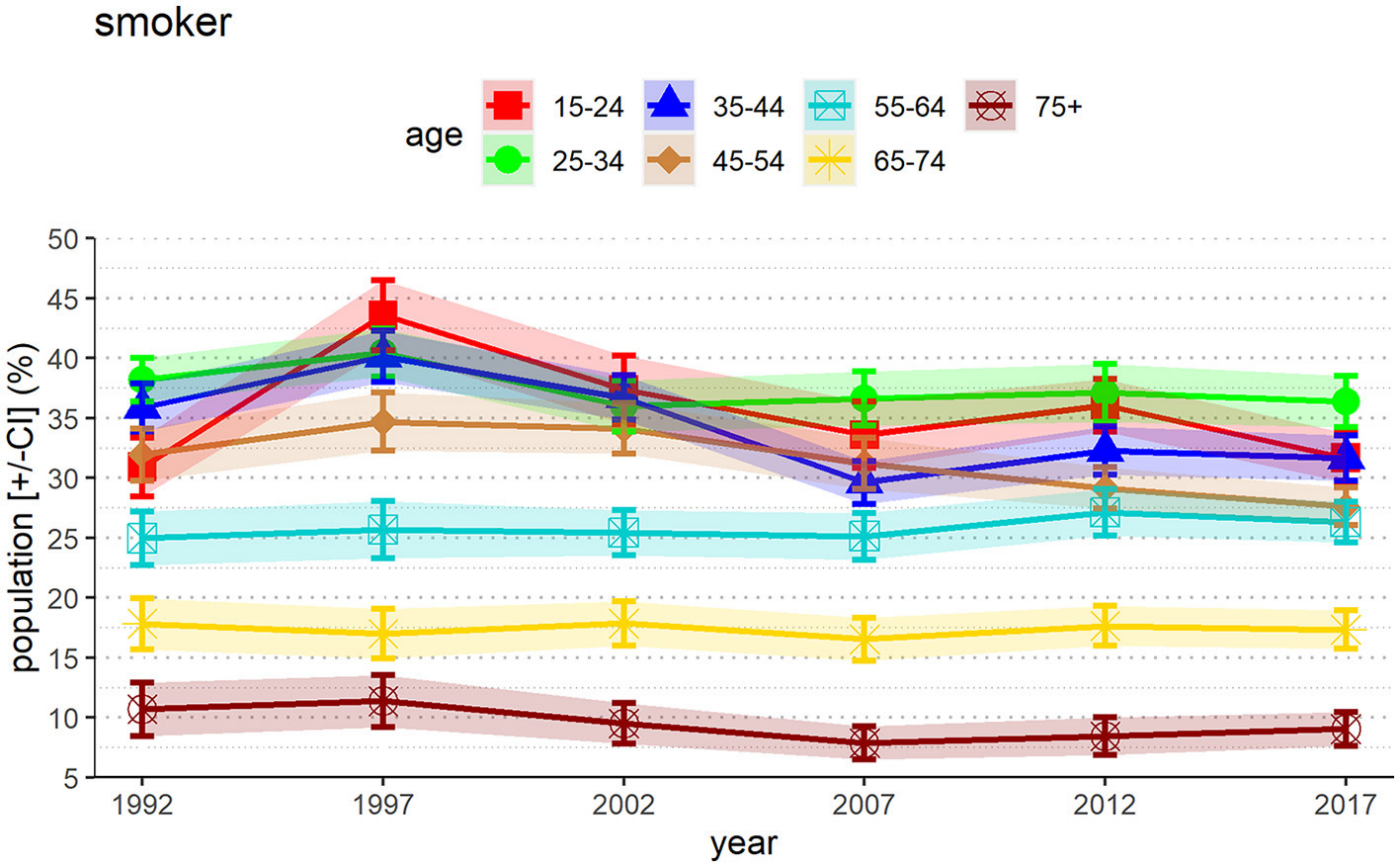
Percentage of smokers in Switzerland according to age. There is a high proportion of smokers among very young adults. The error bars [+/-CI] with the colored shadings reflect the 95% confidence intervals.

Figure 3 shows that there is a relationship between smoking and age and that the proportion of smokers among young people in Switzerland is particularly high. All cohorts from age 15 to age 64 have a far higher prevalence of smokers than cohorts at an older age, with the highest prevalence in the 25–34 age group. Between 25 and 38% of the population in these age groups are smokers.

The percentage of smokers decreases with increasing age. The proportion of 75-year-olds and older who smoke has been below 10% since at least 2007. However, the low number in this age group may also reflect the fact that smokers in this group meet an earlier death than nonsmokers. The very high proportion of smokers in the younger age group is a matter of concern from an epidemiological viewpoint.

## 4 Discussion

Existing research has identified demographic groups who are at greater risk for persistent tobacco use. These include individuals who are socioeconomically disadvantaged, have lower levels of education, experience poorer mental health, identify as sexual and/or gender minorities or belong to racial and/or ethnic minorities (19–23). In the sociomedical context, these can be interpreted as expressions of health inequity. Health inequity is better recognized in countries where disadvantaged social groups are clearly identified and present as a challenge (24, 25).

Switzerland is considered one of the wealthiest nations globally, with a highly regarded healthcare system and one of the highest life expectancies in the world (16, 17). These socioeconomic indicators can be taken as indications of a relatively high level of health equity and equal opportunities. The country has a well-developed and specialized healthcare system accessible to almost all parts of the population, including those from a low socioeconomic background, with some exceptions for certain vulnerable groups such as so-called sans papiers, which were also not recorded in this study (16, 24, 25). Paradoxically, Switzerland scores among the lowest countries regarding regulations restricting tobacco sales and consumption, and hardly any other country in Europe is as heavily influenced by the tobacco lobby as Switzerland (8–10). If the inverse relation between health equity and smoking consumption is correct, Switzerland should see a decrease in smoking in educated people with higher socioeconomic status.

In our study, we showed that people at with highest education level in Switzerland had a significant decrease in smoking prevalence over the last years (*p* < 0.05). Specifically, the smoking rate in this group was approximately 29% in 1992, increasing to 32% in 1997 and subsequently displaying a steady decline in each measurement period, reaching 23% at the most recent assessment conducted in 2017 (overall decline: 5.6%). Conversely, the observed reduction in smoking prevalence among individuals with educational qualifications below the tertiary level displayed a less substantial reduction in overall nicotine consumption. Those with intermediate-level qualifications exhibited a smoking prevalence rate of around 27% in 2017 (significant decline: 2.1%), while individuals with the level of obligatory schooling qualifications demonstrated a somewhat smaller nonsignificant decline (0.6%) in relative smoking rates, with a prevalence rate of 29% in the most recent assessment. Over the years, only minor fluctuations and a slight decrease in the measured values are observed in both groups (intermediate and obligatory schooling qualifications). We cannot explain it why the prevalence is highest among people in the middle education group (SEK 2) (Figure 1). According to international experience (26), it would be expected to have lower prevalence than the lowest level of education, which is not the case in Switzerland. It could be that this group may be targeted by tobacco advertising. But we cannot prove this with data and it would be speculation. Future federal investigations will look into this question in more detail.

In addition, there is a persistently high rate of smoking, particularly among very young people, at least starting from the age of 15 (Figure 3). This is a clear indication that the protection of children and young people is not sufficient. Recent data showed, that the use of tobacco and nicotine products was more common among Swiss adolescents than earlier studies and is more prevalent in Switzerland than in most other high-income countries (27, 28).

We also found that people without a Swiss passport have a significantly higher rate of smoking than people who have a Swiss passport. The proportion in 2017 showed a smoking rate of 32% among people without Swiss citizenship and 26% among people with a Swiss passport (Figure 2). An unusual discrepancy arises from this data. On the one hand, Switzerland has one of the world's top-three-rated healthcare systems (16), which offers all people, including people with low socioeconomic status, high-quality medical care (17). On the other hand, we are facing a significant health inequity where individuals with low or medium levels of educational qualifications are potentially poorly protected from smoking-related nicotine addiction, and with people without a Swiss passport and young people are disproportionately affected. Targeted cessation support for groups with a low socioeconomic background and taxation of tobacco products, smoke-free environments, education campaigns, advertising bans and cessation support may reduce smoke related health inequities and prevalence (29).

The COVID-19 pandemic might provide some parallel explanation. Despite the fact that the Swiss healthcare system extensively focused on the health crisis triggered by SARS-CoV-2, Switzerland saw significantly higher and excess mortality, especially among people without a Swiss passport and those of lower socioeconomic status compared with the general population (30, 31). Such mortality risks associated with social background are well-documented in many countries, especially in minority and disadvantaged populations (32). These effects were observed in countries such as the United States, where there is published evidence of higher health inequity, especially for socially marginalized groups (33). For Switzerland, existing datasets are inadequate in demonstrating potential health inequities, and more studies are needed to show the existence of social inequity linked to health variables in this highly developed country.

Because the level of educational qualifications can be considered a predictor of the rate of smoking, the data should be compared with countries where nicotine consumption through smoking is more strongly restricted. Countries with strong education systems such as Ireland or the United Kingdom, which have some of the strongest tobacco restrictions in Europe, could serve as comparators (10). In Ireland, smoking prevalence has dropped from 41% in 1995 to 13% in 2015 (34). In UK, 13% of people smoked in 2021 (35). The comparison with these two countries shows a higher demand in Switzerland, with a currently estimated smoking rate of about 27% (36).

However, the comparison also shows that existing measures taken in other countries cannot completely negate smoking-related nicotine dependence. For this reason, to further reduce smoking in the population, targeting groups according to educational attainment alone would be insufficient, just as it is probably not enough to rely solely on stricter rules and restrictions related to cigarette sales or consumption to develop a successful prevention policy. There is an interesting example of this, namely in Sweden. Between 2004 and 2021, daily smoking in Sweden decreased from 16 to 6% among men and women (37). What might have caused this significant reduction? One important factor is that smoking rates among Swedish males never reached the extreme levels of other European countries, giving Sweden an advantage in curbing the smoking epidemic (38). A decrease in the number of smokers occurred simultaneously with a decrease in the consumption of snus, a traditional oral tobacco product kept under the upper lip. Hence, even before the sales of snus started to increase from an all-time low sales level in 1970, the male smoking rate was at least 20% lower than in most European countries (39). From the 1980s and onwards, about 30% of adult smoking men who wanted to quit smoking used snus as a way to help them do so, resulting in two-thirds becoming chronically addicted to snus (40). Importantly, women did not use snus to the same extent but still showed the same rate of smoking decrease as men in the period. The sale of snus is banned in the EU, but Sweden was granted an exception, and presently 19% of men and 4% of women use it daily (41). Whether this can be viewed as a success is controversial, and many people are reluctant to name Sweden as a model country for smoking prevention since the low cigarette consumption is accompanied by a steady consumption of a nicotine substitute. In one Norwegian survey in males, the use of snus increased the probability of quitting smoking compared with medicinal nicotine products (42). Even if this is not the perfect policy, it might help reduce health inequity in Sweden at least in part. However, in Sweden, despite successful nicotine policies, people with lower socioeconomic status and lower educational qualification level also showed an increased risk for smoking addiction, as in other countries (43, 44).

Nicotine is among the most potent addictive substances, often producing withdrawal symptoms more severe than those associated with cocaine or heroin (45). In addition to the discussion on health equity and smoking prevalence, there is another important aspect. Smoking is also disproportionately associated with people with psychiatric disorders. Many studies report a positive association between smoking and mental illness, with smoking rates increasing with the severity of the disease. Individuals with mental illness also tend to start smoking at a younger age, smoke more heavily, and are more addicted to cigarettes than the general population (46). For these reasons, discussions should not only address which political and preventive strategies are the strictest or the best. Above all, we should be aware that smokers as individuals are often severely addicted and that a sole focus on education alone does not go far enough. These people need help if the aim is for them to stop smoking.

This study has numerous limitations. First, the statistical information is based on health surveys that take place every five years in Switzerland and in the given data set, the information regarding education, citizenship and age is split into three separate groups making it impossible to control for these subgroups simultaneously. The apparent decrease in the smoking rate in 2007 for the group with obligatory schooling cannot be explained statistically. Furthermore, our study only shows divergent smoking prevalences in different social groups. In future studies it has to be discussed, whether these groups have differences in morbidity and mortality rates due to smoking. A further limitation lies in the analysis of the prevalence among people without Swiss citizenship. The data employed only provide information on whether or not individuals possess Swiss citizenship. There is a significant part of the population that has only recently obtained citizenship but is not visible in the data used in our analysis. Furthermore, the imprecisely defined and very small proportion of occasional smokers was combined with smokers, as this distinction is rarely differentiated according to the WHO. The last limitation is the sole analysis of the educational qualification level. For a more detailed representation of the different populations, it would be important to analyze the socioeconomic background of smokers and nonsmokers. Unfortunately, such public data does not exist in Switzerland.

## 5 Conclusion

The findings of this study indicate a correlation, that in Switzerland, a high level of educational qualification is correlated with lower levels of smoking. People with lower schooling levels show significantly higher prevalence of smoking. Swiss citizens have a significantly lower prevalence of smoking than non-Swiss citizens. Since Switzerland has the lowest prevention standards and poorly implemented policies against nicotine consumption compared with other countries in Europe, the high smoking rates can potentially lead to longer-term health consequences and high healthcare costs. There is a need to consider how the high smoking prevalence in different groups can be better addressed politically and by adopting the rules of the FCTS. The sole focus on educational attainment does not seem to be a sufficient reference point for prevention policies. This study shows that smoking-related nicotine addiction is complex and that many different aspects, mental health and social determinants come together alongside social and health inequities, which will need multifaceted approach to achieve better long-term outcomes.

## Data availability statement

Publicly available datasets were analyzed in this study. This data can be found here: https://www.bfs.admin.ch/bfs/de/home.html.

## Author contributions

DW: Data curation, Formal analysis, Investigation, Project administration, Validation, Writing—original draft. HG: Investigation, Methodology, Supervision, Writing—original draft, Conceptualization, Validation. DK: Formal analysis, Methodology, Validation, Writing—review & editing, Conceptualization. SM: Formal analysis, Methodology, Validation, Writing— review & editing, Conceptualization. TS: Formal analysis, Methodology, Validation, Writing—review & editing, Conceptualization. SB: Formal analysis, Methodology, Validation, Writing—review & editing, Conceptualization. MH: Formal analysis, Methodology, Validation, Writing—review & editing, Conceptualization. KR: Formal analysis, Methodology, Validation, Writing—review & editing. AO: Formal analysis, Methodology, Validation, Writing—review & editing. PW: Formal analysis, Methodology, Validation, Writing—review & editing. DH: Formal analysis, Methodology, Validation, Writing—review & editing. RB-H: Formal analysis, Methodology, Validation, Writing—review & editing. JF: Formal analysis, Methodology, Validation, Writing —review & editing. GF: Formal analysis, Methodology, Validation, Writing—review & editing. JM: Formal analysis, Methodology, Validation, Writing—review & editing, Conceptualization. TP: Formal analysis, Methodology, Validation, Writing—review & editing, Conceptualization. AG: Conceptualization, Data curation, Formal analysis, Writing—original draft, Investigation, Methodology, Project administration, Resources, Supervision, Validation, Visualization.
