# Supplementary material for: Smoking trends and health equity in Switzerland between 1992 and 2017: dependence of smoking prevalence on educational level and social determinants
Source: Front Psychiatry. 2023 Nov 23;14:1258272. doi: 10.3389/fpsyt.2023.1258272 (PMC10701586; doi:10.3389/fpsyt.2023.1258272)
Supplement: Supplementary file 1 [file Data_Sheet_1.PDF]

Schweizerische Eidgenossenschaft
Confédération suisse
Confederazione Svizzera
Confederaziun svizra

Eidgenössisches Departement des Innern EDII
Bundesamt für Statistik BFS
Abteilung Gesundheit und Soziales

Schweizerische Gesundheitsbefragung 2017
Enquête suisse sur la santé 2017
Indagine sulla salute in Svizzera 2017

Telefonischer und schriftlicher Fragebogen

Sektion Gesundheit der Bevölkerung
CH-2010 Neuchâtel
Espace de l'Europe 10

26.10.2020

Source: https://www.bfs.admin.ch/bfs/de/home/statistiken/gesundheit/erhebungen/sgb.assetdetail.14839331.html

Telefonischer Fragebogen

Nr.	Fragen	Thema	Quelle / Bemerkungen
01000	LINK : ALL<br>--<br>Wie ist Ihr Gesundheitszustand im Allgemeinen? Ist er…<br><br>*INT : Antwortmöglichkeiten vorlesen!*<br>--<br>1 - Sehr gut<br>2 - Gut<br>3 - Mittelmässig<br>4 - Schlecht<br>5 - Sehr schlecht<br>--<br>(-1) - Weiss nicht<br>(-2) - Keine Antwort	Selbstwahrgenommener Gesundheitszustand<br><br><br>TSUBG05	EHIS (MEHM1)<br>*SGB92-02: TSUBG01*<br>*SGB07: TSUBG03*<br>*SGB12: TSUBG04*<br>*SGB17: Antwortmodalität 3 in Italienisch und Französisch gegenüber 2012 angepasst.*
01100	LINK : ALL<br>--<br>Haben Sie eine Krankheit oder ein gesundheitliches Problem, wo chronisch oder andauernd ist? Damit meine ich Krankheiten oder gesundheitliche Probleme, wo schon seit mindestens 6 Monaten andauern oder schätzungsweise noch während mindestens 6 Monaten andauern werden.<br>--<br>1 - Ja<br>2 - Nein<br>--<br>(-1) - Weiss nicht<br>(-2) - Keine Antwort	Chronische Krankheiten<br><br><br>TKRAN07	EHIS 2008 (MEHM2)<br>*SGB97-02 : TKRAN01*<br>*SGB07 :TKRAN04*<br>*SGB12 : TKRAN07*
01200	LINK : ALL<br>--<br>Seit mindestens 6 Monaten, wie sehr sind Sie eingeschränkt durch ein gesundheitliches Problem bei gewöhnlichen Aktivitäten im täglichen Leben? Würden Sie sagen, Sie sind…<br><br>*INT : Antwortmöglichkeiten vorlesen!*<br>--<br>1 - Stark eingeschränkt<br>2 - Eingeschränkt, aber nicht stark<br>3 - Überhaupt nicht eingeschränkt → **012.20**<br>--<br>(-1) - Weiss nicht → **012.20**<br>(-2) - Keine Antwort → **012.20**	Chronische Krankheiten<br><br><br>TKRAN08	EHIS 2008 (MEHM3)<br>*SGB97-02 : TKRAN02*<br>*SGB07 :TKRAN05*<br>*SGB12 : TKRAN08*
01210	LINK : IF 01200={1,2}	Chronische Krankheiten	ESS 1997

	---<br>Ist das gesundheitliche Problem Ihrer Meinung nach körperlich, psychisch oder geistig bedingt?<br><br>---<br>1 - Körperlich bedingt<br>2 - Psychisch (oder geistig) bedingt<br>3 - Beides (körperlich UND psychisch oder geistig)<br>4 - Weder körperlich noch psychisch (oder geistig)<br>---<br>(-1) - Weiss nicht<br>(-2) - Keine Antwort	TKRAN09	*SGB97-02: TKRAN03a-b*<br>*SGB07-12: TKRAN06*<br>*SGB17: Antwortmodalitäten angepasst und Umfang um "geistig" ausgedehnt.*
01220	LINK : ALL<br>---<br>Haben Sie in den letzten 12 Monaten eine von den folgenden Krankheiten oder gesundheitlichen Problemen gehabt?<br><br>a) Asthma<br>b) Allergien wie Heuschnupfen, Hautallergien (Dermatitis), Lebensmittelallergie etc..<br>c) Arthrose, Arthritis<br>d) Osteoporose<br>e) Chronische Bronchitis, COPD (chronisch obstruktiven Lungenkrankheit), Emphysem (Lungenblähung)<br>f) Herzinfarkt<br>g) Schlaganfall (Gehirnblutung, zerebrales Blutgerinnsel)<br>h) Krebs<br>i) Depression<br>i) Harninkontinenz (Probleme, die Blase zu kontrollieren)<br><br>*INT : Items vorlesen!*<br>---<br>1 - Ja<br>2 - Nein<br>---<br>(-1) - Weiss nicht<br>(-2) - Keine Antwort	Chronische Krankheiten<br><br><br>TKRAN10a<br>TKRAN10b<br>TKRAN10c<br>TKRAN10d<br>TKRAN10e<br>TKRAN10f<br>TKRAN10g<br>TKRAN10h<br>TKRAN10i<br>TKRAN10j	EHIS 2014 (angepasst)<br>*SGB17: Fragen vom schriftlichen Fragebogen im telefonischen Fragebogen verschoben*
01221	LINK : IF 01220_a={1}<br>---<br>Ist das Asthma von einem Arzt oder einer anderen Person aus dem medizinischen Bereich diagnostiziert worden?<br>---<br>1 - Ja<br>2 - Nein	Chronische Krankheiten<br><br>TKRAN11a	EHIS 2014 (angepasst)<br>*SGB17: Frage vom schriftlichen Fragebogen im telefonischen Fragebogen verschoben*

	---<br>(-1) - Weiss nicht<br>(-2) - Keine Antwort		
01222	LINK : IF 01220_b={1}<br>---<br>Ist die Allergie von einem Arzt oder einer anderen Person aus dem medizinischen Bereich diagnostiziert worden?<br>---<br>1 - Ja<br>2 - Nein<br>---<br>(-1) - Weiss nicht<br>(-2) - Keine Antwort	Chronische Krankheiten<br><br>TKRAN11b	EHIS 2014 (angepasst)<br>*SGB17: Frage vom schriftlichen Fragebogen im telefonischen Fragebogen verschoben*
01223	LINK : IF 01220_c={1}<br>---<br>Ist die Arthrose / Arthritis von einem Arzt oder einer anderen Person aus dem medizinischen Bereich diagnostiziert worden?<br>---<br>1 - Ja<br>2 - Nein<br>---<br>(-1) - Weiss nicht<br>(-2) - Keine Antwort	Chronische Krankheiten<br><br>TKRAN11c	EHIS 2014 (angepasst)<br>*SGB17: Frage vom schriftlichen Fragebogen im telefonischen Fragebogen verschoben*
01224	LINK : IF 01220_d={1}<br>---<br>Ist die Osteoporose von einem Arzt oder einer anderen Person aus dem medizinischen Bereich diagnostiziert worden?<br>---<br>1 - Ja<br>2 - Nein<br>---<br>(-1) - Weiss nicht<br>(-2) - Keine Antwort	Chronische Krankheiten<br><br>TKRAN11d	EHIS 2014 (angepasst)<br>*SGB17: Frage vom schriftlichen Fragebogen im telefonischen Fragebogen verschoben*
01225	LINK : IF 01220_e={1}<br>---<br>Ist die Chronische Bronchitis, COPD oder das Emphysem von einem Arzt oder einer anderen Person aus dem medizinischen Bereich diagnostiziert worden?<br>---<br>1 - Ja<br>2 - Nein	Chronische Krankheiten<br><br>TKRAN11e	EHIS 2014 (angepasst)<br>*SGB17: Frage vom schriftlichen Fragebogen im telefonischen Fragebogen verschoben*

	--<br>(-1) - Weiss nicht<br>(-2) - Keine Antwort		
01226	LINK : IF 01220_f={1}<br>--<br>Ist der Herzinfarkt von einem Arzt oder einer anderen Person aus dem medizinischen Bereich diagnostiziert worden?<br>--<br>1 - Ja<br>2 - Nein<br>--<br>(-1) - Weiss nicht<br>(-2) - Keine Antwort	Chronische Krankheiten<br><br>TKRAN11f	EHIS 2014 (angepasst)<br>*SGB17: Frage vom schriftlichen Fragebogen im telefonischen Fragebogen verschoben*
01227	LINK : IF01220_g={1}<br>--<br>Ist der Schlaganfall von einem Arzt oder einer anderen Person aus dem medizinischen Bereich diagnostiziert worden?<br>--<br>1 - Ja<br>2 - Nein<br>--<br>(-1) - Weiss nicht<br>(-2) - Keine Antwort	Chronische Krankheiten<br><br>TKRAN11g	EHIS 2014 (angepasst)<br>*SGB17: Frage vom schriftlichen Fragebogen im telefonischen Fragebogen verschoben*
01228	LINK : IF 01220_h={1}<br>--<br>Ist der Krebs von einem Arzt oder einer anderen Person aus dem medizinischen Bereich diagnostiziert worden?<br>--<br>1 - Ja<br>2 - Nein<br>--<br>(-1) - Weiss nicht<br>(-2) - Keine Antwort	Chronische Krankheiten<br><br>TKRAN11h	EHIS 2014 (angepasst)<br>*SGB17: Frage vom schriftlichen Fragebogen im telefonischen Fragebogen verschoben*
01229	LINK : IF 01220_i={1}<br>--<br>Ist die Depression von einem Arzt oder einer anderen Person aus dem medizinischen Bereich diagnostiziert worden?<br>--<br>1 - Ja<br>2 - Nein<br>--<br>(-1) - Weiss nicht<br>(-2) - Keine Antwort	Chronische Krankheiten<br><br>TKRAN11i	EHIS 2014 (angepasst)<br>*SGB17: Frage vom schriftlichen Fragebogen im telefonischen Fragebogen verschoben*

01230	LINK : IF 01220_j={1}<br>--<br>Ist die Harninkontinenz von einem Arzt oder einer anderen Person aus dem medizinischen Bereich diagnostiziert worden?<br>--<br>1 - Ja<br>2 - Nein<br>--<br>(-1) - Weiss nicht<br>(-2) - Keine Antwort	Chronische Krankheiten<br><br>TKRAN11j	EHIS 2014 (angepasst)<br>*SGB17: Frage vom schriftlichen Fragebogen im telefonischen Fragebogen verschoben*
01310	LINK : ALL<br>--<br>Sehen Sie genug, um ein Buch oder eine Zeitung zu lesen?<br>Mit Brille oder Kontaktlinsen, wenn Sie das normalerweise tragen.<br><br>*INT : Nachfragen, wenn die Person nur mit "Ja" antwortet*<br>--<br>1 - Ja, ohne Schwierigkeiten<br>2 - Ja, mit leichten Schwierigkeiten<br>3 - Ja, aber mit starken Schwierigkeiten<br>4 - Nein<br>--<br>(-2) - Keine Antwort	Behinderung: Sehen<br><br>TSBHD03	INSEE SYIN 12<br>IGIP 65.01
01400	LINK : ALL<br>--<br>Tragen Sie ein Hörgerät?<br><br>*INT : z.B. in den Taschen, hinter dem Ohr, im Ohr, im Kanal, oder ein Cochlea-Implantat*<br>--<br>1 - Ja<br>2 - Nein<br>--<br>(-2) - Keine Antwort	Behinderung: Hören<br><br>THBHD01	BFS - SGB<br>EHIS modifiziert (keine Rubrik „taub“)<br><br>*EHIS modifiziert (keine Rubrik „taub“)*
01410	LINK : ALL<br>--<br>Können Sie einem gewöhnlichen Gespräch, wo 2 weitere Personen teilnehmen, folgen?<br><br>*INT : Allenfalls mit Hörgerät.*<br>*Nachfragen, wenn die Person nur mit "Ja" antwortet*	Behinderung: Hören<br><br>THBHD02	BFS - SGB

	---<br>1 - Ja, ohne Schwierigkeiten<br>2 - Ja, mit leichten Schwierigkeiten<br>3 - Ja, aber mit starken Schwierigkeiten<br>4 - Nein<br>---<br>(-2) - Keine Antwort		
01500	LINK : ALL<br>---<br>Können Sie ohne Schwierigkeiten reden (sprechen)?<br><br>*INT : Nachfragen, wenn die Person nur mit "Ja" antwortet*<br>---<br>1 - Ja, ohne Schwierigkeiten<br>2 - Ja, mit leichten Schwierigkeiten<br>3 - Ja, aber mit starken Schwierigkeiten<br>4 - Nein<br>---<br>(-2) - Keine Antwort	Behinderung: Reden<br><br><br>TRBHD03	BFS - SGB<br>*SGB97-07: TRBHD01*<br>*SGB12: TRBHD03*
01600	LINK : ALL<br>---<br>Wie weit können Sie alleine d.h. ohne Hilfe laufen (gehen), ohne dass Sie anhalten müssen und ohne dass Sie starke Beschwerden haben?<br><br>*INT : Antwortmöglichkeiten vorlesen!*<br>---<br>1 - 200 Meter oder mehr<br>2 - Mehr als einige Schritte, aber weniger als 200 Meter<br>3 - Nur einige Schritte<br>4 - Kann überhaupt nicht gehen<br>---<br>(-2) - Keine Antwort	Behinderung: Bewegung<br><br><br>TBBHD03	WHO Survey 4.2.1
01650	LINK : ALL<br>---<br>Haben Sie Schwierigkeiten, sich mehr als 10 Minuten zu konzentrieren?<br><br>*INT : Nachfragen, wenn die Person nur mit "Ja" antwortet*<br>---<br>1 - Nein, keine Schwierigkeiten<br>2 - Ja, leichte Schwierigkeiten	Behinderung:<br>Konzentration<br><br><br>TKBHD01	INSEE (F)- Enquête vie quotidienne et santé 2014 (angepasst)

	3 - Ja, grosse Schwierigkeiten<br>4 - Kann mich überhaupt nicht konzentrieren<br>---<br>(-2) - Keine Antwort		
01651	LINK : ALL<br>---<br>Haben Sie Schwierigkeiten, sich an wichtige Sachen (Dinge) zu erinnern?<br><br>*INT : Nachfragen, wenn die Person nur mit "Ja" antwortet*<br>---<br>1 - Nein, keine Schwierigkeiten<br>2 - Ja, leichte Schwierigkeiten<br>3 - Ja, grosse Schwierigkeiten<br>4 - Kann mich überhaupt nicht erinnern<br>---<br>(-2) - Keine Antwort	Behinderung: Sich Erinnern<br><br><br>TKBHD02	INSEE (F)- Enquête vie quotidienne et santé 2014 (angepasst)<br>*SGB02: TEBHD01*
01800	LINK : (IF ALTER>=65) OR (IF ALTER<65 AND (01200={1,2} OR 01310={2,3,4} OR 01410={2,3,4} OR 01500={2,3,4} OR 01600={2,3,4}))<br><br>Nur an Personen ab 65 Jahren. Bei Personen unter 65 Jahren nur an Personen mit Behinderungen (TKRAN08/12.00 = 1,2 oder TSBHD03/13.10=2, 3, 4 oder THBHD02/14.10=2, 3, 4 oder TRBHD03/15.00=2, 3, 4 oder TBBHD03/16.00=2, 3, 4) Alle anderen Personen unter 65 Jahren weiter zur Frage 18.20<br>---<br>Ich lese Ihnen jetzt verschiedene Alltagsaktivitäten vor. Bitte sagen Sie mir jedes Mal, ob Sie das ohne Schwierigkeiten, mit leichten Schwierigkeiten, mit starken Schwierigkeiten oder überhaupt nicht machen können<br><br>a) Selbstständig essen<br>b) Selbstständig ins oder aus dem Bett steigen oder von einem Sessel aufstehen<br>c) Selbstständig an- und ausziehen<br>d) Selbstständig zur Toilette gehen<br>e) Selbstständig baden oder duschen<br><br>*INT : Items vorlesen!*<br>*Vorübergehende Gesundheitsprobleme nicht einbeziehen!*<br>*Falls nötig: Präzisieren, dass die Schwierigkeiten im Zusammenhang mit dem Gesundheitszustand stehen müssen.*<br>---<br>1 - Ja, ohne Schwierigkeiten<br>2 - Ja, mit leichten Schwierigkeiten<br>3 - Ja, aber mit starken Schwierigkeiten<br>4 - Nein, überhaupt nicht<br>---<br>(-2) - Keine Antwort	Behinderung: ADL Basis Alltägliche Aktivität<br><br><br>TBADL02a<br>TBADL02b<br>TBADL02c<br>TBADL02d<br>TBADL02e	EHIS<br>*EHIS Antwortskala angepasst*<br>*SGB07: TBADL01a-e*

01810	LINK : (IF ALTER>=65) OR (IF ALTER<65 AND (01200={1,2} OR 01310={2,3,4} OR 01410={2,3,4} OR 01500={2,3,4} OR 01600={2,3,4}))<br>---<br>Ich lese Ihnen jetzt andere Alltagsaktivitäten vor. Bitte sagen Sie mir jedes Mal, ob Sie das ohne Schwierigkeiten, mit leichten Schwierigkeiten, mit starken Schwierigkeiten oder überhaupt nicht machen können.<br><br>a) Selbstständig Essen zubereiten<br>b) Selbstständig telefonieren<br>c) Selbstständig einkaufen<br>d) Selbstständig Wäsche waschen<br>e) Selbstständig leichte Hausarbeit erledigen<br>f) Selbstständig gelegentlich schwere Hausarbeit erledigen<br>g) Sich selbstständig um Finanzen kümmern<br>h) Selbstständig die öffentlichen Verkehrsmittel benützen<br><br>*INT : Items vorlesen!*<br>*Vorübergehende Gesundheitsprobleme nicht einbeziehen!*<br>*Schwere Hausarbeit = schwere Möbel verschieben, Frühjahrputz, Boden nass aufnehmen, Fenster putzen, etc.*<br>*Falls nötig: Präzisieren, dass die Schwierigkeiten im Zusammenhang mit dem Gesundheitszustand stehen müssen.*<br>---<br>1 - Ja, ohne Schwierigkeiten<br>2 - Ja, mit leichten Schwierigkeiten<br>3 - Ja, aber mit starken Schwierigkeiten<br>4 - Nein, überhaupt nicht<br>---<br>(-2) - Keine Antwort	Behinderung: IADL Instrumentalische Alltägliche Aktivität<br><br><br>TIADL02a<br>TIADL02b<br>TIADL02c<br>TIADL02d<br>TIADL02e<br>TIADL02f<br>TIADL02g<br>TIADL02h	EHIS<br>*EHIS Antwortskala angepasst*<br>*SGB07:TIADL01a-h*
01820	LINK : IF ALTER <65 AND (01200={3,-1,-2} AND 01310={1,-2} AND 01410={1,-2} AND 01500={1,-2} AND 01600={1,-2})<br><br>Nur an unter 65 Jährige, welchen die Frage 18.00 nicht gestellt wurde.<br>---<br>Können Sie ohne Schwierigkeiten Selbstständig die öffentlichen Verkehrsmittel benützen?<br><br>*INT : Nachfragen, wenn die Person nur mit "Ja" antwortet*<br>---<br>1 - Ja, ohne Schwierigkeiten<br>2 - Ja, mit leichten Schwierigkeiten<br>3 - Ja, aber mit starken Schwierigkeiten<br>4 - Nein, überhaupt nicht<br>---<br>(-2) - Keine Antwort	Behinderung: IADL Instrumentalische Alltägliche Aktivität<br><br><br>TIADL02hh	EHIS<br>*EHIS Antwortskala angepasst*<br>*SGB07:TIADL01h*
02100	LINK : ALL	Krankheitssymptome	IGIP tél. 5.01-5.11<br>*SGB12: TKRSY34 eingefügt*

	--<br>Ich lese Ihnen jetzt verschiedene Beschwerden vor. Bitte sagen Sie mir jedes Mal, ob Sie das in den letzten 4 Wochen überhaupt nicht, ein bisschen oder stark gehabt haben:<br><br>a) Rücken- oder Kreuzschmerzen<br>b) Allgemeine Schwäche, Müdigkeit, Energielosigkeit<br>c) Schmerzen oder Druckgefühl im Bauch<br>d) Durchfall, Verstopfung oder beides<br>e) Einschlaf- oder Durchschlafstörungen<br>f) Kopfschmerzen, Druck im Kopf oder Gesichtsschmerzen<br>g) Herzklopfen, Herzjagen oder Herzstolpern<br>h) Schmerzen oder Druck im Brustbereich<br>i) Fieber<br>j) Schmerzen in den Schultern, im Nacken und/oder in den Armen<br><br>*INT : Items vorlesen!*<br>*Wenn zurzeit gegen eine Beschwerde ein Medikament genommen wird, heisst das, dass man diese Beschwerde hat.*<br>--<br>1 - Überhaupt nicht<br>2 - Ein bisschen<br>3 - Stark<br>--<br>(-2) - Keine Antwort	TKRSY01<br>TKRSY02<br>TKRSY03<br>TKRSY04<br>TKRSY05<br>TKRSY06<br>TKRSY07<br>TKRSY08<br>TKRSY09<br>TKRSY34	
02101	LINK : IF 02100_j={2,3}<br><br>Nur an Personen mit Schmerzen in den Schultern, im Nacken oder in den Armen (TKRSY34/21.00= 2, 3)<br>--<br>Sind Sie der Ansicht, dass die Schmerzen in den Schultern, im Nacken und/oder in den Armen in Zusammenhang mit Ihrer jetzigen oder früheren Arbeit stehen?<br><br>*INT : Antwortmöglichkeiten vorlesen!*<br>--<br>1 - Ja, sicher<br>2 - Ja, eher<br>3 - Teilweise<br>4 - Nein, eher nicht<br>5 - Nein, sicher nicht<br>--<br>(-1) - Weiss nicht<br>(-2) - Keine Antwort	Krankheitssymptome<br><br><br>TKRSY36	SECO (M.Graf)

02102	LINK : IF 02100_a={2,3}<br><br>Nur an Personen mit Rücken- oder Kreuzschmerzen (TKRSY01/21.00= 2, 3)<br>---<br>Bei den Rücken- oder Kreuzschmerzen, sind Sie der Ansicht, dass diese in Zusammenhang mit Ihrer jetzigen oder früheren Arbeit stehen?<br><br>*INT : Antwortmöglichkeiten vorlesen!*<br>---<br>1 - Ja, sicher<br>2 - Ja, eher<br>3 - Teilweise<br>4 - Nein, eher nicht<br>5 - Nein, sicher nicht<br>---<br>(-1) - Weiss nicht<br>(-2) - Keine Antwort	Krankheitssymptome<br><br><br>TKRSY35	BFS - SGB
02200	LINK : IF SEX=2 AND ALTER>=15 AND ALTER <=49<br><br>Nur an Frauen zwischen 15 und 49 Jahren . Frauen ab 50 und Männer weiter zu 23.00<br>---<br>Sind Sie zurzeit schwanger?<br>---<br>1 - Ja<br>2 - Nein<br>---<br>(-1) - Weiss nicht<br>(-2) - Keine Antwort	Gesundheitszustand<br>Schwangerschaft<br><br><br>TSCHW01	BFS – SGB
02300	LINK : ALL<br><br>Wieder an alle<br>---<br>Können Sie mir sagen, wie gross Sie ohne Schuhe sind?<br><br><xxx> - Zentimeter<br>---<br>(-1) - Weiss nicht<br>(-2) - Keine Antwort	Gesundheitszustand<br>Körpermasse<br><br><br>TGEZU01	IGIP tél. 54.00
02310	LINK : IF (SEX=1) OR (SEX=2 AND ALTER<=49 AND 02200={2,-1,-2}) OR (SEX=2 AND ALTER>=50)<br>---<br>Und wie schwer sind Sie ohne Kleider?	Gesundheitszustand<br>Körpermasse	IGIP tél. 55.00<br>*SGB17: TGEZU02c und TGEZU02d =TGEZU02*

	<xxx> - Kilos<br>---<br>(-1) - Weiss nicht<br>(-2) - Keine Antwort	TGEZU02<br>[TGEZU02c]	
02311	LINK : IF 02200={1}<br><br>Bei schwangeren Frauen (TSCHW01/22.00 = 1)<br>---<br>Wie schwer sind Sie am Anfang von der Schwangerschaft gewesen?<br><br><xxx> - Kilos<br>---<br>(-1) - Weiss nicht<br>(-2) - Keine Antwort	Gesundheitszustand<br>Körpermasse<br><br>[TGEZU02d]	BFS – SGB<br>*SGB17: TGEZU02c und TGEZU02d =TGEZU02*
02400<br><br>*PROXY*	LINK : ALL AND NOT PROXY<br>---<br>Sind Sie im Moment mit Ihrem Körpergewicht zufrieden? Sind Sie...<br><br>*INT : Antwortmöglichkeiten vorlesen!*<br>---<br>1 - Absolut zufrieden<br>2 - Ziemlich zufrieden<br>3 - Eher unzufrieden<br>4 - Absolut unzufrieden<br>---<br>(-2) - Keine Antwort	Gesundheitszustand<br>Zufriedenheit mit Gewicht<br><br>TGEZU03	"Etude sur la santé des adolescents" / Michaud
02410<br><br>*PROXY*	LINK : ALL AND NOT PROXY<br>---<br>Möchten Sie Ihr Gewicht verändern?<br><br>*INT : Antwortmöglichkeiten vorlesen!*<br>---<br>1 - Ja, abnehmen<br>2 - Ja, zunehmen<br>3 - Nein<br>---<br>(-2) - Keine Antwort	Gesundheitszustand<br>Zufriedenheit mit Gewicht<br><br>TGEZU07	"Etude sur la santé des adolescents" / Michaud<br>*SGB97 : 15+ Jahre*<br>*SGB02 : 15-24 Jahre*<br>*SGB07 : 15-49 Jahre*<br>*Ab SGB12 : 15+ Jahre*<br>*SGB17: les variables TGEZU04+05 (présentes de 1997 à 2012) ont été combinées pour n'avoir qu'une seule question*
02500	LINK : ALL AND NOT PROXY	Psychische Gesundheit	MOS SF-36 MHI-5

PROXY	---<br>Ich lese Ihnen jetzt verschiedene Gefühlzustände vor. Bitte sagen Sie mir jedes Mal, ob Sie sich so in den letzten 4 Wochen immer, meistens, manchmal, selten oder nie gefühlt haben:<br><br>1) Sehr nervös<br>2) So niedergeschlagen oder verstimmt, dass Sie nichts hat können aufmuntern<br>3) Ruhig, ausgeglichen und gelassen<br>4) Entmutigt und deprimiert<br>5) Glücklich<br><br>*INT : Items vorlesen!*<br>---<br>1 - Immer<br>2 - Meistens<br>3 - Manchmal<br>4 - Selten<br>5 - Nie<br>---<br>(-2) - Keine Antwort	TPSYG11<br>TPSYG12<br>TPSYG13<br>TPSYG14<br>TPSYG15	*SGB12 : changement de nom de variables pour TPSYG11-15 car les questions combinaient 2 indices (EVI et MHI-5) qui ne se suivaient pas et étaient mélangées, les réponses pouvaient s'influencer (TPSYG17,18.19,21,23)*<br>*SGB17 ist gleich wie SGB07*
02600	LINK : ALL<br>---<br>Sind Sie in den letzten 12 Monaten bei einem Arzt gewesen, inklusive Spezialisten und Gynäkologen, den Zahnarzt nicht mitgerechnet?<br><br>*INT : Alle Besuche in der Arztpraxis mit Untersuchungen, Beratungen und Therapien. Hausbesuche vom Arzt zählen auch, ausschliessliche Telefonkonsultation zählt nicht*<br>---<br>1 - Ja<br>2 - Nein **→ 026.10**<br>---<br>(-2) - Keine Antwort **→ 026.10**	Inanspruchnahme mediz. ambul. Dienstleistungen<br><br><br>TINAN31	Anal. IGIP tél. 16.00<br>*SGB17: Filter nach Geschlecht: Bei den Männern werden die Gynäkologen nicht erwähnt.*<br>*SGB92:TINAN01*<br>*SGB97-02:TINAN50*<br>*Ab SGB07:TINAN31*
02620<br><br><br>*PROXY*	LINK : IF 02600={1} AND NOT PROXY<br><br>Nur an Personen, die in den letzten 12 Monaten beim Arzt waren (TINAN31/02600=1)<br>---<br>Wie häufig sind Sie in den letzten 12 Monaten bei einem Allgemeinpraktiker oder bei Ihrem Hausarzt gewesen?<br><br><0> - Nie<br><xx> - Mal (1 bis 97 Mal) --> 02630<br><br>*INT : besser eine Schätzung als weiss nicht!*	Inanspruchnahme mediz. ambul. Dienstleistungen<br><br><br>TINAN55	BFS - SGB<br>*SGB12: ohne vorangehende Filterfrage*<br>*SGB07: TINAN32*<br>*SGB12: TINAN42*

	---<br>(-1) - Weiss nicht<br>(-2) - Keine Antwort		
02610	LINK : IF 02600={2,-2} OR 02620={0,-1,-2} OR (02600={1} AND PROXY)<br><br>Falls TINAN31=2, -2 /02600=Nein, NR oder wenn TINAN55=0, -1, -2/02620=0, -1, -2.<br>die anderen weiter zu 02630<br>---<br>Haben Sie einen persönlichen Hausarzt resp. eine persönliche Hausärztin?<br><br>*INT : Hausarzt: ein Arzt, zu dem Sie mit den meisten Gesundheitsproblemen gehen können*<br>---<br>1 - Ja<br>2 - Nein<br>---<br>(-2) - Keine Antwort	Inanspruchnahme mediz. ambul. Dienstleistungen<br><br>TINAN56	BFS - SGB<br>*SGB17: population de référence différente et question légèrement adaptée*<br>*SGB97-12: TINAN13*
02630<br><br>*PROXY*	LINK : IF 02600={1} AND SEX={1} AND NOT PROXY<br><br>Nur Männer, die in den letzten 12 Monaten einen Arzt konsultiert haben (sex=1 and TINAN31/02600 = 1). Frauen, die in den letzten 12 Monaten einen Arzt konsultiert haben (sex=2 and TINAN31/02600=1) weiter zu Frage 02640<br>Männer und Frauen, die in den letzten 12 Monaten keinen Arztbesuch hatten (oder keine Antwort) (TINAN31/02600=2, -2), weiter zu 02660<br>---<br>Wie häufig sind Sie in den letzten 12 Monaten bei einem Spezialisten gewesen?<br><br><xx> - Mal (0 bis 97 Mal)<br><br>*INT : Alle Besuche in der Arztpraxis mit Untersuchungen, Beratungen und Therapien. Keine Zahnarztbesuche! Hausbesuche vom Arzt zählen auch, ausschliessliche Telefonkonsultation zählt nicht. Besser eine Schätzung als weiss nicht!*<br>---<br>(-1) - Weiss nicht<br>(-2) - Keine Antwort	Inanspruchnahme mediz. ambul. Dienstleistungen<br><br>TINAN16	BFS - SGB
02640<br><br>*PROXY*	LINK : IF 02600={1} AND SEX={2} AND NOT PROXY<br><br>Nur Frauen, die in den letzten 12 Monaten einen Arzt konsultiert haben (sex=2 and TINAN31/02600 = 1).<br>Männer, die in den letzten 12 Monaten einen Arzt konsultiert haben (sex=1 and TINAN31/02600=1) weiter zu Frage 02660<br>---<br>Wie häufig sind Sie in den letzten 12 Monaten beim Frauenarzt gewesen?<br><br><xx> - Mal (0 bis 97 Mal)	Inanspruchnahme mediz. ambul. Dienstleistungen<br><br>TINAN17	BFS - SGB

	INT : Besser eine Schätzung als weiss nicht!<br>---<br>(-1) - Weiss nicht<br>(-2) - Keine Antwort		
02650<br><br>*PROXY*	LINK : IF 02600={1} AND SEX={2} AND NOT PROXY<br>---<br>Wie häufig sind Sie in den letzten 12 Monaten bei einem anderen Spezialisten ausser dem Frauenarzt gewesen?<br><br><xx> - Mal (0 bis 97 Mal)<br><br>*INT : Alle Besuche in der Arztpraxis mit Untersuchungen, Beratungen und Therapien. Keine Zahnarztbesuche! Hausbesuche vom Arzt zählen auch, ausschliessliche Telefonkonsultation zählt nicht. Besser eine Schätzung als weiss nicht!*<br>---<br>(-1) - Weiss nicht<br>(-2) - Keine Antwort	Inanspruchnahme mediz. ambul. Dienstleistungen<br><br><br>TINAN18	BFS - SGB
02660<br><br>*PROXY*	LINK : ALL AND NOT PROXY<br><br>Wieder an Alle<br>---<br>Wann haben Sie zum letzten Mal einen Arzt besucht?<br><br><xx> - Monat (TINAN05b)<br><xxxx> - Jahr (TINAN05c)<br><br>*INT : Alle Besuche in der Arztpraxis mit Untersuchungen, Beratungen und Therapien bei Hausärzten, Allgemeinpraktikern, Frauenärzten oder anderen Spezialisten. Keine Zahnarztbesuche! Hausbesuche vom Arzt zählen auch, ausschliessliche Telefonkonsultation zählt nicht. Besser eine Schätzung als weiss nicht!*<br>---<br>0 - Nie<br>---<br>(-1) - Weiss nicht<br>(-2) - Keine Antwort	Inanspruchnahme mediz. ambul. Dienstleistungen<br><br><br>TINAN05b<br>TINAN05c	BFS - SGB
02680<br><br>*PROXY*	LINK : IF SEX={2} AND (02600={2,-2} OR 02640={0,-1,-2}) AND NOT PROXY<br><br>Nur an Frauen, die in den letzten 12 Monaten keinen Arzt (TINAN31/02600 = 2, -2) oder keinen Frauenarzt (TINAN17/02640 = 0, -1, -2) besucht haben (+WN / KA) (TINAN17/02640 = 0. -1, -2).<br>Alle anderen Frauen sowie alle Männer weiter zur Frage 3500<br>---<br>Wann sind Sie zum letzten Mal bei einem Frauenarzt gewesen?	Inanspruchnahme mediz. ambul. Dienstleistungen<br><br><br>TINAN57	Registre des Tumeurs GE<br>*SGB17: Antwortskategorien angepasst*<br>*SGB02-12: TINAN27*

	INT : Antwortmöglichkeiten vorlesen!<br>---<br>1 - Vor 1 Jahr bis weniger als 2 Jahren<br>2 - Vor 2 Jahren bis weniger als 3 Jahren<br>3 - Vor 3 Jahren oder mehr<br>4 - Nie<br>---<br>(-1) - Weiss nicht<br>(-2) - Keine Antwort		
03500<br><br>*PROXY*	LINK : ALL AND NOT PROXY<br>---<br>Sind Sie in den letzten 12 Monaten in Behandlung gewesen wegen einem psychischen Problem?<br>---<br>1 - Ja<br>2 - Nein → **027.00**<br>---<br>(-2) - Keine Antwort → **027.00**	Psychische Gesundheit<br>Inanspruchnahme<br><br>TPSYG08	BFS - SGB
03510<br><br>*PROXY*	LINK : IF 03500={1} AND NOT PROXY<br>---<br>Was ist die Person von Beruf gewesen, wo Sie behandelt hat?<br><br>*INT : 3 Antworten möglich!*<br>---<br>1 - Psychologe, Psychotherapeut<br>2 - Psychiater<br>3 - Allgemeinarzt, Internist<br>4 - Anderer Arzt<br>6 - Komplementärmedizinischer Therapeut (z.B. Naturheilpraktiker, Magnetopath, Ayurveda)<br>7 - Anderer nicht medizinischer Therapeut (z.B. Tanz-, Mal-, Theatertherapeut, Yoga, Schamane)<br>8 - Anderer Beruf<br>---<br>(-1) - Weiss nicht<br>(-2) - Keine Antwort	Psychische Gesundheit<br>Inanspruchnahme<br><br>TPSYG09a<br>TPSYG09b<br>TPSYG09c	BFS - SGB<br>*Ab SGB02 wurde die fünfte Antwortkategorie (anderer medizinischer Spezialist) nicht mehr abgefragt*
02700	LINK : ALL<br><br>WIEDER AN ALLE<br>---<br>Wie viel Mal sind Sie in den letzten 12 Monaten im Spital oder in einer Spezialklinik gewesen, Kuraufenthalte nicht mitgerechnet? Zählen Sie alle Aufenthalte wo Sie übernachtet haben.	Inanspruchnahme<br>Spital<br><br>TINAN35	EHIS<br>*SGB97-02: TINAN23*

	<xxx> - Aufenthalte<br><br>*INT : Eine Überweisung von einem Spital an ein anderes zählt als ein Aufenthalt.*<br>---<br>0 - Nie<br>---<br>(-1) - Weiss nicht → **027.21**<br>(-2) - Keine Antwort → **027.21**		
02710<br><br>*PROXY*	LINK : IF 02700>={1} AND NOT PROXY<br>---<br>Wieviele Tage sind Sie in den letzten 12 Monaten im Spital oder in einer Spezialklinik gewesen, Kuraufenthalte nicht mitgerechnet?<br><br><xxx> - Tage<br><br>*INT : Evtl. beim Schätzen helfen!*<br>---<br>(-1) - Weiss nicht<br>(-2) - Keine Antwort	Inanspruchnahme Spital<br><br>TINAN45	IGIP tél. 18.00<br>*SGB92-02 : TINAN10*<br>*Ab SGB07: andere Referenzpopulation*
02721<br><br>*PROXY*	LINK : ALL AND NOT PROXY<br>---<br>Wie viel Mal sind Sie in den letzten 12 Monaten notfallmässig in einer Notfallstation von einem Spital, in einem Gesundheitszentrum oder in einer Poliklinik gewesen?<br><br><xx> - Mal<br><br>*INT : Evtl. beim Schätzen helfen!*<br>*Zahnbehandlungen gehören nicht dazu*<br>---<br>(-1) - Weiss nicht<br>(-2) - Keine Antwort	Inanspruchnahme Spital<br><br>TINAN58	BAG (F. Gurtner)<br>*SGB07-12: TINAN36-TINAN37*
02722<br><br>*PROXY*	LINK : ALL AND NOT PROXY<br>---<br>Und wie viel Mal sind Sie sonst in den letzten 12 Monaten zur Abklärung oder zur Behandlung in einem Spital oder in einer Spezialklinik gewesen, aber nicht als Notfall und ohne zu Übernachten?<br><br><xx> - Mal<br><br>*INT : Evtl. beim Schätzen helfen!*<br>*Zählen Sie nur Konsultationen, ohne dass Sie übernachtet haben*	Inanspruchnahme Spital<br><br>TINAN59	BAG (F. Gurtner)<br>*SGB17: unterschiedliche Population*<br>*SGB07-12: TINAN38*

	--<br>(-1) - Weiss nicht<br>(-2) - Keine Antwort		
02740	LINK : IF (SEX={2} AND (ALTER>=20 AND ALTER<=49 AND 02200={2,-1,-2}) OR (SEX={2} AND ALTER>={50})<br><br>Frauen ab 20 Jahren und nicht schwanger.<br>--<br>Haben Sie eine Operation zur Entfernung der Gebärmutter gehabt (Hysterektomie)?<br>--<br>1 - Ja<br>2 - Nein<br>--<br>(-1) - Weiss nicht<br>(-2) - Keine Antwort	Inanspruchnahme Operation<br><br><br>TOPER12	STPH (E. Zemp)<br>*SGB17: Adaptation de l'énoncé de la question*<br>*SGB92: SOPER05(a-b)*<br>*SGB97-02: TOPER05(a-b)*<br>*SGB07: TOPER11 (a-b)*
02800	LINK : ALL<br>--<br>Haben Sie in den letzten 7 Tagen irgendein Medikament genommen?<br><br>*INT : Alles - ausser Mittel zur äusseren Anwendungen z.B. Salbe*<br>--<br>1 - Ja<br>2 - Nein **→Frauen ab 50 J. 029.20 →Männer 030.00**<br>--<br>(-2) - Keine Antwort **→Frauen ab 50 J. 029.20 →Männer 030.00**	Medikamentenkonsum<br><br><br>TMEKO01	Anal. IGIP tel. 19.00
02810	LINK : IF SEX={2} AND ALTER <=49 AND 02800={1}<br><br>Nur an Frauen von 15 bis 49 Jahren die in den 7 letzten Tagen ein Medikament genommen haben. Frauen ab 50 Jahren, die ein Medikament genommen haben (tmeko01/28.00 = 1), weiter zu Frage 28.20<br>--<br>Nehmen Sie zurzeit die Pille zur Empfängnisverhütung (Antibabypille)?<br>--<br>1 - Ja<br>2 - Nein **→ 028.20**<br>--<br>(-2) - Keine Antwort **→ 028.20**	Medikamentenkonsum<br><br><br>TMEKO49	BFS - SGB<br>*SGB17: unterschiedliche Referenzpopulation*<br>*SGB12: TMEKO46*
02811	LINK : IF 02810={1}<br><br>Nur an Frauen von 15 bis 49 Jahren, die ein Medikament genommen haben (tmeko01/28.00 =1). Frauen von 15 bis 49 Jahren ohne Medikamente (+ka) (tmeko01/ 28.00 =2, -2), weiter zur Frage 29.20	Medikamentenkonsum<br><br><br>TMEKO47	BFS - SGB

	---<br>Haben Sie in den letzten 7 Tagen auch andere Medikamente eingenommen?<br>---<br>1 - Ja<br>2 - Nein → **029.20**<br>---<br>(-2) - Keine Antwort → **029.20**		
02940	LINK : 02940_a TO 02940_i : IF 02811={1} OR 02810={2,-2} OR (SEX={2} AND ALTER>=50 AND 02800={1}) OR (SEX={1} AND 02800={1})<br><br>02940_j : IF (SEX={2} AND ALTER>=45 AND ALTER<=49 AND (02811={1}) OR (02810={2,-2})) OR (SEX={2} AND ALTER>=50 AND 02800={1}) OR (SEX={1} AND ALTER>=55 AND 02800={1})<br>---<br>Ich lese Ihnen eine Liste von Medikamenten vor. Bitte sagen Sie mir jedes Mal, wie oft Sie das in den letzten 7 Tagen genommen haben:<br><br>a) Mittel gegen Bluthochdruck<br>b) Herzmedikamente<br>c) Schlafmittel (z.B. Imovane, Sonata)<br>d) Mittel gegen Schmerzen<br>e) Beruhigungsmittel (z.B. Valium, Ativan, Xanax, Temesta)<br>f) Medikamente zur Steigerung von der Aufmerksamkeit oder zum Wachsein (z.B. Ritalin, Medikinet, Concerta, Modasomil)<br>g) Medikamente gegen zu hohes Cholesterin<br>h) Medikamente gegen Depression (z.B. Prozac, Paxil, Effexor)<br>i) Mittel gegen Diabetes oder wegen Diabetes Insulin gespritzt<br>j) Mittel gegen die Osteoporose<br><br>*INT : Items vorlesen!*<br>*Helfen beim Antworten, falls nötig*<br>---<br>1 - Täglich<br>2 - Mehrmals<br>3 - Etwa 1 Mal in den letzten 7 Tagen<br>4 - Nie<br>---<br>(-2) - Keine Antwort	Medikamentenkonsum<br><br><br>TMEKO02<br>TMEKO04<br>TMEKO06<br>TMEKO10<br>TMEKO12<br>TMEKO50<br>TMEKO29<br>TMEKO40<br>TMEKO42<br>TMEKO44	Anal. IGIP tel. 19.00<br>TMEKO50: CoRolAR Core Welle 7, 2014<br>*SGB17: TMEKO50 eingefügt*
02841	LINK : IF 02940_c={1,2,3}<br>---<br>Sind die Schlafmittel vom Arzt verordnet worden?<br>---<br>1 - Ja, vom Arzt verordnet<br>2 - Nein, nicht vom Arzt verordnet	Medikamentenkonsum<br><br><br>TMEKO34	Anal. IGIP schr. 22<br>*SGB92-02: TMEKO07*

	---<br>(-2) - Keine Antwort		
02851	LINK : IF 02940_d={1,2,3}<br>---<br>Sind die Schmerzmittel vom Arzt verordnet worden?<br>---<br>1 - Ja, vom Arzt verordnet<br>2 - Nein, nicht vom Arzt verordnet<br>---<br>(-2) - Keine Antwort	Medikamentenkonsum<br><br>TMEKO35	Anal. IGIP schr. 22<br>*SGB92-02: TMEKO11*
02861	LINK : IF 02940_e={1,2,3}<br>---<br>Sind die Beruhigungsmittel vom Arzt verordnet worden?<br>---<br>1 - Ja, vom Arzt verordnet<br>2 - Nein, nicht vom Arzt verordnet<br>---<br>(-2) - Keine Antwort	Medikamentenkonsum<br><br>TMEKO36	Anal. IGIP schr. 22<br>*SGB92-02: TMEKO13*
02901	LINK : IF 02940_i={1,2,3}<br>---<br>In welcher Form nehmen Sie dieses Mittel wegen Diabetes ein?<br><br>*INT : 2 Antworten möglich*<br>---<br>1 - Tabletten eingenommen<br>2 - Insulin gespritzt<br>3 - Anderes<br>---<br>(-2) - Keine Antwort	Medikamentenkonsum<br><br>TMEKO43a<br>TMEKO43b	BAG (F. Gurtner)
02920<br><br>*PROXY*	LINK : IF SEX=2 AND ALTER>=35 AND NOT PROXY<br><br>Nur an Frauen ab 35 Jahren. Frauen bis 34 Jahre und Männer weiter zu Frage 30.00<br>---<br>Haben Sie gegenwärtig eine Hormonersatzbehandlung?<br><br>*INT : Gemeint ist die Behandlung mit weiblichen Hormonen während oder nach den Wechseljahren, d.h. nach dem Aufhören der Regelblutung (meist zwischen dem 47. und 52. Lebensjahr)*	Medikamentenkonsum<br><br>TMEKO26	BFS - SGB<br>*SGB97:TMEKO48*<br>*le nom de variable a été changé pour l'ESS97 car la population y est différente par rapport aux enquêtes suivantes pour lesquelles la population de référence est restée identique.*

	---<br>1 - Ja<br>2 - Nein<br>---<br>(-1) - Weiss nicht<br>(-2) - Keine Antwort		
03000	LINK : ALL<br>---<br>Haben Sie in den letzten 12 Monaten für sich selber einen SPITEX-Dienst gebraucht, d.h. eine Krankenschwester, eine Haushalts- oder Betagtenhilfe oder den Mahlzeiten- oder Fahrdienst?<br>---<br>1 - Ja<br>2 - Nein → **031.00**<br>---<br>(-1) - Weiss nicht → **031.00**<br>(-2) - Keine Antwort → **031.00**	Inanspruchnahme Spitex<br><br>TINAN24	BFS - SGB
03001	LINK : IF 03000={1}<br>---<br>Ist es vorübergehend oder ist es regelmässig gewesen?<br><br>*INT : Vorübergehend kann auch phasenweise sein*<br>---<br>1 - Vorübergehend<br>2 - Regelmässig<br>---<br>(-1) - Weiss nicht<br>(-2) - Keine Antwort	Inanspruchnahme Spitex<br><br>TINAN30	IUMSP (B. Santos-Eggimann)
03003<br><br>*PROXY*	LINK : IF 03000={1} AND NOT PROXY<br>---<br>Wie häufig haben Sie den Spitexdienst gebraucht?<br><br>*INT : Antwortmöglichkeiten vorlesen!*<br>---<br>1 - Mehrmals pro Woche<br>2 - 1 Mal pro Woche<br>3 - 1 Mal alle 2 Wochen<br>4 - 1 Mal pro Monat<br>5 - Weniger als 1 Mal pro Monat	Inanspruchnahme Spitex<br><br>TINAN60	BFS - SGB

	--<br>(-1) - Weiss nicht<br>(-2) - Keine Antwort		
03002	LINK : IF 03000={1}<br>--<br>Um was für Hilfe, wo Sie in den letzten 12 Monaten bekommen haben, hat es sich gehandelt? Ist das...<br><br>a) Pflege<br>b) Haushalthilfe<br>c) Anderes (z.B. Mahlzeit, Fahrdienst,...)<br><br>*INT : Items vorlesen!*<br>--<br>1 - Ja<br>2 - Nein<br>--<br>(-2) - Keine Antwort	Inanspruchnahme Spitex<br><br><br>TINAN53a<br>TINAN53b<br>TINAN53c	BAG (F. Gurtner)<br>*SGB07-12: TINAN40a-c*
03100	LINK : ALL<br>--<br>Haben Sie für sich selber in den letzten 12 Monaten aus gesundheitlichen Gründen Hilfe von Ihrer Partnerin oder Ihrem Partner, Verwandten, Bekannten oder Nachbarn bekommen, z.B. Hilfe wie Einkaufen, Pflege, Mahlzeiten oder bei administrativen Angelegenheiten?<br><br>*INT : Auch Personen, die mit der Zielperson im selben Haushalt leben*<br>--<br>1 - Ja<br>2 - Nein → **031.10**<br>--<br>(-2) - Keine Antwort → **031.10**	Erhaltene informelle Hilfe<br><br><br>TINHI01	BFS - SGB<br>*SGB92: THIBE01a-b*<br>*SGB97: THIBE09*<br>*SGB02+07: SHIBE01*
03101	LINK : IF 03100={1}<br>--<br>Ist es vorübergehend oder ist es regelmässig gewesen?<br><br>*INT : Vorübergehend kann auch phasenweise sein*<br>--<br>1 - Vorübergehend<br>2 - Regelmässig<br>--<br>(-1) - Weiss nicht<br>(-2) - Keine Antwort	Erhaltene informelle Hilfe<br><br><br>TINHI02	BFS - SGB

03120<br><br>*PROXY*	LINK : IF 03100={1} AND NOT PROXY<br>---<br>Wie häufig haben Sie Hilfe aus gesundheitlichen Gründen von diesen Personen aus Ihrem Umfeld bekommen?<br><br>*INT : Antwortmöglichkeiten vorlesen!*<br>---<br>1 - Mehrmals pro Woche<br>2 - 1 Mal pro Woche<br>3 - 1 Mal alle 2 Wochen<br>4 - 1 Mal pro Monat<br>5 - Weniger als 1 Mal pro Monat<br>---<br>(-1) - Weiss nicht<br>(-2) - Keine Antwort	Erhaltene informelle Hilfe<br><br>TINHI22	BFS - SGB
03121	LINK : 03121_a IF 03100={1} and NBPERS_REG>=2<br>03121_b IF 03100={1}<br>03121_c IF 03100={1}<br>03121_d IF 03100={1}<br>---<br>Von wem alles haben Sie Hilfe bekommen? Handelt es sich um eine oder mehrere Personen…<br><br>a) … in Ihrem Haushalt (if NBPERS_REG>1)<br>b) … aus der Familie, Verwandtschaft, das heisst nicht im gleichen Haushalt<br>c) … Nachbarn, Bekannte, Freunde<br>d) … im Rahmen von Verein, Kirche oder von einer anderen Organisation<br><br>*INT : Items vorlesen!*<br>---<br>1 - Ja<br>2 - Nein<br>---<br>(-2) - Keine Antwort	Erhaltene informelle Hilfe<br><br>TINHI23a<br>TINHI23b<br>TINHI23c<br>TINHI23d	BFS - SGB<br>*SGB92: THIBE02a-e*<br>*SGB97: THIBE11a-e*<br>*SGB02-07: SHIBE03a-j*
03122	LINK : IF 03121_a={1}<br><br>Wenn die Person Hilfe aus ihrem Haushalt erhalten hat (TINHI23A/03121=1)<br>---<br>Was für Hilfe haben Sie von den Personen in Ihrem Haushalt bekommen? Ist das...<br><br>a) Kranken- und Körperpflege<br>b) Hilfe im Haushalt (Mahlzeiten, Einkaufen)<br>c) Andere Hilfe (Transporte, moralische Unterstützung, administrative Angelegenheiten)	Erhaltene informelle Hilfe<br><br>TINHI24a<br>TINHI24b<br>TINHI24c	BFS - SGB<br>*SGB92: THIBE04a-e*<br>*SGB97: THIBE12a-i*<br>*SGB02-07: SHIBE04a-jSGB12: TINHI04a-c*

	INT : Items vorlesen!<br>*"Andere Hilfe" beinhaltet auch z.B. Finanzen verwalten, sich um andere Personen im Haushalt kümmern etc.*<br>---<br>1 - Ja<br>2 - Nein<br>---<br>(-2) - Keine Antwort		
03103	LINK : IF 03121_b={1} OR 03121_c={1} OR 03121_d={1}<br><br>Wenn die Person Hilfe von ausserhalb des Haushalts erhalten hat (TINHI23B-D/03121=1)<br>---<br>Was für Hilfe haben Sie bekommen von den Personen, wo nicht in Ihrem Haushalt wohnen? Ist das...<br><br>a) Kranken- und Körperpflege<br>b) Hilfe im Haushalt (Mahlzeiten, Einkaufen)<br>c) Andere Hilfe (Transporte, moralische Unterstützung, administrative Angelegenheiten)<br><br>*INT : Items vorlesen!*<br>*"Andere Hilfe" beinhaltet auch z.B. Finanzen verwalten, sich um andere Personen im Haushalt kümmern etc.*<br>---<br>1 - Ja<br>2 - Nein<br>---<br>(-2) - Keine Antwort	Erhaltene informelle Hilfe<br><br><br><br>TINHI25a<br>TINHI25b<br>TINHI25c	BFS - SGB<br>*SGB92: THIBE04a-e*<br>*SGB97: THIBE12a-i*<br>*SGB02-07: SHIBE04a-j*<br>*SGB12: TINHI04a-c*
03110<br><br><br>*PROXY*	LINK : IF (03000={2,-1,-2} AND 03100={2,-2}) AND (01800_a-e={2,3,4} OR 01810_a-h={2,3,4} OR 01820={2,3,4} OR 01200={1,2} OR 01310={2,3,4} OR 01410={2,3,4} OR 01500={2,3,4} OR 01600={2,3,4} OR 01650={2,3,4} OR 01651={2,3,4}) AND NOT PROXY<br><br>Nur Personen, die bei der Spitex (TINAN24/03000=2, -1, -2), ET "NON" (+NSP + NR) der informellen Hilfe mit „Nein" (+WN+KA) geantwortet haben (TINHI01/03100=2, -1, -2) UND die entweder mindestens eine ADL haben (TBADL02a-e/01800= 2, 3, 4 oder TIADL02a-h/01810=2,3,4 oder TIADL02HH/01820=2, 3, 4) oder die Schwierigkeiten oder Probleme bei Behinderungen haben (TKRAN08/01200=1, 2 oder TSBHD03/01310=2, 3, 4 oder THBHD02/01410=2, 3, 4 oder TRBHD03/01500=2, 3, 4 oder TBBHD03/01600=2,3 ,4 oderTKBHD01/01650=2, 3, 4 oder TKBHD02/01651=2, 3, 4)<br>Die anderen weiter zu Frage 03200<br>---<br>Würden Sie aufgrund von gesundheitlichen Problemen Hilfe von Personen aus Ihrem Umfeld oder von der Spitex brauchen?<br>---<br>1 - Ja<br>2 - Nein	Erhaltene informelle Hilfe<br><br><br><br>TINHI26	BFS - SGB<br>*SGB92: THIBE05*<br>*SGB12: TINHI16*

	--<br>(-1) - Weiss nicht<br>(-2) - Keine Antwort		
03200	LINK : ALL<br><br>Wieder an alle<br>--<br>Haben Sie in den letzten 12 Monaten einer oder mehreren Personen mit gesundheitlichen Problemen geholfen? Das kann innerhalb oder ausserhalb von Ihrem Haushalt sein. Zum Beispiel im Haushalt helfen, Essen bringen oder Transporte machen von Kranken, Behinderten oder älteren Personen.<br>--<br>1 - Ja<br>2 - Nein **→ 033.00**<br>--<br>(-2) - Keine Antwort **→ 033.00**	Geleistete informelle Hilfe<br><br><br>TINHI27	anal. UNI-LONDON 65<br>*SGB92 : TSOUN01*<br>*SGB97 : TSOUN09-10*<br>*SGB02+07: SSOUN04*<br>*SGB12: TINHI17*<br>*SGB17: Frage angepasst*
03205	LINK : IF 03200={1}<br>--<br>Ist es vorübergehend oder ist es regelmässig gewesen?<br><br>*INT : Vorübergehend kann auch phasenweise sein*<br>--<br>1 - Vorübergehend<br>2 - Regelmässig<br>--<br>(-1) - Weiss nicht<br>(-2) - Keine Antwort	Geleistete informelle Hilfe<br><br><br>TINHI28	BFS - SGB
03201<br><br><br>*PROXY*	LINK : IF 03200={1} AND NOT PROXY<br>--<br>Wie häufig haben Sie einer oder mehreren Personen mit Gesundheitsproblemen geholfen?<br><br>*INT : Antwortmöglichkeiten vorlesen!*<br>--<br>1 - Mehrmals pro Woche<br>2 - 1 Mal pro Woche<br>3 - 1 Mal alle 2 Wochen<br>4 - 1 Mal pro Monat<br>5 - Weniger als 1 Mal im Monat<br>--<br>(-1) - Weiss nicht<br>(-2) - Keine Antwort	Geleistete informelle Hilfe<br><br><br>TINHI29	anal. UNI-LONDON 65<br>*SGB92 : TSOUN01*<br>*SGB97 : TSOUN09-10*<br>*SGB02+07: SSOUN05*<br>*SGB12: TINHI18*<br>*SGB17: Enoncé et catégories adaptés*

03202	LINK : 03202_a IF 03200={1} AND NBPERS_REG>1<br>03202_b IF 03200={1}<br>03202_c IF 03200={1}<br>03202_d IF 03200={1}<br>---<br>Wem haben Sie geholfen? Handelt es sich um eine oder mehrere Personen…<br><br>a) … in Ihrem Haushalt (if NBPERS_REG>1)<br>b) … aus der Familie, Verwandtschaft, das heisst nicht im gleichen Haushalt<br>c) … Nachbarn, Bekannte, Freunde<br>d) … im Rahmen von Verein, Kirche oder von einer anderen Organisation<br><br>*INT : Items vorlesen!*<br>---<br>1 - Ja<br>2 - Nein<br>---<br>(-2) - Keine Antwort	Geleistete informelle Hilfe<br><br><br>TINHI30a<br>TINHI30b<br>TINHI30c<br>TINHI30d	BFS - SGB<br>*SGB12: TINHI19a-d*
03203	LINK : IF 03202_a={1}<br><br>Falls die Person jemandem im eigenen Haushalt hilft (TINHI30a/32.02 = 1)<br>---<br>Was für Hilfe haben Sie für die Personen in Ihrem Haushalt geleistet? Ist das…<br><br>a) Kranken- und Körperpflege<br>b) Hilfe im Haushalt (Mahlzeiten, Einkaufen)<br>c) Andere Hilfe (Transporte, moralische Unterstützung, administrative Angelegenheiten)<br><br>*INT : Items vorlesen!*<br>*"Andere Hilfe" beinhaltet auch z.B. Finanzen verwalten, sich um andere Personen im Haushalt kümmern etc.*<br>---<br>1 - Ja<br>2 - Nein<br>---<br>(-2) - Keine Antwort	Geleistete informelle Hilfe<br><br><br>TINHI31a<br>TINHI31b<br>TINHI31c	BFS - SGB<br>*SGB12: TINHI20a-c*
03204	LINK : IF 03202_b={1} OR 03202_c={1} OR 03202_d={1}<br><br>Falls die Person jemandem ausserhalb seines Haushalts hilft (TINHI30b-d/32.02 = 1)<br>---<br>Was für Hilfe haben Sie für die Personen geleistet, wo nicht in Ihrem Haushalt leben?	Geleistete informelle Hilfe<br><br><br>TINHI32a	BFS - SGB<br>*SGB12: TINHI21a-c*

	a) Kranken- und Körperpflege<br>b) Hilfe im Haushalt (Mahlzeiten, Einkaufen)<br>c) Andere Hilfe (Transporte, moralische Unterstützung, administrative Angelegenheiten)<br><br>*INT : Items vorlesen!*<br>*"Andere Hilfe" beinhaltet auch z.B. Finanzen verwalten, sich um andere Personen im Haushalt kümmern etc.*<br>---<br>1 - Ja<br>2 - Nein<br>---<br>(-2) - Keine Antwort	TINHI32b<br>TINHI32c	
03300<br><br>*PROXY*	LINK : ALL AND NOT PROXY<br><br>WIEDER AN ALLE<br>---<br>Jetzt möchte ich gerne wissen, wie Sie Ihre Lebensqualität im Allgemeinen einschätzen?<br><br>*INT : Antwortmöglichkeiten vorlesen!*<br>---<br>1 - Sehr gut<br>2 - Gut<br>3 - Weder gut noch schlecht<br>4 - Schlecht<br>5 - Sehr schlecht<br>---<br>(-2) - Keine Antwort	Soziale Unterstützung<br>Lebensqualität<br><br>TSOUN24	BFS - SGB
03310<br><br>*PROXY*	LINK : ALL AND NOT PROXY<br>---<br>Wie viele Menschen stehen Ihnen so nahe, dass Sie sich auf sie verlassen können, wenn Sie ein ernstes persönliches Problem haben?<br><br>*INT : Beim Schätzen helfen!*<br>---<br>1 - 0<br>2 - 1-2<br>3 - 3-5<br>4 - 6 oder mehr<br>---<br>(-1) - Weiss nicht<br>(-2) - Keine Antwort	Soziale Unterstützung<br><br>TSOUN25	Oslo-Scale, EHIS
03311	LINK : ALL AND NOT PROXY	Soziale Unterstützung	Oslo-Scale, EHIS

PROXY	---<br>Wie viel Interesse und Anteilnahme zeigen andere Menschen an dem, was Sie machen?<br><br>*INT : Antwortmöglichkeiten vorlesen!*<br>---<br>1 - Viel<br>2 - Ziemlich viel<br>3 - Weder viel noch wenig<br>4 - Wenig<br>5 - Kein Interesse<br>---<br>(-1) - Weiss nicht<br>(-2) - Keine Antwort	TSOUN29	*SGB12: TSOUN26*<br>*SGB17: Fragestellung in F und I modifiziert sowie Antwortkategorien in D und I angepasst und vereinheitlicht*
03312<br><br>*PROXY*	LINK : ALL AND NOT PROXY<br>---<br>Wie einfach wäre es für Sie, Hilfe von Nachbarn zu bekommen, wenn Sie diese benötigen?<br><br>*INT : Antwortmöglichkeiten vorlesen!*<br>---<br>1 - Sehr leicht<br>2 - Leicht<br>3 - Möglich<br>4 - Schwierig<br>5 - Sehr schwierig<br>---<br>(-1) - Weiss nicht<br>(-2) - Keine Antwort	Soziale Unterstützung<br><br>TSOUN27	Oslo-Scale, EHIS
03320<br><br>*PROXY*	LINK : ALL AND NOT PROXY<br>---<br>Gibt es unter den Personen, wo Ihnen nahestehen, öpper (jemanden), wo Sie wirklich jederzeit über ganz persönliche Probleme reden können?<br><br>*INT : Diese Person kann auch der Partner sein! Es können Verwandte oder Familienmitglieder, Freunde/innen, Nachbarn, Arbeitskollegen/innen usw. sein.*<br>---<br>1 - Ja, mehrere Personen<br>2 - Ja, eine Person<br>3 - Nein	Soziale Unterstützung<br><br>TSOUN05	IGIP tél. 46.00

	---<br>(-1) - Weiss nicht<br>(-2) - Keine Antwort		
03330<br><br>*PROXY*	LINK : ALL AND NOT PROXY<br>---<br>Vermissen Sie manchmal eine Person, wo Sie wirklich jederzeit über ganz persönliche Probleme reden können?<br>---<br>1 - Ja<br>2 - Nein<br>---<br>(-1) - Weiss nicht<br>(-2) - Keine Antwort	Soziale Unterstützung<br><br>TSOUN30	IGIP tél 47.00<br>*Ab SGB17 unterschiedliche Referenzpopulation*<br>*SGB17: Frage angepasst*<br>*SGB92-02: TSOUN06*<br>*SGB07-12: TSOUN18*
03340	LINK : ALL<br>---<br>Wie häufig nehmen Sie an einem Anlass teil von einem Verein, Klub, einer politischen Partei, einer kulturellen Vereinigung oder anderen Gruppen, darunter auch religiöse?<br><br>*INT : z.B. Fussball-Training, Elternverein-Anlässe, Lesezirkel, Gottesdienstbesuch, usw.*<br>---<br>1 - Fast täglich<br>2 - Etwa 1 Mal pro Woche<br>3 - Etwa 1-3 Mal pro Monat<br>4 - Ein paar Mal pro Jahr<br>5 - Seltener<br>6 - Nie<br>---<br>(-1) - Weiss nicht<br>(-2) - Keine Antwort	Soziale Unterstützung<br>Soziale Teilnahme<br><br>TSOUN28	UNI-LONDON 66b) angepasst<br>*(in Anlehnung an SHP)*
03400<br><br>*PROXY*	LINK : ALL AND NOT PROXY<br>---<br>Wie häufig kommt es vor, dass Sie sich einsam fühlen? Kommt das… vor?<br><br>*INT : Antwortmöglichkeiten vorlesen!*<br>---<br>1 - Sehr häufig<br>2 - Ziemlich häufig<br>3 - Manchmal<br>4 - Nie<br>---<br>(-2) - Keine Antwort	Psychische Gesundheit<br><br>TPSYG05	BFS - SGB

03600<br><br>*PROXY*	LINK : ALL AND NOT PROXY<br>--<br>Wir kommen jetzt zu Fragen, wo das Verhalten im Alltag betreffen. Kommen Sie in Ihrer Freizeit mindestens einmal pro Woche durch körperliche Betätigung zum Schwitzen? z.B. durch Rennen, Velofahren, Sport treiben usw.<br>--<br>1 - Ja<br>2 - Nein → **036.10**<br>--<br>(-2) - Keine Antwort → **036.10**	Körperliche Aktivität<br><br>TKOBW01	USA
03601<br><br>*PROXY*	LINK : IF 03600={1} AND NOT PROXY<br>--<br>An wie vielen Tagen pro Woche im Durchschnitt?<br><br><x> - Tagen pro Woche<br>--<br>(-2) - Keine Antwort	Körperliche Aktivität<br><br>TKOBW02	USA
03610<br><br>*PROXY*	LINK : ALL AND NOT PROXY<br>--<br>Jetzt geht es um weniger intensive Bewegungsformen. Also um Aktivitäten, wo Sie zumindest ein bisschen ausser Atem kommen, aber nicht unbedingt ins Schwitzen. Das sind zum Beispiel zügiges Laufen, Wandern, Tanzen, Gartenarbeiten:<br>An wievielen Tagen pro Woche machen Sie solche körperliche Aktivitäten?<br><br><x> - Tagen pro Woche<br>--<br>0 - Nie → **036.50**<br>--<br>(-1) - Weiss nicht → **036.50**<br>(-2) - Keine Antwort → **036.50**	Körperliche Aktivität<br><br>TKOBW09	SWI-BASPO 2000
03611<br><br>*PROXY*	LINK : IF 03610>={1} AND NOT PROXY<br>--<br>Wie lange sind Sie an jedem von diesen Tagen aktiv, im Durchschnitt?<br><br><xx> Stunden pro Tag (TKOBW11a)<br><xx> Minuten pro Tag (TKOBW11b)<br><br>*INT : „Aktiv" bezieht sich nur auf weniger intensive Bewegungsformen*<br>--<br>(-1) - Weiss nicht<br>(-2) - Keine Antwort	Körperliche Aktivität<br><br>TKOBW11<br>[TKOBW11a]<br>[TKOBW11b]	Anal. SWI-BASPO 2000<br>*Variable im Datensatz ist in Minuten*
03650	LINK : ALL AND NOT PROXY	Körperliche Aktivität	Item aus dem IPAQ

PROXY	---<br>Jetzt geht es ums Sitzen, zum Beispiel bei der Arbeit, zu Hause, auf dem Weg von einem Ort zum andern oder während der Freizeit, am Tisch, vor dem Fernseher oder beim Lesen. Alles in allem, wieviele Stunden verbringen Sie insgesamt an einem gewöhnlichen Wochentag im Sitzen?<br><br><xx> Stunden pro Tag (TKOBW17)<br><br>*INT : Geben Sie bitte einen Durchschnitt pro Tag an.*<br>*Ab 30 Minuten auf 1 Stunde aufrunden.*<br>*Nur Montag bis Freitag*<br>---<br>(-1) - Weiss nicht<br>(-2) - Keine Antwort	TKOBW17	*Antworten nur in Stunden (und nicht zusätzlich in Minuten)*<br>*SGB12: SKOBW09*
03651<br><br>*PROXY*	LINK : IF 03650 >={1} AND NOT PROXY<br>---<br>Wie häufig unterbrechen Sie das Sitzen, also wie häufig stehen Sie auf?<br><br>*INT : Antwortmöglichkeiten vorlesen!*<br>---<br>1 - Weniger als 1 Mal die Stunde<br>2 - Stündlich<br>3 - Halbstündlich<br>4 - Alle 15 Minuten<br>5 - Alle 10 Minuten<br>6 - Alle 5 Minuten<br>---<br>(-1) - Weiss nicht<br>(-2) - Keine Antwort	Körperliche Aktivität<br><br>TKOBW18	BFS - SGB (zusammen mit BASPO und BAG)
03700	LINK : ALL<br>---<br>Wir möchten Ihnen jetzt ein paar Fragen zu Ihrer Ernährung stellen. Achten Sie auf bestimmte Sachen bei Ihrer Ernährung?<br>---<br>1 - Ja, achte auf etwas<br>2 - Nein, achte auf nichts<br>---<br>(-2) - Keine Antwort	Ernährung<br><br>TERNA01	IGIP tél. 14.00
03710	LINK : ALL<br>---<br>Haben Sie in den letzten 12 Monaten eine spezielle Diät eingehalten?	Ernährung<br><br>TERNA14	ISPM BE (M. Zwahlen)

	--<br>1 - Ja<br>2 - Nein → **037.20**<br>--<br>(-1) - Weiss nicht → **037.20**<br>(-2) - Keine Antwort → **037.20**		
03711	LINK : IF 03710={1}<br>--<br>Warum haben Sie eine spezielle Diät eingehalten?<br><br>*INT : Helfen beim Antworten, falls nötig*<br>--<br>1 - Um Gewicht zu verlieren, ohne medizinische Gründe<br>2 - Aus medizinischen Gründen<br>3 - Aus anderen Gründen (weder medizinisch, noch um Gewicht zu verlieren)<br>--<br>(-1) - Weiss nicht<br>(-2) - Keine Antwort	Ernährung<br><br><br>TERNA15	ISPM BE (M. Zwahlen)
03720	LINK : ALL<br>--<br>An wie vielen Tagen pro Woche essen Sie gewöhnlich Gemüse oder Salat oder trinken Sie Gemüsesäfte? Kartoffeln zählen nicht dazu.<br>--<br>0 - Nie → **037.30**<br>8 - Weniger als 1 Tag pro Woche → **037.30**<br>1 - 1 Tag pro Woche → **037.30**<br>2 - 2 Tage pro Woche → **037.30**<br>3 - 3 Tage pro Woche → **037.30**<br>4 - 4 Tage pro Woche → **037.30**<br>5 - 5 Tage pro Woche<br>6 - 6 Tage pro Woche<br>7 - 7 Tage pro Woche<br>--<br>(-2) - Keine Antwort → **037.30**	Ernährung<br><br><br>TERNA20	Anal. SALUTE C3.3<br>*SGB92-02: TERNA06*
03721<br><br><br>*PROXY*	LINK : IF 03720={5,6,7} AND NOT PROXY<br>--<br>Und wie viele Portionen Gemüse, Salat oder Gemüsesäfte nehmen Sie im Durchschnitt am Tag zu sich? Eine Portion wäre etwa so gross wie Ihre Hand voll oder etwa 1 Tomate, 1 grosses Rüebli. Beim Saft sind es etwa 2dl.<br>--<br>0 - Weniger als 1 Portion<br>1 - 1 Portion	Ernährung<br><br><br>TERNA21	ISPM BE (M. Zwahlen)<br>*SGB07 : TERNA17*

	2 - 2 Portionen<br>3 - 3 Portionen<br>4 - 4 Portionen<br>5 - 5 Portionen und mehr<br>---<br>(-1) - Weiss nicht<br>(-2) - Keine Antwort		
03730	LINK : ALL<br>---<br>An wie vielen Tagen pro Woche essen Sie im Durchschnitt Früchte oder trinken Sie Fruchtsäfte?<br>---<br>0 - Nie → **037.40**<br>8 - Weniger als 1 Tag pro Woche → **037.40**<br>1 - 1 Tag pro Woche → **037.40**<br>2 - 2 Tage pro Woche → **037.40**<br>3 - 3 Tage pro Woche → **037.40**<br>4 - 4 Tage pro Woche → **037.40**<br>5 - 5 Tage pro Woche<br>6 - 6 Tage pro Woche<br>7 - 7 Tage pro Woche<br>---<br>(-2) - Keine Antwort → **037.40**	Ernährung<br><br>TERNA22	Anal. SALUTE C3.3<br>*SGB92-97: TERNA07 (ohne Saft)*<br>*SGB02: TERNA07 + TERNA10*
03731<br><br>*PROXY*	LINK : IF 03730={5,6,7} AND NOT PROXY<br>---<br>Und wie viele Portionen Früchte oder Fruchtsäfte nehmen Sie im Durchschnitt am Tag zu sich? Eine Portion wäre etwa so gross wie Ihre Hand voll also 1 Apfel, 1 Birne. Beim Saft sind es etwa 2dl.<br>---<br>0 - Weniger als 1 Portion<br>1 - 1 Portion<br>2 - 2 Portionen<br>3 - 3 Portionen<br>4 - 4 Portionen<br>5 - 5 Portionen und mehr<br>---<br>(-1) - Weiss nicht<br>(-2) - Keine Antwort	Ernährung<br><br>TERNA23	ISPM BE (M. Zwahlen)<br>*SGB07: TERNA16*
03740	LINK : ALL<br>---<br>An wie vielen Tagen pro Woche essen Sie im Allgemeinen Fleisch oder Wurstwaren?	Ernährung<br><br>TERNA03	SALUTE C 3.2

	---<br>0 - Nie<br>8 - Weniger als 1 Tag pro Woche<br>1 - 1 Tag pro Woche<br>2 - 2 Tage pro Woche<br>3 - 3 Tage pro Woche<br>4 - 4 Tage pro Woche<br>5 - 5 Tage pro Woche<br>6 - 6 Tage pro Woche<br>7 - 7 Tage pro Woche<br>---<br>(-2) - Keine Antwort		
03750	LINK : ALL<br>---<br>An wie vielen Tagen pro Woche essen Sie Fisch, im Durchschnitt?<br>---<br>0 - Nie<br>8 - Weniger als 1 Tag pro Woche<br>1 - 1 Tag pro Woche<br>2 - 2 Tage pro Woche<br>3 - 3 Tage pro Woche<br>4 - 4 Tage pro Woche<br>5 - 5 Tage pro Woche<br>6 - 6 Tage pro Woche<br>7 - 7 Tage pro Woche<br>---<br>(-2) - Keine Antwort	Ernährung<br><br>TERNA05	SALUTE C 3.3
03780	LINK : ALL<br>---<br>An wie vielen Tagen pro Woche essen Sie im Durchschnitt Süssigkeiten oder Desserts?<br><br>*INT : z. B. Schokolade, Guetzli, Glace, Kuchen, Schoggiweggli*<br>---<br>0 - Nie<br>8 - Weniger als 1 Tag pro Woche<br>1 - 1 Tag pro Woche<br>2 - 2 Tage pro Woche<br>3 - 3 Tage pro Woche<br>4 - 4 Tage pro Woche<br>5 - 5 Tage pro Woche	Ernährung<br><br>TERNA25	BFS - SGB;<br>basierend auf SGE “nutricalc” und STPH “SAPALDIA 4”

	6 - 6 Tage pro Woche<br>7 - 7 Tage pro Woche<br>---<br>(-2) - Keine Antwort		
03781<br><br>*PROXY*	LINK : IF 03780={5,6,7} AND NOT PROXY<br>---<br>Und wie viele Portionen Süssigkeiten oder Desserts nehmen Sie im Durchschnitt am Tag zu sich?<br>Eine Portion entspricht etwa 1 Reihe Schokolade, 3 Stück Guetzli, 1 Kugel Glace, 1 (kleines) Stück Kuchen, 1 Schoggiweggli.<br>---<br>0 - Weniger als 1 Portion<br>1 - 1 Portion<br>2 - 2 Portionen<br>3 - 3 Portionen<br>4 - 4 Portionen<br>5 - 5 Portionen und mehr<br>---<br>(-1) - Weiss nicht<br>(-2) - Keine Antwort	Ernährung<br><br>TERNA26	BFS - SGB
03782	LINK : ALL<br>---<br>An wie vielen Tagen pro Woche essen Sie im Durchschnitt salzige Knabbereien?<br><br>*INT : z. B. Pommes Chips, Salzstängeli, Blätterteiggebäck, gesalzener Nüsse*<br>---<br>0 - Nie<br>8 - Weniger als 1 Tag pro Woche<br>1 - 1 Tag pro Woche<br>2 - 2 Tage pro Woche<br>3 - 3 Tage pro Woche<br>4 - 4 Tage pro Woche<br>5 - 5 Tage pro Woche<br>6 - 6 Tage pro Woche<br>7 - 7 Tage pro Woche<br>---<br>(-2) - Keine Antwort	Ernährung<br><br>TERNA27	BFS - SGB;<br>basierend auf SGE “nutricalc” und STPH “SAPALDIA 4”
03783<br><br>*PROXY*	LINK : IF 03782={5,6,7} AND NOT PROXY<br>---<br>Und wie viele Portionen salzige Knabbereien nehmen Sie im Durchschnitt am Tag zu sich?<br>Eine Portion entspricht etwa 1 Hand voll Pommes Chips, Salzstängeli, Blätterteiggebäck, gesalzener Nüsse.	Ernährung<br><br>TERNA28	BFS - SGB

	---<br>0 - Weniger als 1 Portion<br>1 - 1 Portion<br>2 - 2 Portionen<br>3 - 3 Portionen<br>4 - 4 Portionen<br>5 - 5 Portionen und mehr<br>---<br>(-1) - Weiss nicht<br>(-2) - Keine Antwort		
03790	LINK : ALL<br>---<br>An wie vielen Tagen pro Woche trinken Sie im Durchschnitt gezuckerte Getränke wie Limonaden, Eistee, Sirup, Energy Drinks, Cola?<br>Achtung: Berücksichtigen Sie hier keine « light»-Getränke.<br><br>*INT : z.B. Pepsi, Sprite, 7-Up, Fanta, Rivella rot, Sinalco, Red Bull, Monster*<br>---<br>0 - Nie<br>8 - Weniger als 1 Tag pro Woche<br>1 - 1 Tag pro Woche<br>2 - 2 Tage pro Woche<br>3 - 3 Tage pro Woche<br>4 - 4 Tage pro Woche<br>5 - 5 Tage pro Woche<br>6 - 6 Tage pro Woche<br>7 - 7 Tage pro Woche<br>---<br>(-2) - Keine Antwort	Ernährung<br><br><br>TERNA29	BFS - SGB;<br>basierend auf SGE "nutricalc" und STPH "SAPALDIA 4"
03791<br><br><br>*PROXY*	LINK : IF 03790={5,6,7} AND NOT PROXY<br>---<br>Und wie viel gezuckerte Getränke trinken Sie im Durchschnitt am Tag?<br><br>*INT : Helfen beim Antworten, falls nötig*<br>*z.B. Pepsi, Sprite, 7-Up, Fanta, Rivella rot, Sinalco, Red Bull, Monster*<br>---<br>0 - Weniger als 1 Glas<br>1 - 1 Glas (2,5 dl)<br>2 - 2 Gläser (5 dl)<br>3 - 3 Gläser (7,5 dl)<br>4 - 4 Gläser (10 dl)<br>5 - 5 Gläser oder mehr (mehr als 1 Liter)	Ernährung<br><br><br>TERNA30	BFS - SGB

	---<br>(-1) - Weiss nicht<br>(-2) - Keine Antwort		
03800	LINK : ALL<br>---<br>Wie häufig trinken Sie normalerweise alkoholische Getränke, also Bier, Wein, Likör, Aperitif, Spirituosen, Schnaps?<br><br>*INT : Helfen beim Antworten, falls nötig*<br>---<br>1 - 3 Mal oder mehr pro Tag → **038.20**<br>2 - 2 Mal pro Tag (zu Mahlzeiten) → **038.20**<br>3 - 1 Mal pro Tag → **038.20**<br>4 - Mehrmals pro Woche → **038.20**<br>5 - 1-2 Mal pro Woche → **038.20**<br>6 - 1-3 Mal pro Monat → **038.20**<br>7 - Weniger als 1 Mal pro Monat → **038.61**<br>8 - Nie, abstinent<br>---<br>(-2) - Keine Antwort	Alkoholkonsum<br><br><br>TALKO15	Anal. IGIP tél. 26.00<br>*SGB92-02: TALKO01*
03810	LINK : IF 03800={8,-2}<br>---<br>Haben Sie früher einmal Alkohol getrunken?<br>---<br>1 - Ja → **039.00**<br>2 - Nein → **039.00**<br>---<br>(-2) - Keine Antwort → **039.00**	Alkoholkonsum<br><br><br>TALKO19	BFS - SGB
03820	LINK : IF 03800={1 TO 6}<br>---<br>Haben Sie in den letzten 7 Tagen Bier getrunken?<br><br>*INT : Ohne alkoholfreies Bier!*<br>---<br>1 - Ja<br>2 - Nein → **038.23**<br>---<br>(-2) - Keine Antwort → **038.23**	Alkoholkonsum<br><br><br>TALKO02a	TREND 44<br>*SGB92: SALKO02a*
03821	LINK : IF 03820={1} AND NOT PROXY	Alkoholkonsum	TREND 44<br>*SGB92: SALKO02b*

PROXY	---<br>Wie häufig?<br><br>*INT : Helfen beim Antworten, falls nötig*<br>---<br>1 - 3 Mal täglich oder mehr<br>2 - 2 Mal täglich<br>3 - 1 Mal täglich<br>4 - Fast täglich<br>5 - 3 oder 4 Mal in den letzten 7 Tagen<br>6 - 1 oder 2 Mal in den letzten 7 Tagen<br>---<br>(-2) - Keine Antwort	TALKO02b	
03822<br><br>*PROXY*	LINK : IF 03820={1} AND NOT PROXY<br>---<br>Und wie viel pro Mal?<br><br>*INT : Helfen beim Antworten, falls nötig*<br>---<br>1 - 5 Gläser / kleine Flaschen à 3 dl oder 3 Flaschen/Dosen à 5 dl oder mehr<br>2 - 3-4 Gläser / kleine Flaschen à 3 dl oder 2 Flaschen/Dosen à 5 dl<br>3 - 2 Gläser / kleine Flaschen à 3 dl oder 1 Flasche/Dose à 5 dl<br>4 - 1 Glas / kleine Flasche à 3 dl<br>---<br>(-2) - Keine Antwort	Alkoholkonsum<br><br>TALKO02c	TREND 44<br>*SGB97 Mengenangaben leicht modifiziert*<br>*SGB92: SALKO02c*
03823	LINK : IF 03820={2,-2}<br><br>Nur Personen, die in den letzten 7 Tagen kein Bier (+KA) getrunken haben (TALKO02a/38.20=2, -2). Die Anderen weiter zur Frage 38.30<br>---<br>Haben Sie in den letzten 12 Monaten Bier getrunken?<br><br>*INT : Ohne alkoholfreies Bier!*<br>---<br>1 - Ja<br>2 - Nein **→ 038.30**<br>---<br>(-2) - Keine Antwort **→ 038.30**	Alkoholkonsum<br><br>TALKO02d	TREND 44<br>*SGB92: SALKO02d*
03824	LINK : IF 03823={1} AND NOT PROXY<br>---<br>Wie häufig?	Alkoholkonsum	TREND 44<br>*SGB92: SALKO02e*

PROXY	*INT : Helfen beim Antworten, falls nötig*<br>---<br>1 - Jede Woche<br>2 - 2-3 Mal pro Monat<br>3 - Ca. 1 Mal pro Monat<br>4 - Weniger als 1 Mal pro Monat<br>---<br>(-2) - Keine Antwort	TALKO02e	
03825<br><br>*PROXY*	LINK : IF 03823={1} AND NOT PROXY<br>---<br>Und wie viel pro Mal?<br><br>*INT : Helfen beim Antworten, falls nötig*<br>---<br>1 - 5 Gläser / kleine Flaschen à 3 dl oder 3 Flaschen/Dosen à 5 dl oder mehr<br>2 - 3-4 Gläser / kleine Flaschen à 3 dl oder 2 Flaschen/Dosen à 5 dl<br>3 - 2 Gläser / kleine Flaschen à 3 dl oder 1 Flasche/Dose à 5 dl<br>4 - 1 Glas / kleine Flasche à 3 dl<br>---<br>(-2) - Keine Antwort	Alkoholkonsum<br><br>TALKO02f	TREND 44<br>*SGB97 Mengenangaben leicht modifiziert*<br>*SGB92: SALKO02f*
03830	LINK : IF 03800={1 TO 6}<br>---<br>Haben Sie in den letzten 7 Tagen Wein getrunken?<br>---<br>1 - Ja<br>2 - Nein **→ 038.33**<br>---<br>(-2) - Keine Antwort **→ 038.33**	Alkoholkonsum<br><br>TALKO03a	TREND 44<br>*SGB92: SALKO03a*
03831<br><br>*PROXY*	LINK : IF 03830={1} AND NOT PROXY<br>---<br>Wie häufig?<br><br>*INT : Helfen beim Antworten, falls nötig*<br>---<br>1 - 3 Mal täglich oder mehr<br>2 - 2 Mal täglich<br>3 - 1 Mal täglich<br>4 - Fast täglich	Alkoholkonsum<br><br>TALKO03b	TREND 44<br>*SGB92: SALKO03b*

	5 - 3 oder 4 Mal in den letzten 7 Tagen<br>6 - 1 oder 2 Mal in den letzten 7 Tagen<br>--<br>(-2) - Keine Antwort		
03832<br><br>*PROXY*	LINK : IF 03830={1} AND NOT PROXY<br>--<br>Und wie viel pro Mal?<br><br>*INT : Helfen beim Antworten, falls nötig*<br>--<br>1 - 1/2 Liter oder mehr (5 Gläser à 1 dl oder mehr)<br>2 - 3-4 dl (3-4 Gläser)<br>3 - 2 dl (2 Gläser)<br>4 - 1 dl (1 Glas)<br>--<br>(-2) - Keine Antwort	Alkoholkonsum<br><br>TALKO03c	TREND 44<br>*SGB97 Mengenangaben leicht modifiziert*<br>*SGB92: SALKO03c*
03833	LINK : IF 03830={2,-2}<br><br>Nur Personen, die in den letzten 7 Tagen keinen Wein (+KA) getrunken haben (TALKO03a/38.30=2, -2). Die Anderen weiter zur Frage 38.40<br>--<br>Haben Sie in den letzten 12 Monaten Wein getrunken?<br>--<br>1 - Ja<br>2 - Nein → **038.40**<br>--<br>(-2) - Keine Antwort → **038.40**	Alkoholkonsum<br><br>TALKO03d	TREND 44<br>*SGB92: SALKO03d*
03834<br><br>*PROXY*	LINK : IF 03833={1} AND NOT PROXY<br>--<br>Wie häufig?<br><br>*INT : Helfen beim Antworten, falls nötig*<br>--<br>1 - Jede Woche<br>2 - 2-3 Mal pro Monat<br>3 - Ca. 1 Mal pro Monat<br>4 - Weniger als 1 Mal pro Monat<br>--<br>(-2) - Keine Antwort	Alkoholkonsum<br><br>TALKO03e	TREND 44<br>*SGB92: SALKO03e*
03835	LINK : IF 03833={1} AND NOT PROXY	Alkoholkonsum	TREND 44

PROXY	--<br>Und wie viel pro Mal?<br><br>*INT : Helfen beim Antworten, falls nötig*<br>--<br>1 - 1/2 Liter oder mehr (5 Gläser à 1 dl oder mehr)<br>2 - 3-4 dl (3-4 Gläser)<br>3 - 2 dl (2 Gläser)<br>4 - 1 dl (1 Glas)<br>--<br>(-2) - Keine Antwort	TALKO03f	*SGB97 Mengenangaben leicht modifiziert*<br>*SGB92: SALKO03f*
03840	LINK : IF 03800={1 TO 6}<br>--<br>Haben Sie in den letzten 7 Tagen Spirituosen, das heisst Schnaps, Whisky, Cognac, Likör usw. getrunken?<br>--<br>1 - Ja<br>2 - Nein → **038.43**<br>--<br>(-2) - Keine Antwort → **038.43**	Alkoholkonsum<br><br>TALKO05a	TREND 44<br>*SGB92: SALKO05a*
03841<br><br>*PROXY*	LINK : IF 03840={1} AND NOT PROXY<br>--<br>Wie häufig?<br><br>*INT : Helfen beim Antworten, falls nötig*<br>--<br>1 - 3 Mal täglich oder mehr<br>2 - 2 Mal täglich<br>3 - 1 Mal täglich<br>4 - Fast täglich<br>5 - 3 oder 4 Mal in den letzten 7 Tagen<br>6 - 1 oder 2 Mal in den letzten 7 Tagen<br>--<br>(-2) - Keine Antwort	Alkoholkonsum<br><br>TALKO05b	TREND 44<br>*SGB92: SALKO05b*
03842<br><br>*PROXY*	LINK : IF 03840={1} AND NOT PROXY<br>--<br>Und wieviel pro Mal?<br><br>*INT : Helfen beim Antworten, falls nötig*	Alkoholkonsum<br><br>TALKO05c	TREND 44<br>*SGB97 Mengenangaben leicht modifiziert*<br>*SGB92: SALKO05c*

	---<br>1 - 5-6 Gläschen oder mehr<br>2 - 3-4 Gläschen<br>3 - 2 Gläschen<br>4 - 1 Gläschen<br>---<br>(-2) - Keine Antwort		
03843	LINK : IF 03840={2,-2}<br><br>Nur Personen, die in den letzten 7 Tagen keine Spirituosen (+KA) getrunken haben (TALKO05a/38.40=2,-2). Die anderen weiter zu 38.50<br>---<br>Haben Sie in den letzten 12 Monaten Spirituosen getrunken?<br>---<br>1 - Ja<br>2 - Nein → **038.50**<br>---<br>(-2) - Keine Antwort → **038.50**	Alkoholkonsum<br><br>TALKO05d	TREND 44<br>*SGB92: SALKO05d*
03844<br><br>*PROXY*	LINK : IF 03843={1} AND NOT PROXY<br>---<br>Wie häufig?<br><br>*INT : Helfen beim Antworten, falls nötig*<br>---<br>1 - Jede Woche<br>2 - 2-3 Mal pro Monat<br>3 - Ca. 1 Mal pro Monat<br>4 - Weniger als 1 Mal pro Monat<br>---<br>(-2) - Keine Antwort	Alkoholkonsum<br><br>TALKO05e	TREND 44<br>*SGB92: SALKO03e*
03845<br><br>*PROXY*	LINK : IF 03843={1} AND NOT PROXY<br>---<br>Und wie viel pro Mal?<br><br>*INT : Helfen beim Antworten, falls nötig*<br>---<br>1 - 5-6 Gläschen oder mehr<br>2 - 3-4 Gläschen<br>3 - 2 Gläschen<br>4 - 1 Gläschen	Alkoholkonsum<br><br>TALKO05f	TREND 44<br>*SGB97 Mengenangaben leicht modifiziert*<br>*SGB92: SALKO05f*

	--- (-2) - Keine Antwort		
03850	LINK : IF 03800={1 TO 6} --- Haben Sie in den letzten 7 Tagen Alcopops getrunken? <br>*INT : z.B. Smirnoff Ice, Bacardi Breezer* --- 1 - Ja 2 - Nein → **038.53** --- (-2) - Keine Antwort → **038.53**	Alkoholkonsum <br>TALKO16a	BFS - SGB
03851 <br>*PROXY*	LINK : IF 03850={1} AND NOT PROXY --- Wie häufig? <br>*INT : Helfen beim Antworten, falls nötig* --- 1 - 3 Mal täglich oder mehr 2 - 2 Mal täglich 3 - 1 Mal täglich 4 - Fast täglich 5 - 3 oder 4 Mal in den letzten 7 Tagen 6 - 1 oder 2 Mal in den letzten 7 Tagen --- (-2) - Keine Antwort	Alkoholkonsum <br>TALKO16b	BFS - SGB
03852 <br>*PROXY*	LINK : IF 03850={1} AND NOT PROXY --- Und wieviel pro Mal? <br>*INT : Helfen beim Antworten, falls nötig* --- 1 - 5 kleine Flaschen à 275 ml oder mehr 2 - 3-4 kleine Flaschen à 275 ml 3 - 2 kleine Flaschen à 275 ml 4 - 1 kleine Flasche à 275 ml --- (-2) - Keine Antwort	Alkoholkonsum <br>TALKO16c	BFS - SGB

03853	LINK : IF 03850={2,-2}<br><br>Nur Personen, die in den letzten 7 Tagen keine Alcopops (+KA) getrunken haben (TALKO016a/38.50=2,-2). Die anderen weiter zu 38.60<br>---<br>Haben Sie in den letzten 12 Monaten Alcopops getrunken?<br>---<br>1 - Ja<br>2 - Nein → **038.60**<br>---<br>(-2) - Keine Antwort → **038.60**	Alkoholkonsum<br><br>TALKO16d	BFS - SGB
03854<br><br>*PROXY*	LINK : IF 03853={1} AND NOT PROXY<br>---<br>Wie häufig?<br><br>*INT : Helfen beim Antworten, falls nötig*<br>---<br>1 - Jede Woche<br>2 - 2-3 Mal pro Monat<br>3 - Ca. 1 Mal pro Monat<br>4 - Weniger als 1 Mal pro Monat<br>---<br>(-2) - Keine Antwort	Alkoholkonsum<br><br>TALKO16e	BFS - SGB
03855<br><br>*PROXY*	LINK : IF 03853={1} AND NOT PROXY<br>---<br>Und wie viel pro Mal?<br><br>*INT : Helfen beim Antworten, falls nötig*<br>---<br>1 - 5 kleine Flaschen à 275 ml oder mehr<br>2 - 3-4 kleine Flaschen à 275 ml<br>3 - 2 kleine Flaschen à 275 ml<br>4 - 1 kleine Flasche à 275 ml<br>---<br>(-2) - Keine Antwort	Alkoholkonsum<br><br>TALKO16f	BFS - SGB
03861<br><br>*PROXY*	LINK : IF 03800={1 TO 7} AND SEX=1 AND NOT PROXY<br>---<br>Wie häufig in den letzten 12 Monaten haben Sie 5 oder mehr Gläser alkoholische Getränke bei einem Anlass oder einer Gelegenheit getrunken?<br><br>*INT : "Gelegenheit" das heisst an einem Abend, an einer Party, einem Fest oder auch alleine.*	Alkoholkonsum<br><br>TALKO23a	Sucht Schweiz (G. Gmel) analog zur SGB07<br>*SGB07: TALKO21*<br>*SGB12: TALKO22*

	Alkoholische Getränke = Bier, Wein, Sekt, Schnaps, Alcopops etc.<br>*Helfen beim Antworten, falls nötig*<br>--<br>1 - Nie<br>2 - Weniger als 1 Mal pro Monat<br>3 - Jeden Monat<br>4 - Jede Woche<br>5 - Jeden Tag oder fast jeden Tag<br>--<br>(-2) - Keine Antwort		
03862<br><br>*PROXY*	LINK : IF 03800={1 TO 7} AND SEX=2 AND NOT PROXY<br>--<br>Wie häufig in den letzten 12 Monaten haben Sie 4 oder mehr Gläser alkoholische Getränke bei einem Anlass oder einer Gelegenheit getrunken?<br><br>*INT : "Gelegenheit" das heisst an einem Abend, an einer Party, einem Fest oder auch alleine.*<br>*Alkoholische Getränke = Bier, Wein, Sekt, Schnaps, Alcopops etc.*<br>*Helfen beim Antworten, falls nötig*<br>--<br>1 - Nie<br>2 - Weniger als 1 Mal pro Monat<br>3 - Jeden Monat<br>4 - Jede Woche<br>5 - Jeden Tag oder fast jeden Tag<br>--<br>(-2) - Keine Antwort	Alkoholkonsum<br><br>TALKO23b	Sucht Schweiz (G. Gmel)<br>analog zur SGB07<br>*SGB07: TALKO21*<br>*SGB12: TALKO22*
03900	LINK : ALL<br>--<br>Rauchen Sie, wenn auch nur selten?<br><br>*INT : Elektronische Zigaretten und iQOS zählen auch dazu*<br>--<br>1 - Ja<br>2 - Nein → **039.20**<br>--<br>(-2) - Keine Antwort → **039.20**	Tabakkonsum<br><br>TTAKO01	Anal. IGIP tel. 28.00
03901	LINK : IF 03900={1}<br>--<br>Rauchen Sie täglich?	Tabakkonsum<br><br>TTAKO21	EHIS angepasst

	--<br>1 - Ja<br>2 - Nein<br>--<br>(-2) - Keine Antwort		
03910	LINK : IF 03900={1}<br>--<br>Was rauchen Sie?<br><br>a) Zigaretten<br>b) Zigarren<br>c) Zigarillos<br>d) Pfeife<br>e) Wasserpfeife, Shisha<br>f) elektronische Zigaretten mit Nikotin<br>g) elektronische Zigaretten ohne Nikotin<br>h) erhitzte Tabakprodukte wie iQOS (Pax etc…)<br><br>*INT : Items vorlesen!*<br>*Selbstgedrehte Zigaretten zählen zu Zigaretten*<br>--<br>1 - Ja<br>2 - Nein<br>--<br>(-2) - Keine Antwort	Tabakkonsum<br><br><br>TTAKO02a<br>TTAKO02b<br>TTAKO02c<br>TTAKO02d<br>TTAKO02e<br>TTAKO02f<br>TTAKO02g<br>TTAKO02h	IGIP tel. 28.10<br>*SGB07: mit TTAKO02e ergänzt*<br>*SGB17: mit TTAKO02f, TTAKO02g und TTAKO02h ergänzt*
03911<br><br><br>*PROXY*	LINK : IF 03910_a={1} AND NOT PROXY<br><br>Personen, die Zigaretten rauchen (ttako02a/39.10=1)<br>--<br>Wieviele Zigaretten rauchen Sie im Durchschnitt pro Tag?<br><br><xx> Zigaretten<br><0> Weniger als 1 Zigarette pro Tag<br><br>*INT : 1 Paket = 20 Zigaretten / 1/2Paket =10 Zigaretten*<br>--<br>(-2) - Keine Antwort	Tabakkonsum<br><br><br>TTAKO03	IGIP tel. 28.11
03912<br><br><br>*PROXY*	LINK : IF 03910_b={1} AND NOT PROXY<br><br>Personen, die Zigarren rauchen (ttako02b/39.10=1)	Tabakkonsum<br><br><br>TTAKO04	IGIP tel. 28.12

	---<br>Wie viele Zigarren rauchen Sie im Durchschnitt pro Tag?<br><br><xx> Zigarren<br><0> Weniger als 1 Zigarre pro Tag<br>---<br>(-2) - Keine Antwort		
03913<br><br>*PROXY*	LINK : IF 03910_c={1} AND NOT PROXY<br><br>Personen, die Zigarillos rauchen (ttako02c/39.10=1)<br>---<br>Wie viele Zigarillos rauchen Sie im Durchschnitt pro Tag?<br><br><xx> Zigarillos<br><0> Weniger als 1 Zigarillo pro Tag<br>---<br>(-2) - Keine Antwort	Tabakkonsum<br><br>TTAKO05	IGIP tel. 28.13
03914<br><br>*PROXY*	LINK : IF 03910_d={1} AND NOT PROXY<br><br>Personen, die Pfeife rauchen (ttako02d/39.10=1)<br>---<br>Wie viele Pfeifen rauchen Sie im Durchschnitt pro Tag?<br><br><xx> Pfeifen<br><0> Weniger als 1 Pfeife pro Tag<br>---<br>(-2) - Keine Antwort	Tabakkonsum<br><br>TTAKO06	IGIP tel. 28.14
03915<br><br>*PROXY*	LINK : IF 03910_f={1} AND NOT PROXY<br><br>Personen, die elektronische Zigaretten mit Nikotin benutzen (TTAKO02f/39.10=1)<br>---<br>Wie oft benützen Sie elektronische Zigaretten mit Nikotin?<br><br>*INT : Helfen beim Antworten, falls nötig*<br>---<br>1 - Täglich<br>2 - Mindestens 1 Mal pro Woche<br>3 - Mindestens 1 Mal pro Monat<br>4 - Weniger als 1 Mal pro Monat	Tabakkonsum<br><br>TTAKO23	BAG Suchtmonitoring-Corolar Split B, Welle 9, 2015 (Frage angepasst) und ITC Canada (International Tobacco Control) (Antwortkategorien angepasst)

	---<br>(-2) - Keine Antwort		
03916<br><br>*PROXY*	LINK : IF 03910_f={1} AND NOT PROXY<br><br>Personen, die elektronische Zigaretten mit Nikotin benutzen<br>---<br>Wieviel Milliliter Flüssigkeit für elektronische Zigaretten oder wieviele elektronische Einweg-Zigaretten verbrauchen Sie durchschnittlich pro Monat?<br><br><xx> ml pro Monat (TTAKO24a)<br><0> Weniger als 1 ml pro Monat (TTAKO24a)<br><xx> Einweg-Zigaretten pro Monat (TTAKO24b)<br><0> Weniger als 1 Einweg-Zigarette pro Monat (TTAKO24b)<br>---<br>(-2) - Keine Antwort	Tabakkonsum<br><br>TTAKO24a<br>TTAKO24b	BFS - HABE 2015 (angepasst)
03917<br><br>*PROXY*	LINK : IF 03910_g={1} AND NOT PROXY<br><br>Personen, die elektronische Zigaretten ohne Nikotin benutzen (TTAKO02g /39.10=1)<br>---<br>Wie oft benützen Sie derzeit elektronische Zigaretten ohne Nikotin?<br><br>*INT : Antwortmöglichkeiten vorlesen!*<br>---<br>1 - Täglich<br>2 - Mindestens 1 Mal pro Woche<br>3 - Mindestens 1 Mal pro Monat<br>4 - Weniger als 1 Mal pro Monat<br>---<br>(-2) - Keine Antwort	Tabakkonsum<br><br>TTAKO25	BAG Suchtmonitoring-Corolar Split B, Welle 9, 2015 (Frage angepasst) und ITC Canada (International Tobacco Control) (Antwortkategorien angepasst)
03918<br><br>*PROXY*	LINK : IF 03910_h={1} AND NOT PROXY<br><br>Personen, die erhitzte Tabakprodukte (iQOS) konsumieren (TTAKO02h /39.10=1)<br>---<br>Wie viele Sticks (Heatsticks) rauchen Sie im Durchschnitt pro Tag?<br><br><xx> Sticks<br><0> Weniger als 1 Stick pro Tag<br>---<br>(-2) - Keine Antwort	Tabakkonsum<br><br>TTAKO26	BFS - SGB

03920	LINK : IF 03900={2,-2}<br><br>Nur Nichtraucher/innen (+KA) (TTAKO01/39.00=2, -2). Raucher/innen weiter zu Frage 39.30<br>---<br>Haben Sie je regelmässig während mehr als 6 Monaten geraucht?<br>---<br>1 - Ja<br>2 - Nein → **039.50**<br>---<br>(-2) - Keine Antwort → **039.50**	Tabakkonsum<br><br><br>TTAKO07	IGIP tel. 28.30
03921	LINK : IF 03920={1}<br>---<br>Wann haben Sie aufgehört zu rauchen?<br><br>*INT : Helfen beim Antworten, falls nötig*<br>---<br>1 - In den letzten 12 Monaten<br>2 - Vor 1 bis weniger als 2 Jahre<br>3 - Vor 2 bis weniger als 5 Jahre<br>4 - Vor 5 bis weniger als 10 Jahre<br>5 - Vor 10 Jahren oder mehr<br>---<br>(-1) - Weiss nicht<br>(-2) - Keine Antwort	Tabakkonsum<br><br><br>TTAKO27	Enquête santé Belgien 2013 (angepasst)<br>*SGB92-12: TTAKO08*
03930<br><br><br>*PROXY*	LINK : IF (03900={1} OR 03920={1}) AND NOT PROXY<br><br>Nur Raucher/innen und Ex-Raucher/innen (TTAKO01/39.00=1 oder TTAKO07/39.20=1)<br>---<br>In welchem Alter haben Sie mit regelmässigem Rauchen angefangen?<br><br><xx> Alter in Jahren<br>---<br>(-2) - Keine Antwort	Tabakkonsum<br><br><br>TTAKO09	BFS - SGB<br>*ESS97: population très légerement différente par raport aux autres enquêtes. Le nom de variable reste inchangé*
03940	LINK : IF 03900={1}<br><br>Nur Raucher/innen (TTAKO01/39.00=1), die anderen weiter zu Frage 39.50<br>---<br>Haben Sie in den letzten 12 Monaten ernsthaft versucht, das Rauchen aufzugeben, das heisst mindestens 14 Tage nicht geraucht?	Tabakkonsum<br><br><br>TTAKO10	IGIP tel 28.20<br>*ESS97: population très légerement différente par raport aux autres enquêtes. Le nom de variable reste inchangé*

	--<br>1 - Ja, habe versucht aufzuhören → **039.50**<br>2 - Nein<br>--<br>(-2) - Keine Antwort		
03941<br><br>*PROXY*	LINK : IF 03940={2,-2} AND NOT PROXY<br>--<br>Möchten Sie gerne aufhören zu rauchen?<br>--<br>1 - Ja<br>2 - Nein<br>--<br>(-2) - Keine Antwort	Tabakkonsum<br><br>TTAKO11	IGIP tel 28.21<br>*ESS97: population très légerement différente par raport aux autres enquêtes. Le nom de variable reste inchangé*
03950<br><br>*PROXY*	LINK : ALL AND NOT PROXY<br><br>Wieder an alle<br>--<br>Wie lange sind Sie täglich dem Tabakrauch von anderen Leuten ausgesetzt?<br><br><xx> Stunden pro Tag (max. 24 Std.) (TTAKO22a)<br><xx> Minuten pro Tag (0-59) (TTAKO22b)<br><br>*INT : Passivrauchen*<br>--<br>(-1) - Weiss nicht<br>(-2) - Keine Antwort	Tabakkonsum<br>Passivrauchen<br><br>[TTAKO22a]<br>[TTAKO22b]<br>TTAKO22	SAPALDIA<br>*SGB07: Minuten pro Tag hinzugefügt. Variable in Datenbank ist in Minuten*
04000	LINK : IF ALTER<=74<br><br>Drogenfragen nur an Personen im Alter von 15 - 74 Jahren! Männer ab 75 Jahren weiter zur Frage 41.51, Frauen ab 75 Jahren weiter zur Frage 41.50<br>--<br>Haben Sie in Ihrem Leben schon einmal Drogen, z.B. Haschisch, Kokain oder andere Drogen genommen?<br>--<br>1 - Ja<br>2 - Nein → **041.00**<br>--<br>(-2) - Keine Antwort → **041.00**	Drogenkonsum<br><br>TDROG01	SFA - WHO 90<br>*SGB92 + 97: 15-59 Jahre*<br>*SGB02: 15- 64 Jahre*<br>*SGB07: 15-69 Jahre*<br>*SGB12 + 17: 15- 74 Jahre*
04010	LINK : IF 04000={1}	Drogenkonsum	ISPM - 10.91<br>*SGB92: 15-39 Jahre*<br>*SGB97: 15- 59 Jahre*

	--<br>Haben Sie schon einmal Cannabis (Haschisch/Marijuana) genommen?<br>--<br>1 - Ja<br>2 - Nein → **040.20**<br>--<br>(-2) - Keine Antwort → **040.20**	TDROG02	*SGB02: 15- 64 Jahre*<br>*SGB07: 15- 69 Jahre*<br>*SGB12-17: 15-74 Jahre*
04012<br><br>*PROXY*	LINK : IF 04010={1} AND NOT PROXY<br>--<br>In welchem Alter haben Sie zum ersten Mal Cannabis (Haschisch/Marijuana) genommen?<br><br><xx> - Alter in Jahren<br>--<br>(-2) - Keine Antwort	Drogenkonsum<br><br>TDROG03	ISPM - 10.91<br>*SGB92: 15-39 Jahre*<br>*SGB97: 15- 59 Jahre*<br>*SGB02: 15- 64 Jahre*<br>*SGB07: 15- 69 Jahre*<br>*SGB12-17: 15-74 Jahre*
04013	LINK : IF 04010={1}<br><br>Nur Personen, die Cannabis konsumiert haben (+KA) (TDROG02=1, -2).). Die Anderen, weiter zu Frage 40.20<br>--<br>Haben Sie in den letzten 12 Monaten Cannabis (Haschisch / Marijuana) genommen?<br>--<br>1 - Ja<br>2 - Nein → **040.20**<br>--<br>(-2) - Keine Antwort → **040.20**	Drogenkonsum<br><br>TDROG78	BFS - SGB<br>*SGB12-17: 15-74 Jahre*<br>*SGB17: Unterschiedliche Referenzpopulation*<br>*SGB12 : TDROG67*
04016	LINK : IF 04013={1}<br><br>Nur Personen, die in den letzten 12 Monaten Cannabis konsumiert haben (TDROG78/40.13 = 1). Die Anderen weiter zur Frage 40.20<br>--<br>Haben Sie in den letzten 30 Tagen Cannabis (Haschisch / Marijuana) genommen?<br>--<br>1 - Ja<br>2 - Nein → **040.20**<br>--<br>(-2) - Keine Antwort → **040.20**	Drogenkonsum<br><br>TDROG79	CoRolAR Core Welle 7, 2014<br>*SGB17: 15-74 Jahre*
04017<br><br>*PROXY*	LINK : IF 04016={1} AND NOT PROXY<br><br>Nur Personen, die in den letzten 30 Tagen Cannabis konsumiert haben (TDROG79/40.16 = 1). Die Anderen weiter zur Frage 40.20<br>--<br>Während den letzten 30 Tagen, wie häufig haben Sie Cannabis (Haschisch / Marijuana) genommen?	Drogenkonsum<br><br>TDROG80	CoRolAR Core Welle 7, 2014<br>*SGB17: 15-74 Jahre*

	INT : Helfen beim Antworten, falls nötig<br>---<br>1 - Täglich oder fast täglich<br>2 - Mehrmals pro Woche<br>3 - Einmal pro Woche<br>4 - Seltener<br>---<br>(-2) - Keine Antwort		
04020	LINK : IF 04000={1}<br>---<br>Haben Sie in Ihrem Leben schon einmal andere Drogen wie z.B. Ecstasy, Kokain, Heroin, usw. genommen?<br>---<br>1 - Ja<br>2 - Nein → **041.00**<br>---<br>(-2) - Keine Antwort → **041.00**	Drogenkonsum<br><br>TDROG50	BFS - SGB<br>*SGB97: 15- 59 Jahre*<br>*SGB02: 15- 64 Jahre*<br>*SGB07: 15- 69 Jahre*<br>*SGB12-17 : 15-74 Jahre*
04030	LINK : IF 04020={1}<br>---<br>Haben Sie schon einmal Ecstasy genommen?<br>---<br>1 - Ja<br>2 - Nein → **040.40**<br>---<br>(-2) - Keine Antwort → **040.40**	Drogenkonsum<br><br>TDROG51	BFS - SGB<br>*SGB97: 15- 49 Jahre*<br>*SGB02: 15- 64 Jahre*<br>*SGB07: 15- 69 Jahre*<br>*SGB12-17 : 15-74 Jahre*
04031	LINK : IF 04030={1}<br>---<br>Haben Sie in den letzten 12 Monaten Ecstasy genommen?<br>---<br>1 - Ja<br>2 - Nein → **040.40**<br>---<br>(-2) - Keine Antwort → **040.40**	Drogenkonsum<br><br>TDROG70	BFS - SGB<br>*SGB12-17 : 15-74 Jahre*
04033	LINK : IF 04031={1}<br><br>Nur Personen, die in den letzten 12 Monaten Ecstasy konsumiert haben (TDROG70/40.31 = 1). Die Anderen weiter zur Frage 40.40<br>---<br>Haben Sie in den letzten 30 Tagen Ecstasy genommen?	Drogenkonsum<br><br>TDROG81	CoRolAR Core Welle 7, 2014<br>*SGB17 : 15-74 Jahre*

	--<br>1 - Ja<br>2 - Nein<br>--<br>(-2) - Keine Antwort		
04040	LINK : IF 04020={1}<br>--<br>Haben Sie schon einmal Kokain genommen?<br>--<br>1 - Ja<br>2 - Nein → **040.50**<br>--<br>(-2) - Keine Antwort → **040.50**	Drogenkonsum<br><br>TDROG14	ISPM - 10.91<br>*SGB92: 15-39 Jahre*<br>*SGB97: 15- 49 Jahre*<br>*SGB02: 15- 64 Jahre*<br>*SGB07: 15- 69 Jahre*<br>*SGB12-17 : 15-74 Jahre*
04041	LINK : IF 04040={1}<br>--<br>Haben Sie in den letzten 12 Monaten Kokain genommen?<br>--<br>1 - Ja<br>2 - Nein → **040.50**<br>--<br>(-2) - Keine Antwort → **040.50**	Drogenkonsum<br><br>TDROG72	BFS - SGB<br>*SGB12-17 : 15-74 Jahre*
04043	LINK : IF 04041={1}<br><br>Nur Personen, die in den letzten 12 Monaten Kokain konsumiert haben (TDROG72/40.41 = 1). Die Anderen weiter zur Frage 40.50<br>--<br>Haben Sie in den letzten 30 Tagen Kokain genommen?<br>--<br>1 - Ja<br>2 - Nein → **040.50**<br>--<br>(-2) - Keine Antwort → **040.50**	Drogenkonsum<br><br>TDROG82	CoRolAR Core Welle 7, 2014<br>*SGB17 : 15-74 Jahre*
04044<br><br>*PROXY*	LINK : IF 04043={1} AND NOT PROXY<br><br>Nur Personen, die in den letzten 30 Tagen Kokain konsumiert haben (TDROG82/40.43 = 1). Die Anderen weiter zur Frage 40.50<br>--<br>Während den letzten 30 Tagen, wie häufig haben Sie Kokain genommen?<br><br>*INT : Helfen beim Antworten, falls nötig*	Drogenkonsum<br><br>TDROG83	CoRolAR Core Welle 7, 2014<br>*SGB17 : 15-74 Jahre*

	--<br>1 - Täglich oder fast täglich<br>2 - Mehrmals pro Woche<br>3 - Einmal pro Woche<br>4 - Seltener<br>--<br>(-2) - Keine Antwort		
04050	LINK : IF 04020={1}<br>--<br>Haben Sie schon einmal Heroin genommen?<br>--<br>1 - Ja<br>2 - Nein → **040.60**<br>--<br>(-2) - Keine Antwort → **040.60**	Drogenkonsum<br><br>TDROG08	ISPM - 10.91<br>*SGB92: 15-39 Jahre*<br>*SGB97: 15- 49 Jahre*<br>*SGB02: 15- 64 Jahre*<br>*SGB07: 15- 69 Jahre*<br>*SGB12-17 : 15-74 Jahre*
04051	LINK : IF 04050={1}<br>--<br>Haben Sie in den letzten 12 Monaten Heroin genommen?<br>--<br>1 - Ja<br>2 - Nein → **040.60**<br>--<br>(-2) - Keine Antwort → **040.60**	Drogenkonsum<br><br>TDROG74	BFS - SGB<br>*SGB12-17 : 15-74 Jahre*
04053	LINK : IF 04051={1}<br><br>Nur Personen, die in den letzten 12 Monaten Heroin konsumiert haben (TDROG74/40.51 = 1). Die Anderen weiter zur Frage 40.60<br>--<br>Haben Sie in den letzten 30 Tagen Heroin genommen?<br>--<br>1 - Ja<br>2 - Nein<br>--<br>(-2) - Keine Antwort	Drogenkonsum<br><br>TDROG84	CoRolAR Core Welle 7, 2014<br>*SGB17 : 15-74 Jahre*
04060	LINK : IF 04020={1}<br>--<br>Haben Sie schon einmal andere Drogen, wie z.B. Speed, Amphetamin, LSD, oder halluzinogene Pilze genommen?<br>--<br>1 - Ja<br>2 - Nein → **041.00**	Drogenkonsum<br><br>TDROG85	ISPM - 10.91<br>*SGB07 TDROG38+57 in einer Variable zusammengefasst*<br>*SGB17: reformulation de l'énoncé de la question*

	--<br>(-2) - Keine Antwort → **041.00**		*SGB92: 15-39 Jahre*<br>*SGB97: 15- 49 Jahre*<br>*SGB02: 15- 64 Jahre*<br>*SGB07: 15- 69 Jahre*<br>*SGB12-17 : 15-74 Jahre*<br>*SGB07+12: TDROG64*
04061	LINK : IF 04060={1}<br>--<br>Haben Sie in den letzten 12 Monaten solche andere Drogen genommen?<br><br>*INT : z.B. Speed, Amphetamin, LSD oder halluzinogene Pilze*<br>--<br>1 - Ja<br>2 - Nein → **041.00**<br>--<br>(-2) - Keine Antwort → **041.00**	Drogenkonsum<br><br>TDROG86	BFS - SGB<br>*SGB17: reformulation de l'énoncé*<br>*SGB12+17 : 15-74 Jahre*<br>*SGB12: TDROG76*
04063	LINK : IF 04061={1}<br><br>Nur Personen, die in den letzten 12 Monaten andere Drogen konsumiert haben (TDROG86/40.61 = 1). Die Anderen weiter zur Frage 41.00<br>--<br>Haben Sie in den letzten 30 Tagen solche andere Drogen genommen?<br>--<br>1 - Ja<br>2 - Nein<br>--<br>(-2) - Keine Antwort	Drogenkonsum<br><br>TDROG87	CoRolAR Core Welle 7, 2014<br>*SGB17 : 15-74 Jahre*
i4100<br><br>*PROXY*	LINK : IF ALTER>=16 AND ALTER<=74 AND NOT PROXY<br><br>Nur an Personen zwischen 16 und 74 Jahren, 15-Jährige weiter zur Frage 43.00, 75-jährige und Ältere weiter zur Frage 43.00<br>--<br>Es kommen nun ein paar Fragen zu Ihrem Sexualverhalten.<br><br>*INT : Bei den nachfolgenden Fragen geht es um die Prävention von AIDS und sexuell übertragbaren Infektionen.*<br>*Argumente wenn Problem: Die nachfolgenden Fragen betreffen die AIDS-Prävention. Gegenwärtig tragen in der Schweiz mehr als 25'000 Personen das HI-Virus und ungefähr 7000 Personen sind bereits daran gestorben. Jeden Tag kommen wieder neue Personen mit dem Virus in Kontakt und riskieren sich anzustecken. Die nächsten Fragen, die ich Ihnen stelle, sind sehr intim. Selbst wenn Sie denken, dass Sie das nicht betrifft, antworten Sie dennoch auf alle Fragen. Ich erinnere Sie nochmals daran, dass alle Antworten strikt anonym bleiben.*	INTRO HIV/AIDS<br><br>TAIDS_a	BAG (A. Jeannin)

	--<br>1 - OK, weiter<br>2 - Verweigerung		
04100<br><br>*PROXY*	LINK : IF i4100={1}<br>--<br>Haben Sie schon einmal Sex gehabt? (Damit meinen wir Geschlechtsverkehr mit Eindringen.)<br>--<br>1 - Ja<br>2 - Nein → **041.50**<br>--<br>(-2) - Keine Antwort → **041.50**	AIDS-Prävention Sexualverhalten<br><br>TAIDS01	EPSS Qu. 25200 - BAG (A. Jeannin)
04101<br><br>*PROXY*	LINK : IF 04100={1}<br>--<br>In welchem Alter haben Sie das erste Mal Sex gehabt?<br><br><xx> Alter in Jahren<br>--<br>(-1) - Weiss nicht<br>(-2) - Keine Antwort	AIDS-Prävention Sexualverhalten<br><br>TAIDS02	EPSS Qu. 25300 - BAG (A. Jeannin)
04110<br><br>*PROXY*	LINK : IF 04100={1}<br>--<br>Haben Sie in den letzten 12 Monaten Sex gehabt?<br>--<br>1 - Ja<br>2 - Nein → **041.20**<br>--<br>(-2) - Keine Antwort → **041.20**	AIDS-Prävention Sexualverhalten<br><br>TAIDS15	BAG (A. Jeannin)
04111<br><br>*PROXY*	LINK : IF 04110={1}<br>--<br>Wie oft haben Sie durchschnittlich in den letzten 12 Monaten Sex gehabt?<br><br>*INT : Antwortmöglichkeiten vorlesen!*<br>--<br>1 - 1-2 Mal im Jahr<br>2 - 1 Mal im Monat<br>3 - 2-3 Mal im Monat<br>4 - 1 Mal die Woche<br>5 - 2-3 Mal die Woche<br>6 - 4 Mal oder mehr die Woche	AIDS-Prävention Sexualverhalten<br><br>TAIDS16	BAG (A. Jeannin)

	---<br>(-2) - Keine Antwort		
04112<br><br>*PROXY*	LINK : IF 04110={1}<br>---<br>Mit wie vielen Personen haben Sie in den letzten 12 Monaten Sex gehabt?<br>Fester Partner/feste Partnerin, Ehemann oder Ehefrau zählt auch als 1 Person.<br><br><xxx> Partner/innen<br><br>*INT : Falls nötig beim Schätzen helfen!*<br>---<br>(-1) - Weiss nicht<br>(-2) - Keine Antwort	AIDS-Prävention Sexualverhalten<br><br>TAIDS17	BAG (A. Jeannin)
04120<br><br>*PROXY*	LINK : IF 04100={1}<br>---<br>Haben Sie beim letzten Mal, wo Sie Sex gehabt haben, ein Präservativ (Kondom / Pariser) verwendet?<br>---<br>1 - Ja<br>2 - Nein<br>3 - Erinnert sich nicht<br>---<br>(-2) - Keine Antwort	AIDS-Prävention Sexualverhalten<br><br>TAIDS18	EPSS Qu. 26600 - BAG (A. Jeannin)<br>*SGB17: unterschiedliche Referenzpopulation*
04130<br><br>*PROXY*	LINK : IF 04100={1}<br>---<br>Bei der folgenden Frage können Sie mit der Antwortnummer antworten. Das letzte Mal, wo Sie Sex gehabt haben, ist das "1" mit Ihrem festen Partner / Ihrer festen Partnerin, "2" mit Gelegenheitspartner/in, oder "3" mit einer Prostituierten / einem Prostituierten gewesen?<br>---<br>1 - 1: Feste/r Partner/in<br>2 - 2: Gelegenheitspartner/in<br>3 - 3: Prostituierte/r<br>---<br>(-2) - Keine Antwort	AIDS-Prävention Sexualverhalten<br><br>TAIDS19	BAG (A. Jeannin)<br>*SGB17: unterschiedliche Referenzpopulation*
04141<br><br>*PROXY*	LINK : IF 04100={1} AND SEX={1}<br><br>Frage für Männer, Frauen weiter zur Frage 41.40<br>---<br>Bei der folgenden Frage können Sie mir wieder die entsprechende Antwortnummer angeben. Haben Sie in den letzten 5 Jahren Sex gehabt ...<br><br>*INT : Antwortmöglichkeiten vorlesen!*	AIDS-Prävention Sexualverhalten<br><br>TAIDS24	EPSS Qu. 25530 - BAG (A. Jeannin)<br>*SGB17: énoncé et réponses de la questions adaptés; population différente*

	---<br>1 - 1: nur mit Frauen<br>2 - 2: hauptsächlich mit Frauen, aber auch mit mindestens einem Mann<br>3 - 3: mit gleich viel Frauen wie Männern<br>4 - 4: hauptsächlich mit Männern, aber auch mit mindestens einer Frau<br>5 - 5: nur mit Männern<br>6 - 6: Keinen Sexualkontakt in den letzten fünf Jahren<br>---<br>(-2) - Keine Antwort		
04140<br><br>*PROXY*	LINK : IF 04100={1} AND SEX={2}<br><br>Frage für Frauen, Männer weiter zur Frage 41.50<br>---<br>Bei der folgenden Frage können Sie mir wieder die entsprechende Antwortnummer angeben. Haben Sie in den letzten 5 Jahren Sex gehabt …<br><br>*INT : Antwortmöglichkeiten vorlesen!*<br>---<br>1 - 1: nur mit Männern<br>2 - 2: hauptsächlich mit Männern, aber auch mit mindestens einer Frau<br>3 - 3: mit gleich viel Männern wie Frauen<br>4 - 4: hauptsächlich mit Frauen, aber auch mit mindestens einem Mann<br>5 - 5: nur mit Frauen<br>6 - 6: Keinen Sexualkontakt in den letzten fünf Jahren<br>---<br>(-2) - Keine Antwort → **042.10**	AIDS-Prävention<br>Sexualverhalten<br><br><br>TAIDS25	EPSS Qu. 25520 - BAG (A. Jeannin)<br>*SGB17: énoncé et réponses de la questions adaptés; population différente*
04150<br><br>*PROXY*	LINK : IF 04141={2 TO 6} OR<br>04100={2,-2} AND SEX={1}<br>---<br>Bei der folgenden Frage können Sie wieder mit der Antwortnummer antworten.<br>Betrachten Sie sich als…<br><br>*INT : Antwortmöglichkeiten vorlesen!*<br>---<br>1 - 1: Heterosexuell (fühlt sich sexuell zu Frauen hingezogen)<br>2 - 2: Schwul, das heisst gay (fühlt sich sexuell zu Männern hingezogen)<br>3 - 3: Bisexuell (fühlt sich sexuell sowohl zu Männern als auch zu Frauen hingezogen)<br>4 - (Nicht vorlesen, codieren falls spontane Aussage) Andere<br>---<br>(-1) - Weiss nicht<br>(-2) - Keine Antwort	AIDS-Prävention<br>Sexualverhalten<br><br><br>TAIDS22	ONS (UK) - Questions on sexual identity;<br>Angepasst durch IUMSP (Bize)

04151<br><br>*PROXY*	LINK : IF 04140={2 TO 6} OR<br>04100={2,-2} AND SEX={2}<br>--<br>Bei der folgenden Frage können Sie wieder mit der Antwortnummer antworten.<br>Betrachten Sie sich als…<br><br>*INT : Antwortmöglichkeiten vorlesen!*<br>--<br>1 - 1: Heterosexuell (fühlt sich sexuell zu Männern hingezogen)<br>2 - 2: Lesbisch (fühlt sich sexuell zu Frauen hingezogen)<br>3 - 3: Bisexuell (fühlt sich sexuell sowohl zu Frauen als auch zu Männern hingezogen)<br>4 - (Nicht vorlesen, codieren falls spontane Aussage) Andere<br>--<br>(-1) - Weiss nicht<br>(-2) - Keine Antwort	AIDS-Prävention<br>Sexualverhalten<br><br><br>TAIDS23	ONS (UK) - Questions on sexual identity;<br>Angepasst durch IUMSP (Bize)
04210<br><br>*PROXY*	LINK : IF i4100={1}<br>--<br>Wie viele Male haben Sie bisher den Aids-Test schon machen lassen?<br><br><xxx> - Anzahl Mal<br>--<br>0 - Nie → **042.00**<br>--<br>(-1) - Weiss nicht → **042.00**<br>(-2) - Keine Antwort → **042.00**	AIDS-Prävention<br>HIV-Test<br><br><br>THIV12	EPSS Qu. 15500 - BAG (A. Jeannin)<br>*SGB97: THIV02 15-49 Jahre*<br>*SGB02: THIV02 15+ Jahre*<br>*SGB07+12: THIV07 / THIV08*<br>*SGB17 : THIV07 + THIV08 zusammen*
04212<br><br>*PROXY*	LINK : IF 04210>={1}<br><br>Nur an Personen, die einen AIDS-Test gemacht haben (04210/1<=THIV12<998).<br>Die Anderen weiter zu Frage 04200<br>--<br>Und wann das letzte Mal?<br><br>*INT : Antwortmöglichkeiten vorlesen!*<br>--<br>1 - In den letzten 12 Monaten<br>2 - Vor mehr als 12 Monaten<br>--<br>(-1) - Weiss nicht<br>(-2) - Keine Antwort	AIDS-Prävention<br>HIV-Test<br><br><br>THIV13	EPSS Qu. 15700 - BAG (A. Jeannin)<br>*SGB97+02: THIV03a-c*<br>*SGB17: Antwrtskategorien angepasst*

04200<br><br>*PROXY*	LINK : ALTER>=18 AND ALTER<=74 AND NOT PROXY<br><br>Nur an Personen zwischen 18 und 74 Jahren! Die 15-Jährigen bis 17-Jährigen sowie 75-jährige und Ältere weiter zu Frage 43.00<br>---<br>Haben Sie jemals Blut gespendet?<br><br>*INT : Zivil oder im Militärdienst. Wenn Auto-Transfusion „Für sich selber Blut spenden", dann „Ja" eingeben.*<br>---<br>1 - Ja<br>2 - Nein<br>---<br>(-1) - Weiss nicht<br>(-2) - Keine Antwort	AIDS-Prävention<br>HIV-Test<br><br>THIV14	EPSS Qu. 15200 - BAG (A. Jeannin)<br>*SGB97: THIV01*<br>*SGB07-12: THIV05*<br><br>*SGB97: 15+ Jahre*<br>*SGB07 :16-74 Jahre*<br>*SGB12 : 16+ Jahre*<br>*SGB17 : 18-74 Jahre*<br>*SGB17 énoncé de la question adapté*
04213<br><br>*PROXY*	LINK : IF 04200={1} AND ALTER>=50 AND ALTER<=74<br><br>Nur an Personen ab 50 Jahren!<br>---<br>Und wann zum letzten Mal?<br>---<br>1 - In den letzten 12 Monaten<br>2 - Vor 1 und weniger als 5 Jahren<br>3 - Vor 5 und weniger als 10 Jahren<br>4 - Vor 10 und weniger als 30 Jahren<br>5 - Vor 1985<br>---<br>(-1) - Weiss nicht<br>(-2) - Keine Antwort	AIDS-Prävention<br>HIV-Test<br><br>THIV15	BFS - SGB
04300	LINK : ALL<br><br>Wieder an alle<br>---<br>Jetzt möchten wir Ihnen noch ein paar Fragen zu medizinischen Tests stellen.<br>Hat Ihnen ein Arzt oder eine andere Person aus dem medizinischen Bereich schon einmal gesagt, Ihr Blutdruck sei zu hoch?<br><br>*INT : Apotheker zählen auch zu Personen aus dem medizinischen Bereich*<br>---<br>1 - Ja<br>2 - Nein → **043.10**	Blutdruck<br><br>TBLUT06	BFS - SGB

	---<br>(-1) - Weiss nicht → **043.10**<br>(-2) - Keine Antwort → **043.10**		
04302	LINK : IF 04300={1}<br>---<br>Haben Sie in den letzten 12 Monaten einen zu hohen Blutdruck gehabt?<br><br>*INT : Wenn die Person sagt, dass Sie ein Medikament nimmt, mit "Ja" codieren.*<br>---<br>1 - Ja<br>2 - Nein<br>---<br>(-1) - Weiss nicht<br>(-2) - Keine Antwort	Blutdruck<br><br>TBLUT12	BFS - SGB (EHIS angepasst)
04310<br><br>*PROXY*	LINK : ALL AND NOT PROXY<br>---<br>Ist Ihr Blutdruck im Moment normal, zu tief oder zu hoch?<br><br>*INT : Wenn die Person sagt, dass Sie ein Medikament nimmt, die Situation mit Medikament berücksichtigen.*<br>---<br>1 - Normal<br>2 - Zu tief<br>3 - Zu hoch<br>---<br>(-1) - Weiss nicht<br>(-2) - Keine Antwort	Blutdruck<br><br>TBLUT01	IGIP tel 57.00
04320<br><br>*PROXY*	LINK : ALL AND NOT PROXY<br>---<br>Wann ist Ihr Blutdruck das letzte Mal gemessen worden?<br><br>*INT : Helfen beim Antworten, falls nötig*<br>---<br>1 - In den letzten 12 Monaten<br>2 - Vor 1 bis weniger als 3 Jahren<br>3 - Vor 3 bis weniger als 5 Jahren<br>4 - Vor 5 Jahren oder mehr<br>5 - Noch nie<br>---<br>(-1) - Weiss nicht<br>(-2) - Keine Antwort	Blutdruck<br><br>TBLUT13	IGIP tel. 59.00<br>*SGB92-12: TBLUT04a-c*<br>*SGB17: Antwortmodalitäten angepasst*

04400	LINK : ALL<br>---<br>Hat Ihnen ein Arzt oder eine andere Person aus dem medizinischen Bereich schon einmal gesagt, Ihr Cholesterinspiegel (Blutfett) sei zu hoch?<br><br>*INT : Apotheker zählen auch zu Personen aus dem medizinischen Bereich*<br>---<br>1 - Ja<br>2 - Nein → **044.10**<br>---<br>(-1) - Weiss nicht → **044.10**<br>(-2) - Keine Antwort → **044.10**	Cholesterin<br><br>TCHOL05	MONICA
04402	LINK : IF 04400={1}<br>---<br>Haben Sie in den letzten 12 Monaten einen zu hohen Cholesterinspiegel gehabt?<br><br>*INT : Wenn die Person sagt, dass Sie ein Medikament nimmt, mit "Ja" codieren.*<br>---<br>1 - Ja<br>2 - Nein<br>---<br>(-1) - Weiss nicht<br>(-2) - Keine Antwort	Cholesterin<br><br>TCHOL13	BFS - SGB (EHIS angepasst)
04410<br><br>*PROXY*	LINK : ALL AND NOT PROXY<br>---<br>Ist Ihr Cholesterinspiegel im Moment normal oder zu hoch?<br><br>*INT : Wenn die Person sagt, dass Sie ein Medikament nimmt, die Situation mit Medikament berücksichtigen.*<br>---<br>1 - Normal<br>2 - Zu hoch<br>---<br>(-1) - Weiss nicht<br>(-2) - Keine Antwort	Cholesterin<br><br>TCHOL08	IGIP tel. 62.10
04420<br><br>*PROXY*	LINK : ALL AND NOT PROXY<br>---<br>Wann ist der Cholesterinspiegel bei Ihnen das letzte Mal gemessen worden?<br><br>*INT : Helfen beim Antworten, falls nötig*	Cholesterin<br><br>TCHOL14	Anal. IGIP tel. 60.00<br>*SGB92-12: TCHOL01a-c*<br>*SGB17: Antwortmodalitäten angepasst*

	--<br>1 - In den letzten 12 Monaten<br>2 - Vor 1 bis weniger als 3 Jahren<br>3 - Vor 3 bis weniger als 5 Jahren<br>4 - Vor 5 Jahren oder mehr<br>5 - Noch nie<br>--<br>(-1) - Weiss nicht<br>(-2) - Keine Antwort		
04500	LINK : ALL<br>--<br>Hat Ihnen ein Arzt schon einmal gesagt, Sie hätten Diabetes bzw. Sie seien zuckerkrank?<br>--<br>1 - Ja<br>2 - Nein → **045.10**<br>--<br>(-1) - Weiss nicht → **045.10**<br>(-2) - Keine Antwort → **045.10**	Diabetes<br><br>TDIAB01	anal. MONICA
04502	LINK : IF 04500={1}<br>--<br>Um welchen Typ Diabetes hat es sich dabei gehandelt?<br>--<br>1 - Typ I<br>2 - Typ II<br>3 - Ein anderer Typus<br>--<br>(-1) - Weiss nicht<br>(-2) - Keine Antwort	Diabetes<br><br>TDIAB11	BFS - SGB (EHIS angepasst)
04503	LINK : IF 04500={1}<br>--<br>Haben Sie in den letzten 12 Monaten Diabetes gehabt?<br>--<br>1 - Ja<br>2 - Nein<br>--<br>(-1) - Weiss nicht<br>(-2) - Keine Antwort	Diabetes<br><br>TDIAB12	BFS - SGB (EHIS angepasst)
04510	LINK : ALL AND NOT PROXY	Diabetes	BFS - SGB

PROXY	---<br>Ist Ihr Blutzucker im Moment normal oder zu hoch?<br><br>*INT : Wenn die Person sagt, dass Sie ein Medikament nimmt, die Situation mit Medikament berücksichtigen.*<br>---<br>1 - Normal<br>2 - Zu hoch<br>---<br>(-1) - Weiss nicht<br>(-2) - Keine Antwort	TDIAB08	*Ab SGB02 ist TDIAB01 kein Filter mehr!*
04520<br><br>*PROXY*	LINK : ALL AND NOT PROXY<br>---<br>Wann ist der Blutzucker bei Ihnen das letzte Mal gemessen worden?<br><br>*INT : Helfen beim Antworten, falls nötig*<br>---<br>1 - In den letzten 12 Monaten<br>2 - Vor 1 bis weniger als 3 Jahren<br>3 - Vor 3 bis weniger als 5 Jahren<br>4 - Vor 5 Jahren oder mehr<br>5 - Noch nie<br>---<br>(-1) - Weiss nicht<br>(-2) - Keine Antwort	Diabetes<br><br>TDIAB13	Anal. IGIP tel. 60.00<br>*SGB92-12: TDIAB06a-c*<br>*SGB17: Antwortmodalitäten angepasst*
04600<br><br>*PROXY*	LINK : IF ((SEX=1 AND ALTER>=55) OR (SEX=2 AND ALTER>=45)) AND NOT PROXY<br><br>Nur an Männer ab 55 Jahren und Frauen ab 45 Jahren. Alle Andern weiter zur Frage 47.00<br>---<br>Ist bei Ihnen schon einmal eine Messung von der Knochendichte durchgeführt worden?<br>---<br>1 - Ja<br>2 - Nein → **047.00**<br>---<br>(-1) - Weiss nicht → **047.00**<br>(-2) - Keine Antwort → **047.00**	Osteoporose<br><br>TOSTE01	ISPM BE (M. Zwahlen)<br>*SGB07-12: Männer ab 50 Jahre / Frauen ab 40 Jahre*<br>*SGB17: Männer ab 55 Jahre / Frauen ab 45 Jahre*
04601<br><br>*PROXY*	LINK : IF 04600={1} AND NOT PROXY<br>---<br>Wann ist die Knochendichte bei Ihnen das letzte Mal gemessen worden?<br><br>*INT : Helfen beim Antworten, falls nötig*	Osteoporose<br><br>TOSTE04	ISPM BE (M. Zwahlen)<br>*SGB07-12: TOSTE02a-c*<br>*SGB07-12: Männer ab 50 Jahre / Frauen ab 40 Jahre*<br>*SGB17: Männer ab 55 Jahre*

	---<br>1 - In den letzten 12 Monaten<br>2 - Vor 1 bis weniger als 3 Jahren<br>3 - Vor 3 bis weniger als 5 Jahren<br>4 - Vor 5 Jahren oder mehr<br>---<br>(-1) - Weiss nicht<br>(-2) - Keine Antwort		*/ Frauen ab 45 Jahre SGB17: Antwortsmodalitäten angepasst*
04700<br><br>*PROXY*	LINK : IF SEX=1 AND ALTER>=40 AND NOT PROXY<br><br>Nur an Männer ab 40 Jahren, jüngere Männer weiter zu 05000 und Frauen weiter zu 04800<br>---<br>Haben Sie schon einmal eine Prostata-Untersuchung machen lassen?<br>---<br>1 - Ja<br>2 - Nein → **049.00**<br>---<br>(-1) - Weiss nicht → **049.00**<br>(-2) - Keine Antwort → **049.00**	Krebsfrüherkennung Prostata<br><br>TKREB01	BFS - SGB
04701<br><br>*PROXY*	LINK : IF 04700={1} AND NOT PROXY<br>---<br>Wie ist diese Untersuchung beim letzten Mal gemacht worden?<br><br>*INT : Antwortmöglichkeiten vorlesen!*<br>---<br>1 - Mit einer „rektalen Untersuchung“<br>2 - Mit einem Bluttest (PSA-Test)<br>3 - Mit beidem<br>---<br>(-1) - Weiss nicht<br>(-2) - Keine Antwort	Krebsfrüherkennung Prostata<br><br>TKREB27	ISPM BE (M. Zwahlen)
04702<br><br>*PROXY*	LINK : IF 04700={1} AND NOT PROXY<br>---<br>Was ist beim letzten Mal der Grund für die Prostata-Untersuchung gewesen? Ist das gewesen:<br><br>*INT : Antwortmöglichkeiten vorlesen!*<br>---<br>1 - Als Vorsorgeuntersuchung oder "Check-up", ohne dass Sie vorher Beschwerden oder Symptome gehabt hätten<br>2 - Zur Abklärung von Beschwerden oder Symptomen	Krebsfrüherkennung Prostata<br><br>TKREB28	ISPM BE (M. Zwahlen)<br>*SGB07: 2 Antworten möglich, TKREB28a-b, ab SGB12: nur eine Antwort möglich*

	3 - Zur Nachkontrolle, weil eine frühere Untersuchung etwas angezeigt hat<br>4 - Anderer Grund<br>---<br>(-1) - Weiss nicht<br>(-2) - Keine Antwort		
04710<br><br>*PROXY*	LINK : IF 04700={1} AND NOT PROXY<br>---<br>Wann ist die Prostata-Untersuchung bei Ihnen das letzte Mal durchgeführt worden?<br><br><xx> Monat (TKREB11b)<br><xxxx> Jahr (TKREB11c)<br>---<br>(-1) - Weiss nicht<br>(-2) - Keine Antwort	Krebsfrüherkennung Prostata<br><br>TKREB11b<br>TKREB11c	BFS - SGB<br>*Ab SGB97: Monat eingefügt*
04711<br><br>*PROXY*	LINK : IF ((04710_b={-1,-2} AND 04710_c={2016}) OR (04710_b={-1,-2} AND 04710_c={-1,-2})) AND NOT PROXY<br><br>Falls sich die Person bei der Frage 04710 nicht mehr an das Datum erinnert (TKREB11a =-2, -1) oder falls das Jahr 2016 aber der Monat unbekannt ist (TKREB11c =2016 und TKREB11b =-2, -1)<br>---<br>Ist es in den letzten 12 Monaten gewesen?<br>---<br>1 - Ja<br>2 - Nein<br>---<br>(-1) - Weiss nicht<br>(-2) - Keine Antwort	Krebsfrüherkennung Prostata<br><br>TKREB20	BFS - SGB<br>*Frage um „Missings" zu verhindern*
04800<br><br>*PROXY*	LINK : IF SEX=2 AND ALTER>=20 AND NOT PROXY<br><br>Für Frauen ab 20 J., andere Frauen und Männer weiter zu Frage 49.00<br>---<br>Haben Sie schon einmal einen Gebärmutterhalsabstrich (PAP-Test) machen lassen?<br>---<br>1 - Ja<br>2 - Nein **→ 048.10**<br>---<br>(-1) - Weiss nicht **→ 048.10**<br>(-2) - Keine Antwort **→ 048.10**	Krebsfrüherkennung Gebärmutterhals<br><br>TKREB43	Anal. IGIP tel. 71.00<br>*SGB17: Fragestellung angepasst*
04801	LINK : IF 04800={1} AND NOT PROXY<br>---<br>Wann ist der Gebärmutterhalsabstrich bei Ihnen das letzte Mal durchgeführt worden?	Krebsfrüherkennung Gebärmutterhals	BFS - SGB

PROXY	<xx> Monat (TKREB44b)<br><xxxx> Jahr (TKREB44c)<br>---<br>(-1) - Weiss nicht<br>(-2) - Keine Antwort	TKREB44b<br>TKREB44c	*Ab SGB97: Monat eingefügt*<br>*SGB17: Fragestellung angeppasst*
04803<br><br>*PROXY*	LINK : IF (04801_b={-1,-2} AND 04801_c={-1,-2}) AND NOT PROXY<br><br>FALLS SICH DIE PERSON BEI DER FRAGE 04801 NICHT MEHR AN DAS DATUM ERINNERT (TKREB40A =-2, -1)<br>---<br>Ist es... gewesen?<br><br>*INT : Antwortmöglichkeiten vorlesen!*<br>---<br>1 - In den letzten 12 Monaten<br>2 - Vor 1 bis weniger als 3 Jahre<br>3 - Vor 3 Jahren oder mehr<br>---<br>(-1) - Weiss nicht<br>(-2) - Keine Antwort	Krebsfrüherkennung Gebärmutterhals<br><br>TKREB45	Anal. EHIS 2014<br>*SGB02-SGB12: TKREB21 Frage um Missings zu verhindern*<br>*SGB17: créé plusieurs questions pour éviter les missings et mieux cibler l'année de dépistage*
04804<br><br>*PROXY*	LINK : IF (04801_b={-1,-2} AND 04801_c={2016}) AND NOT PROXY<br><br>falls das Jahr 2016, aber der Monat unbekannt ist (04801/TKREB44c=2016 et TKREB44b=-2, -1)<br>---<br>Ist es in den letzten 12 Monaten gewesen?<br>---<br>1 - Ja<br>2 - Nein<br>---<br>(-1) - Weiss nicht<br>(-2) - Keine Antwort	Krebsfrüherkennung Gebärmutterhals<br><br>TKREB46	Anal. EHIS 2014<br>*SGB02-SGB12: TKREB21 Frage um Missings zu verhindern*<br>*SGB17: créé plusieurs questions pour éviter les missings et mieux cibler l'année de dépistage*
04805<br><br>*PROXY*	LINK : IF (04801_b={-1,-2} AND 04801_c={2014}) AND NOT PROXY<br><br>falls das Jahr 2014, aber der Monat unbekannt ist (04801/TKREB44c=2014 et TKREB44b=-2, -1)<br>---<br>Ist es vor weniger als 3 Jahren gewesen?<br>---<br>1 - Ja<br>2 - Nein	Krebsfrüherkennung Gebärmutterhals<br><br>TKREB47	Anal. EHIS 2014<br>*SGB17: créé plusieurs questions pour éviter les missings et mieux cibler l'année de dépistage*

	---<br>(-1) - Weiss nicht<br>(-2) - Keine Antwort		
04810<br><br>*PROXY*	LINK : IF SEX=2 AND ALTER>=20 AND NOT PROXY<br>---<br>Haben Sie schon einmal eine Mammographie machen lassen?<br><br>*INT : Die Mammographie ist eine Röntgenuntersuchung der weiblichen Brust.*<br>---<br>1 - Ja<br>2 - Nein → **049.00**<br>---<br>(-1) - Weiss nicht → **049.00**<br>(-2) - Keine Antwort → **049.00**	Krebsfrüherkennung Brust<br><br>TKREB05	Anal. IGIP tel 72.00<br>*SGB02: unterschiedliche Referenzpopulation*<br>*Ab SGB07: gleiche Population und vergleichbar mit SGB92 und SGB97*
04811<br><br>*PROXY*	LINK : IF 04810={1} AND NOT PROXY<br>---<br>Wann ist die Mammographie bei Ihnen das letzte Mal gemacht worden?<br><br><xx> Monat (TKREB13b)<br><xxxx> Jahr (TKREB13c)<br>---<br>(-1) - Weiss nicht<br>(-2) - Keine Antwort	Krebsfrüherkennung Brust<br><br>TKREB13b<br>TKREB13c	Anal. IGIP tel. 72.00<br>*Ab 1997: intégration aussi du mois pour la date demandée*<br>*SGB02: unterschiedliche Referenzpopulation*<br>*Ab SGB07: gleiche Population und vergleichbar mit SGB97*
04815<br><br>*PROXY*	LINK : IF (04811_b={-1,-2} AND 04811_c={-1,-2}) AND NOT PROXY<br><br>Falls sich die Person bei der Frage 04811 nicht mehr an das Datum erinnert (TKREB13a =-2, -1)<br>---<br>Ist es... gewesen?<br><br>*INT : Antwortmöglichkeiten vorlesen!*<br>---<br>1 - In den letzten 12 Monaten<br>2 - Vor 1 bis weniger als 2 Jahre<br>3 - Vor 2 Jahren oder mehr<br>---<br>(-1) - Weiss nicht<br>(-2) - Keine Antwort	Krebsfrüherkennung Brust<br><br>TKREB48	Anal. EHIS 2014<br>*SGB02: Frage um Missings zu verhindern / unterschiedliche Population*<br>*SGB07-SGB12: TKREB24*<br>*SGB17: créé plusieurs questions pour éviter les missings et mieux cibler l'année de dépistage*
04816	LINK : IF (04811_b={-1,-2} AND 04811_c={2016}) AND NOT PROXY<br><br>falls das Jahr 2016, aber der Monat unbekannt ist (04811/tkreb13c=2016 et tkreb13b=-2, -1)	Krebsfrüherkennung Brust	Anal. EHIS 2014<br>*SGB02: Frage um Missings zu verhindern /*

PROXY	---<br>Ist es in den letzten 12 Monaten gewesen?<br>---<br>1 - Ja<br>2 - Nein<br>---<br>(-1) - Weiss nicht<br>(-2) - Keine Antwort	TKREB49	*unterschiedliche Population*<br>*SGB07-SGB12: TKREB24*<br>*SGB17: créé plusieurs questions pour éviter les missings et mieux cibler l'année de dépistage*
04817<br><br>*PROXY*	LINK : IF (04811_b={-1,-2} AND 04811_c={2015}) AND NOT PROXY<br><br>falls das Jahr 2015, aber der Monat unbekannt ist (04811/tkreb13c=2015 et tkreb13b=-2, -1)<br>---<br>Ist es vor weniger als 2 Jahren gewesen?<br>---<br>1 - Ja<br>2 - Nein<br>---<br>(-1) - Weiss nicht<br>(-2) - Keine Antwort	Krebsfrüherkennung Brust<br><br>TKREB50	Anal. EHIS 2014<br>*SGB02-SGB12: TKREB21*<br>*Frage um Missings zu verhindern*<br>*SGB17: créé plusieurs questions pour éviter les missings et mieux cibler l'année de dépistage*
04813<br><br>*PROXY*	LINK : IF 04810={1} AND NOT PROXY<br>---<br>Wer hat veranlasst, dass diese Mammographie durchgeführt wird?<br><br>*INT : Vorlesen!*<br>---<br>1 - Sie selbst, auf eigene Initiative<br>2 - Auf Aufforderung vom Arzt, von der Ärztin<br>3 - Im Rahmen von einem Brustkrebs- Früherkennungsprogramm (Mammographie-Screening)<br>---<br>(-1) - Weiss nicht<br>(-2) - Keine Antwort	Krebsfrüherkennung Brust<br><br>TKREB25	BFS - SGB<br>*SGB02: unterschiedliche Population, changé le nom de variable en 2002 (TKREB42) car dès 2007 la variable est comparable avec les enquêtes suivantes*
04814<br><br>*PROXY*	LINK : IF 04810={1} AND NOT PROXY<br>---<br>Was ist beim letzten Mal der Grund für die Mammographie gewesen? Ist das gewesen:<br><br>*INT : Antwortmöglichkeiten vorlesen!*<br>---<br>1 - Als Vorsorgeuntersuchung oder "Check-up", ohne dass Sie vorher Beschwerden oder Symptome gehabt hätten<br>2 - Zur Abklärung von Beschwerden oder Symptomen	Krebsfrüherkennung Brust<br><br>TKREB29	ISPM BE (M. Zwahlen)

	3 - Zur Nachkontrolle, weil eine frühere Mammographie etwas angezeigt hat<br>4 - Anderer Grund<br>---<br>(-1) - Weiss nicht<br>(-2) - Keine Antwort		
04900<br><br>*PROXY*	LINK : IF ALTER>=40 AND NOT PROXY<br><br>Personen ab 40 Jahren. Die jüngeren Personen weiter zur Frage 50.00.<br>---<br>Haben Sie schon einmal einen Test auf verstecktes Blut im Stuhl, d. h. ein Hämoccult-Test machen lassen?<br><br>*INT : Das ist ein Test, bei dem Stuhlproben mit einem speziellen Kärtchen auf unsichtbare Spuren von Blut im Stuhl untersucht werden.*<br>---<br>1 - Ja<br>2 - Nein → **049.10**<br>---<br>(-1) - Weiss nicht → **049.10**<br>(-2) - Keine Antwort → **049.10**	Krebsfrüherkennung Darm<br><br>TKREB30	ISPM BE (M. Zwahlen)
04901<br><br>*PROXY*	LINK : IF 04900={1} AND NOT PROXY<br>---<br>Wann ist dieser Hämoccult-Test bei Ihnen das letzte Mal durchgeführt worden?<br><br><xx> Monat (TKREB31b)<br><xxxx> Jahr (TKREB31c)<br>---<br>(-1) - Weiss nicht<br>(-2) - Keine Antwort	Krebsfrüherkennung Darm<br><br>TKREB31b<br>TKREB31c	ISPM BE (M. Zwahlen)
04904<br><br>*PROXY*	LINK : IF (04901_b={-1,-2} AND 04901_c={-1,-2}) AND NOT PROXY<br><br>Falls sich die Person bei der Frage 049.01 nicht mehr an das Datum erinnert (TKREB31a =-1, -2) oder falls das Jahr 2011 aber der Monat unbekannt ist (TKREB31c =2016 und TKREB31b = -1, -2)<br>---<br>Ist es …gewesen?<br><br>*INT : Antwortmöglichkeiten vorlesen!*<br>---<br>1 - In den letzten 12 Monaten<br>2 - Vor 1 bis weniger als 2 Jahren<br>3 - Vor 2 bis weniger als 5 Jahren<br>4 - Vor 5 Jahren oder mehr	Krebsfrüherkennung Darm<br><br>TKREB51	Anal. EHIS 2014<br>*SGB07-SGB12: TKREB32*<br>*SGB17: créé plusieurs questions pour éviter les missings et mieux cibler l'année de dépistage*

	---<br>(-1) - Weiss nicht<br>(-2) - Keine Antwort		
04905<br><br>*PROXY*	LINK : IF (04901_b={-1,-2} AND 04901_c={2016}) AND NOT PROXY<br><br>falls das Jahr 2016, aber der Monat unbekannt ist (tkreb31c=2016 et tkreb31b=-1)<br>---<br>Ist es in den letzten 12 Monaten gewesen?<br>---<br>1 - Ja<br>2 - Nein<br>---<br>(-1) - Weiss nicht<br>(-2) - Keine Antwort	Krebsfrüherkennung Darm<br><br>TKREB52	Anal. EHIS 2014<br>*SGB07-SGB12: TKREB32*<br>*SGB17: créé plusieurs questions pour éviter les missings et mieux cibler l'année de dépistage*
04906<br><br>*PROXY*	LINK : IF (04901_b={-1,-2} AND 04901_c={2015}) AND NOT PROXY<br><br>falls das Jahr 2015, aber der Monat unbekannt ist (tkreb31c=2015 et tkreb31b=-1)<br>---<br>Ist es vor weniger als 2 Jahren gewesen?<br>---<br>1 - Ja<br>2 - Nein<br>---<br>(-1) - Weiss nicht<br>(-2) - Keine Antwort	Krebsfrüherkennung Darm<br><br>TKREB53	Anal. EHIS 2014<br>*SGB17: créé plusieurs questions pour éviter les missings et mieux cibler l'année de dépistage*
04907<br><br>*PROXY*	LINK : IF (04901_b={-1,-2} AND 04901_c={2012}) AND NOT PROXY<br><br>falls das Jahr 2012, aber der Monat unbekannt ist (tkreb31c=2012 et tkreb31b=-1)<br>---<br>Ist es vor weniger als 5 Jahren gewesen?<br>---<br>1 - Ja<br>2 - Nein<br>---<br>(-1) - Weiss nicht<br>(-2) - Keine Antwort	Krebsfrüherkennung Darm<br><br>TKREB54	Anal. EHIS 2014<br>*SGB17: créé plusieurs questions pour éviter les missings et mieux cibler l'année de dépistage*
04903<br><br>*PROXY*	LINK : IF 04900={1} AND NOT PROXY<br>---<br>Was ist beim letzten Mal der Grund dafür gewesen, dass Sie diesen Hämoccult-Test gemacht haben? Ist das gewesen:	Krebsfrüherkennung Darm	ISPM BE (M. Zwahlen)

	INT : Antwortmöglichkeiten vorlesen!<br>---<br>1 - Als Vorsorgeuntersuchung oder "Check-up", ohne dass Sie vorher Beschwerden oder Symptome gehabt hätten<br>2 - Zur Abklärung von Beschwerden oder Symptomen<br>3 - Zur Nachkontrolle, weil ein früherer Test etwas angezeigt hat<br>4 - Anderer Grund<br>---<br>(-1) - Weiss nicht<br>(-2) - Keine Antwort	TKREB33	
04910<br><br>*PROXY*	LINK : IF ALTER>={40} AND NOT PROXY<br><br>Personen ab 40 Jahren. Die jüngeren Personen weiter zur Frage 05000.<br>---<br>Haben Sie schon einmal eine Darmspiegelung gehabt? Das ist eine Darmuntersuchung, wo man eine dünne Metall-Sonde in den Dickdarm einführt.<br><br>*INT : andere Fachausdrücke: "Endoskopie", "Koloskopie", "Rektoskopie" oder "Sigmoidoskopie", je nach Darmabschnitt, den man untersucht.*<br>---<br>1 - Ja<br>2 - Nein → **050.00**<br>---<br>(-1) - Weiss nicht → **050.00**<br>(-2) - Keine Antwort → **050.00**	Krebsfrüherkennung Darm<br><br><br>TKREB34	ISPM BE (M. Zwahlen)
04911<br><br>*PROXY*	LINK : IF 04910={1} AND NOT PROXY<br>---<br>Wann ist diese Darmspiegelung bei Ihnen zum letzten Mal durchgeführt worden?<br><br><xx> Monat (TKREB35b)<br><xxxx> Jahr (TKREB35c)<br>---<br>(-1) - Weiss nicht<br>(-2) - Keine Antwort	Krebsfrüherkennung Darm<br><br><br>TKREB35b<br>TKREB35c	ISPM BE (M. Zwahlen)
04914<br><br>*PROXY*	LINK : IF (04911_b={-1,-2} AND 04911_c={-1,-2}) AND NOT PROXY<br><br>Falls sich die Person bei der Frage 04911 nicht mehr an das Datum erinnert (TKREB35a =-2, -1)<br>---<br>Ist es... gewesen?	Krebsfrüherkennung Darm<br><br><br>TKREB55	Anal. EHIS 2014<br>*SGB07-SGB12: TKREB36*<br>*SGB17: créé plusieurs questions pour éviter les missings et mieux cibler*

	INT : Antwortmöglichkeiten vorlesen!<br>---<br>1 - In den letzten 12 Monaten<br>2 - Vor 1 bis weniger als 5 Jahre<br>3 - Vor 5 bis weniger als 10 Jahre<br>4 - Vor 10 Jahren oder mehr<br>---<br>(-1) - Weiss nicht<br>(-2) - Keine Antwort		*l'année de dépistage*
04915<br><br>*PROXY*	LINK : IF (04911_b={-1,-2} AND 04911_c={2016}) AND NOT PROXY<br><br>falls das Jahr 2016, aber der Monat unbekannt ist (04911/tkreb35c=2016 et tkreb35b=-2, -1)<br>---<br>Ist es in den letzten 12 Monaten gewesen?<br>---<br>1 - Ja<br>2 - Nein<br>---<br>(-1) - Weiss nicht<br>(-2) - Keine Antwort	Krebsfrüherkennung Darm<br><br>TKREB56	Anal. EHIS 2014<br>*SGB07-SGB12: TKREB36*<br>*SGB17: créé plusieurs questions pour éviter les missings et mieux cibler l'année de dépistage*
04916<br><br>*PROXY*	LINK : IF (04911_b={-1,-2} AND 04911_c={2012}) AND NOT PROXY<br><br>falls das Jahr 2012, aber der Monat unbekannt ist (04911/tkreb35c=2012 et tkreb35b=-2, -1)<br>---<br>Ist es vor weniger als 5 Jahren gewesen?<br>---<br>1 - Ja<br>2 - Nein<br>---<br>(-1) - Weiss nicht<br>(-2) - Keine Antwort	Krebsfrüherkennung Darm<br><br>TKREB57	Anal. EHIS 2014<br>*SGB17: créé plusieurs questions pour éviter les missings et mieux cibler l'année de dépistage*
04917<br><br>*PROXY*	LINK : IF (04911_b={-1,-2} AND 04911_c={2007}) AND NOT PROXY<br><br>falls das Jahr 2007, aber der Monat unbekannt ist (04911/tkreb35c=2007 et tkreb35b=-2, -1)<br>---<br>Ist es vor weniger als 10 Jahren gewesen?<br>---<br>1 - Ja<br>2 - Nein	Krebsfrüherkennung Darm<br><br>TKREB58	Anal. EHIS 2014<br>*SGB17: créé plusieurs questions pour éviter les missings et mieux cibler l'année de dépistage*

	---<br>(-1) - Weiss nicht<br>(-2) - Keine Antwort		
04913<br><br>*PROXY*	LINK : IF 04910={1} AND NOT PROXY<br>---<br>Was ist beim letzten Mal der Grund dafür gewesen, dass Sie diese Darmspiegelung gemacht haben? Ist das gewesen:<br><br>*INT : Antwortmöglichkeiten vorlesen!*<br>---<br>1 - Als Vorsorgeuntersuchung oder "Check-up", ohne dass Sie vorher Beschwerden oder Symptome gehabt hätten<br>2 - Zur Abklärung von Beschwerden oder Symptomen<br>3 - Zur Nachkontrolle, weil eine frühere Untersuchung etwas angezeigt hat<br>4 - Anderer Grund<br>---<br>(-1) - Weiss nicht<br>(-2) - Keine Antwort	Krebsfrüherkennung Darm<br><br>TKREB37	ISPM BE (M. Zwahlen)
05000<br><br>*PROXY*	LINK : ALL AND NOT PROXY<br><br>Wieder an alle<br>---<br>Haben Sie sich schon einmal von einem Arzt vorsorglich Ihre Haut oder Muttermale untersuchen lassen?<br>---<br>1 - Ja<br>2 - Nein **→ 051.00**<br>---<br>(-1) - Weiss nicht **→ 051.00**<br>(-2) - Keine Antwort **→ 051.00**	Krebsfrüherkennung Haut<br><br>TKREB18	BFS - SGB
05001<br><br>*PROXY*	LINK : IF 05000={1} AND NOT PROXY<br>---<br>Wann ist diese Untersuchung von der Haut oder von den Muttermalen bei Ihnen zum letzten Mal durchgeführt worden?<br><br><xx> Monat (TKREB19b)<br><xxxx> Jahr (TKREB19c)<br>---<br>(-1) - Weiss nicht<br>(-2) - Keine Antwort	Krebsfrüherkennung Haut<br><br>TKREB19b<br>TKREB19c	BFS - SGB
05002<br><br>*PROXY*	LINK : IF ((05001_b={-1,-2} AND 05001_c={2016}) OR (05001_b={-1,-2} AND 05001_c={-1,-2})) AND NOT PROXY<br><br>Falls sich die Person bei der Frage 05001 nicht mehr an das Datum erinnert (TKREB19a =-2, -1) oder falls das Jahr 2016 aber der Monat unbekannt ist (TKREB19c =2016 und TKREB19b =-2, -1)	Krebsfrüherkennung Haut	BFS - SGB<br>*um „Missings" zu verhindern*

	--<br>Ist es in den letzten 12 Monaten gewesen?<br>--<br>1 - Ja<br>2 - Nein<br>--<br>(-1) - Weiss nicht<br>(-2) - Keine Antwort	TKREB26	
05100	LINK : IF ALTER>=60<br><br>An alle 60-jährigen und älteren Personen. Personen, die jünger als 60 Jahre sind, weiter zur Frage 52.00<br>--<br>Sind Sie in den letzten 12 Monaten einmal gestürzt?<br>--<br>1 - Ja<br>2 - Nein → **052.00**<br>--<br>(-1) - Weiss nicht → **052.00**<br>(-2) - Keine Antwort → **052.00**	Altersprobleme / Stürze<br><br>TALTP01	NCHS Suppl. aging
05101	LINK : IF 05100={1}<br>--<br>Wie häufig?<br>--<br>1 - 1 Mal<br>2 - 2 Mal<br>3 - Mehr als 2 Mal<br>--<br>(-1) - Weiss nicht<br>(-2) - Keine Antwort	Altersprobleme / Stürze<br><br>TALTP02	NCHS Suppl. aging
05102	LINK : IF 05100={1}<br>--<br>Sind Sie wegen Schwindel gestürzt?<br>--<br>1 - Ja<br>2 - Nein<br>--<br>(-2) - Keine Antwort	Altersprobleme / Stürze<br><br>TALTP03	BFS - SGB<br>SGB92 + SGB02 + SGB17

05200	LINK : ALL<br><br>Wieder an alle<br>--<br>Jetzt kommen wir noch zu allgemeinen Statistikfragen wie Arbeit und Ausbildung.<br><br>Haben Sie in der letzten Woche gegen Entlöhnung geschafft - auch wenn es nur für eine Stunde gewesen ist - egal, ob angestellt, selbständig oder als Lehrling?<br><br>*INT : Falls MILITÄRDIENST länger als 12 Wochen, Rekrutenschule, Unter-/Offiziersschule und Abverdienen oder Zivildienst ==> falls kein Berufsmilitär aktuelle Situation neben dem Militär erfassen*<br>--<br>1 - Ja → **052.10**<br>2 - Nein<br>--<br>(-1) - Weiss nicht<br>(-2) - Keine Antwort	Arbeitssituation<br><br><br>TARSI49	BFS Standard - SHAPE 2011<br>(Q 120.10)
05201	LINK : IF 05200={2, -1, -2}<br>--<br>Haben Sie in der letzten Woche unentgeltlich im Familienbetrieb mitgeholfen, auch wenn es nur für EINE STUNDE gewesen ist? Z.B. in der Landwirtschaft, im Geschäft von Familienangehörigen etc.<br><br>*INT : Nicht als Arbeit gilt Geldverdienen im EIGENEN Haushalt, z.B. Student, der gegen Taschengeld das Auto vom Vater gewaschen hat.*<br>--<br>1 - Ja → **052.10**<br>2 - Nein<br>--<br>(-1) - Weiss nicht<br>(-2) - Keine Antwort	Arbeitssituation<br><br><br>TARSI50	BFS Standard - SHAPE 2011<br>(Q 121.00)
05202	LINK : IF 05201={2, -1, -2}<br>--<br>Obwohl Sie nicht geschafft haben, haben Sie in der LETZTEN WOCHE trotzdem einen Job als Angestellter, Selbständiger oder als Lehrling gehabt, oder schaffen (arbeiten) Sie normalerweise im Familienbetrieb mit?<br><br>*INT : Nicht geschafft zum Beispiel wegen FERIEN, KRANKHEIT, MILITÄR, AUSBILDUNG, aber trotzdem einen gültigen Arbeitsvertrag.*<br>--<br>1 - Ja<br>2 - Nein → **052.40**	Arbeitssituation<br><br><br>TARSI51	BFS Standard - SHAPE 2011<br>(Q 122.00)

	---<br>(-1) - Weiss nicht → **052.40**<br>(-2) - Keine Antwort → **052.40**		
05210	LINK : IF 05200={1} OR 05201={1} OR 05202={1}<br><br>An Personen, die in der letzten Woche gearbeitet haben (TARSI49/52.00=1, oder TARSI50/52.01= 1) oder die eine Erwerbstätigkeit inne hatten aber die letzte Woche nicht gearbeitet haben (TARSI51/52.02=1)<br>---<br>Nebst der ERWERBSTÄTIGKEIT oder dem Job, wo Sie letzte Woche gehabt haben, welche von den folgenden Situationen trifft auf Sie zu? Sind Sie ....<br><br>*INT : ALLES VORLESEN! MEHRERE ANTWORTEN MÖGLICH.*<br>*- Entlöhnte Lehrlinge/Praktikanten/Au-Pair sind NICHT in AUSBILDUNG ;*<br>*- Allein stehende Personen müssen NICHT Hausfrau/-mann angeben*<br>*- Personen, die eine RENTE erhalten (AHV/IV, Pensionskasse) müssen als RENTEN-EMPFÄNGER aufgenommen werden*<br>---<br>21 - Als Arbeitslos eingeschrieben<br>22 - Nicht eingeschrieben, aber auf Stellensuche<br>32 - In Ausbildung (z.B. Student, Schüler)<br>33 - Hausfrau, Hausmann<br>34 - Rentenempfänger<br>35 - Invalid<br>37 - Anderes<br>00 - NEIN, keine weiteren Tätigkeiten	Arbeitssituation<br><br><br>TARSI52a<br>TARSI52b<br>TARSI52c<br>[TARSI52d]	BFS Standard - SHAPE 2011 (Q 123.00)
05211	LINK : IF 05200={1} OR 05201={1} OR 05202={1}<br>---<br>Welche Art von ERWERBSTÄTIGKEIT haben Sie in der letzten Woche gehabt? Ist das eine ...<br><br>*INT : Antwortmöglichkeiten vorlesen!*<br>*- Bei Arbeitnehmern gemäss Arbeitsvertrag*<br>*- Bei Selbstständigerwerbenden: Mehrere Kunden=EINE Stelle/Job*<br>*- Lehrlinge als VOLLZEIT - Erwerbstätige aufnehmen*<br>---<br>1 - Vollzeiterwerbstätigkeit → **052.15**<br>2 - Teilzeiterwerbstätigkeit<br>3 - Mehr als eine Teilzeiterwerbstätigkeit → **052.12**<br>---<br>(-1) - Weiss nicht → **052.15**<br>(-2) - Keine Antwort → **052.15**	Arbeitssituation<br><br><br>TARSI53	BFS Standard - SHAPE 2011 (Q 127.00)

05203	LINK : IF 05211={2}<br><br>Falls eine Teilzeitstelle (TARSI53/52.11=2)<br>---<br>Wieviel Prozent von einer Vollzeitstelle entspricht Ihre Teilzeitstelle?<br><br>- Teilzeit entspricht _ _ % (1 - 90%)<br><br>*INT : Bei Selbstständigerwerbenden oder kleinen Teilzeitpensen beim SCHÄTZEN helfen!!*<br>---<br>(-1) - Weiss nicht<br>(-2) - Keine Antwort	Arbeitssituation<br><br><br>TARSI54a	BFS Standard ab 2012 - SHAPE 2011 (Q 128.00/01)
05212	LINK : IF 05211={3}<br><br>Falls mehr als eine Teilzeitstelle (TARSI53/52.11=3)<br>---<br>Wieviel Prozent von einer Vollzeitstelle entspricht Ihre WICHTIGSTE Teilzeitstelle?<br><br>- Teilzeit entspricht _ _ % (1 - 90%)<br><br>*INT : Bei Selbstständigerwerbenden oder kleinen Teilzeitpensen beim SCHÄTZEN helfen!!*<br>---<br>(-1) - Weiss nicht<br>(-2) - Keine Antwort	Arbeitssituation<br><br><br>TARSI54b	BFS Standard ab 2012 - SHAPE 2011 (Q 128.00/01)
05213	LINK : IF 05211={3}<br><br>Nur falls mehr als eine Teilzeitstelle (TARSI53/52.11=3), falls nur eine Teilzeitstelle (TARSI53/52.11=2) weiter zu 52.15<br>---<br>Wieviel Prozent von einer Vollzeitstelle entspricht Ihre ZWEITWICHTIGSTE Teilzeitstelle?<br><br>- Teilzeit entspricht _ _ % (1 - 90%)<br><br>*INT : Bei Selbstständigerwerbenden oder kleinen Teilzeitpensen beim SCHÄTZEN helfen!!*<br>---<br>(-1) - Weiss nicht<br>(-2) - Keine Antwort	Arbeitssituation<br><br><br>TARSI54c	BFS Standard ab 2012 - SHAPE 2011 (Q 128.02)
05215<br><br><br>*PROXY*	LINK : IF 05211={1,2,-1,-2} AND NOT PROXY<br><br>An alle Personen, die eine Stelle haben (TARSI49/ 52.00=1 oder TARSI50/52.01=1 oder TARSI51/52.02=1). Personen, die mehr als eine Teilzeitstelle haben, weiter zur Frage 52.16	Arbeitssituation<br><br><br>TARSI84a	EWCS Q18 - 10280

	---<br>Wieviele Stunden pro Woche schaffen (arbeiten) Sie normalerweise bei Ihrer Arbeit?<br><br>- Anzahl Stunden pro Woche (3-stellig, von 1 bis 100) _ _ _<br><br>*INT : inkl. Überstunden, aber ohne Pause für Mittagessen (Abendessen), Arbeitsweg. Ab 30 Minuten auf 1 Stunde aufrunden.*<br>---<br>(-1) - Weiss nicht → **052.18**<br>(-2) - Keine Antwort → **052.18**		
05216<br><br>*PROXY*	LINK : IF 05211={3} AND NOT PROXY<br><br>An Personen, die mehr als eine Teilzeitstelle haben (TARSI53/52.11=3). Die anderen weiter zur Frage 52.18<br>---<br>Wieviele Stunden pro Woche schaffen (arbeiten) Sie normalerweise für Ihre Hauptstelle?<br><br>- Anzahl Stunden pro Woche (2-stellig, von 1 bis 90) _ _ _<br><br>*INT : inkl. Überstunden, aber ohne Pause für Mittagessen (Abendessen), Arbeitsweg. Ab 30 Minuten auf 1 Stunde aufrunden.*<br>---<br>(-1) - Weiss nicht<br>(-2) - Keine Antwort	Arbeitssituation<br><br>TARSI84b	EWCS Q18 - 10280
05217<br><br>*PROXY*	LINK : IF 05211={3} AND NOT PROXY<br>---<br>Wie viele Stunden pro Woche schaffen (arbeiten) Sie normalerweise für Ihre zweitwichtigste Stelle?<br><br>- Anzahl Stunden pro Woche (2-stellig, von 1 bis 90) _ _ _<br><br>*INT : inkl. Überstunden, aber ohne Pause für Mittagessen (Abendessen), Arbeitsweg. Ab 30 Minuten auf 1 Stunde aufrunden*<br>---<br>(-1) - Weiss nicht<br>(-2) - Keine Antwort	Arbeitssituation<br><br>TARSI84c	EWCS Q18 - 10280
05218<br><br>*PROXY*	LINK : IF 05200={1} OR 05201={1} OR 05202={1} AND NOT PROXY<br><br>Wieder an alle Personen, die erwerbstätig sind (TARSI49/52.00=1, oder TARSI50/52.01= 1, oder TARSI51/52.02= 1)<br>---<br>Wie häufig im Monat schaffen (arbeiten) Sie normalerweise mehr als 10 Stunden pro Tag?<br><br><xx> - Tage pro Monat<br>---<br>0 - Nie	Arbeitssituation<br><br>TARSI82	EWCS Q14E

	---<br>(-1) - Weiss nicht<br>(-2) - Keine Antwort		
05219<br><br>*PROXY*	LINK : IF 05200={1} OR 05201={1} OR 05202={1} AND NOT PROXY<br>---<br>Wie häufig im Monat schaffen (arbeiten) Sie normalerweise in der Nacht, also mindestens 2 Stunden zwischen 11 Uhr in der Nacht und 6 Uhr am Morgen?<br><br><xx> - Anzahl Nächte pro Monat (max. 30)<br>---<br>0 - Nie<br>---<br>(-1) - Weiss nicht<br>(-2) - Keine Antwort	Arbeitssituation<br><br>TARSI56	EWCS Q32 - 10500
05220	LINK : IF 05200={1} OR 05201={1} OR 05202={1}<br>---<br>Schaffen (arbeiten) Sie in wechselnden/rotierenden Schichten, z.B. früh, spät, Nacht?<br><br>*INT : Antwortmöglichkeiten vorlesen!*<br>---<br>1 - Nie<br>2 - Manchmal<br>3 - Häufig<br>4 - Immer<br>---<br>(-2) - Keine Antwort	Arbeitssituation<br><br>TARSI57	SIP 2010, E13
05221	LINK : IF 05200={1} OR 05201={1} OR 05202={1}<br>---<br>Haben Sie unregelmässige Arbeitszeiten, wo schwer vorhersehbar sind, z.B. Arbeit auf Abruf?<br><br>*INT : Antwortmöglichkeiten vorlesen!*<br>---<br>1 - Nie<br>2 - Manchmal<br>3 - Häufig<br>4 - Immer<br>---<br>(-2) - Keine Antwort	Arbeitssituation<br><br>TARSI58	SIP 2010, E13 modifiziert
05233	LINK : IF 05200={1} OR 05201={1} OR 05202={1}	Arbeitssituation	EHIS 2014

	--<br>Wenn Sie schaffen (arbeiten), wie machen Sie dies hauptsächlich?<br><br>*INT : Antwortmöglichkeiten vorlesen!*<br>--<br>1 - Meistens im Sitzen (ohne jegliche körperliche Anstrengung)<br>2 - Meistens im Stehen (ohne körperliche Anstrengung)<br>3 - Meistens im Gehen bzw eine mässig anstrengende körperliche Tätigkeit<br>4 - Meistens schwere körperliche Arbeit oder körperlich anstrengende Tätigkeiten<br>--<br>(-1) - Weiss nicht<br>(-2) - Keine Antwort	TARSI85	
05234	LINK : IF 05200={1} OR 05201={1} OR 05202={1}<br>--<br>Während wievielen Tagen oder Halbtagen haben Sie in den letzten vier Wochen Ihre berufliche Tätigkeit aufgrund von Krankheit oder Unfall nicht können ausüben?<br><br><xx> Anzahl Tage (TZWBH06a) (max. 30)<br><xx> Anzahl halbe Tage (TZWBH06b) (max. 30)<br><br>*INT : Alle Stellen zusammengezählt! Anzahl (halbe) Tage, die man von der Arbeit fernbleibt. NICHT Anzahl (halbe) Tage, die man krank war.*<br>--<br>(-1) - Weiss nicht<br>(-2) - Keine Antwort	Arbeitssituation<br><br>TZWBH06a<br>TZWBH06b	BFS - SAKE<br>*SGB17: halb-tag eingefügt*
05223	LINK : IF 05200={1} OR 05201={1} OR 05202={1}<br>--<br>Die folgenden Fragen beziehen sich nur auf Ihre berufliche HAUPTTÄTIGKEIT. Bei mehreren Stellen beziehen sich die Fragen auf die wichtigste Stelle.<br><br>Sind Sie in Ihrer JETZIGEN beruflichen Tätigkeit ...<br><br>*INT : Antwortmöglichkeiten vorlesen!*<br>*=> Wichtigste Stelle = in der Regel Stelle mit den meisten Arbeitsstunden.*<br>*=> Selbstständigerwerbende: Mehrere Kunden = Eine Stelle/Job*<br>*=> Angestellt von einem Privathaushalt (Putzfrau/Babysitting..)= Arbeitnehmer bei einem privaten Unternehmen = (4)*<br>--<br>1 - Selbständig Erwerbende(r)<br>2 - Arbeitnehmer(in) in der AG oder GmbH, wo Ihnen selbst gehört<br>3 - Arbeitnehmer(in) im Familienbetrieb von einem Haushaltsmitglied<br>4 - Arbeitnehmer(in) bei einem sonstigen privaten oder öffentlichen Unternehmen **→ 052.26**<br>5 - Lehrling **→ 052.32**	Arbeitssituation<br><br>TARSI60	BFS Standard ab 2012 - SHAPE 2011 (Q 130.00)

05224	LINK : IF 05223={1,2,3}<br><br>Nur an selbständig Erwerbende, Angestellte im eigenen Unternehmen oder Mitarbeitende im Familienbetrieb (TARSI60/52.23= 1, 2, 3)<br>---<br>Haben Sie Mitarbeiter, wo Ihnen direkt oder indirekt unterstellt sind?<br>---<br>1 - Ja<br>2 - Nein → **052.32**<br>---<br>(-1) - Weiss nicht → **052.32**<br>(-2) - Keine Antwort → **052.32**	Arbeitssituation<br><br><br>TARSI61	BFS Standard - SHAPE 2011 (Q 133.00)
05225<br><br><br>*PROXY*	LINK : IF 05224={1} AND NOT PROXY<br>---<br>Wie viele?<br><br><xxx> - Angestellte (1 bis 899)<br><997> - 900 Angestellte und mehr<br>---<br>(-1) - Weiss nicht<br>(-2) - Keine Antwort	Arbeitssituation<br><br><br>TARSI62	BFS – SGB
05226	LINK : IF 05223={4}<br><br>Nur an Arbeitnehmer bei einem privaten oder öffentlichen Unternehmen (TARSI60/52.23=4).<br>Angestellte im eigenen Unternehmen, Mitarbeitende im Familienbetrieb, Lehrlinge oder selbständig Erwerbende weiter zur Frage 52.32<br>---<br>Welche Position haben Sie in Ihrer jetzigen beruflichen Haupttätigkeit? Sind Sie...<br><br>*INT : Antwortmöglichkeiten vorlesen!*<br>---<br>1 - Angestellt ohne Cheffunktion (keine direkt oder indirekt unterstellten Mitarbeiter!)<br>2 - Angestellt mit Chefposition und unterstellten Mitarbeitern (Bürochef, Filialleiter, Gruppenchef, mittleres Kader)<br>3 - Angestellt als Mitglied von der Direktion oder Geschäftsleitung (Direktor, Direktorin, Mitglied der Unternehmensleitung)<br>---<br>(-1) - Weiss nicht<br>(-2) - Keine Antwort	Arbeitssituation<br><br><br>TARSI63	BFS Standard - SHAPE 2011 (Q 132.00)
05232	LINK : IF 05200={1} OR 05201={1} OR 05202={1}<br><br>Wieder an alle Personen, die arbeiten (TARSI49/52.00=1, TARSI50/52.01= 1, TARSI51/52.02= 1)<br>---<br>Beeinflusst Ihre Arbeit Ihre Gesundheit?	Arbeitssituation<br><br><br>TARSI86	EWCS Q67

	INT : Antwortmöglichkeiten vorlesen!<br>---<br>1 - Ja, hauptsächlich positiv<br>2 - Ja, hauptsächlich negativ<br>3 - Nein<br>---<br>(-1) - Weiss nicht<br>(-2) - Keine Antwort		
05228	LINK : IF 05200={1} OR 05201={1} OR 05202={1}<br><br>An Personen, die arbeiten (TARSI49/52.00=1, TARSI50/52.01= 1, TARSI51/52.02= 1)<br>---<br>Was ist Ihr Beruf in Ihrer JETZIGEN beruflichen Haupttätigkeit?<br>Was genau machen Sie?<br><br>Lehrling: Was lernen Sie für einen Beruf?<br><br>STAMM_OFS :<br><xxxxxxxx> Berufsliste BFS<br><br>*INT : Z.B:*<br>*- Geografielehrer und nicht einfach Geograf*<br>*- Chefbuchhalter und nicht einfach Chef*<br>*- Bankangestellte und nicht einfach kaufmännische Angestellte*<br>---<br>91 - Automatische Suche in der Berufsliste<br>90 - Manuelle Berufseingabe<br>---<br>(-1) - Weiss nicht<br>(-2) - Keine Antwort	Arbeitssituation<br><br><br>[STAMM_OFS_TYP]<br>[STAMM_OFS]<br>CH_ISCO_19<br>CH_ISCO_19_text_d<br>CH_ISCO_19_text_f<br>CH_ISCO_19_text_i	BFS Standard ab 2012 - SHAPE 2011 (Q 134.00 /135.00)
05229	LINK : IF 05200={1} OR 05201={1} OR 05202={1}<br>---<br>Wie ist der NAME von Ihrem Betrieb und in welcher ORTSCHAFT befindet er sich? Wenn sich's um einen Betrieb mit mehreren Filialen handelt, so sagen Sie mir die Ortschaft, wo das GEBÄUDE steht, wo Sie drin schaffen (arbeiten).<br><br>TARSI65a :<br><xxxxxxxx> n° BUR → 056.00<br>---<br>91 - Automatische Suche = in der Betriebsliste/BUR **→ 056.00**<br>87 - Bauernhof, Landwirtschaft **→ 056.00**	Arbeitssituation<br><br><br>[TARSI65]<br>[TARSI65a]<br>noga_2008	BFS Standard - SHAPE 2011 (Q 136.00)

	88 - Arbeitgeber = anderer Privathaushalt → **056.00**<br>89 - Betrieb im Ausland, Grenzgänger<br>90 - Manuelle Namens-u. Adresseingabe<br>---<br>(-1) - Weiss nicht<br>(-2) - Keine Antwort		
05230	LINK : IF 05229={89,90,-1,-2}<br>---<br>Angestellte: In welcher Wirtschaftsbranche ist Ihr Betrieb genau tätig?<br>Selbständig Erwerbende: In welcher Wirtschaftsbranche sind Sie genau tätig?<br><br>*INT : Ingenieur-/Architektur-/Geometerbüro = Code (07)*<br>---<br>1 - Land-/Forstwirtschaft/Gartenbau/Tierhaltung/Fischerei → **056.00**<br>2 - Rohstoffgewinnung (Steinkohlen, Salz,...) → **056.00**<br>3 - Industrie/Herstellung von Waren (Nahrungsmittel, Maschinen oder andere Artikel) → **056.00**<br>4 - Erzeugung und Versorgung von Strom/Gas/Kältetechnik → **056.00**<br>5 - Wasserversorgung, Abfallentsorgung → **056.00**<br>6 - Baugewerbe/Ausbaugewerbe (Wohnung, Sanitäranlagen, Malerei, Tiefbauten,...) → **056.00**<br>7 - Dienstleistungsbranche<br>---<br>(-1) - Weiss nicht → **056.00**<br>(-2) - Keine Antwort → **056.00**	Arbeitssituation<br><br><br>[TARSI66]<br>noga_2008	NOGA-Nomenklatur, BFS 2002<br>BFS-Standard ab 2012 - SHAPE 2011 (Q 138.00 /138.10)
05231	LINK : IF 05230={7}<br>---<br>Angestellte: In welcher Dienstleistungsbranche ist Ihr Betrieb oder Ihre Filiale tätig? Selbständig Erwerbende: In welcher Dienstleistungsbranche sind Sie tätig?<br>---<br>8 - Handel, Reparatur Motorfahrzeug<br>9 - Verkehr, Lagerei, Post<br>10 - Gastgewerbe, Gastronomie<br>11 - Information und Kommunikation (Zeitungen, Tel., Informatik)<br>12 - Banken, Versicherungen<br>13 - Immobilien<br>14 - Recht., Wirtschaftsprüf., Werbung Wissenschaft (Ing., F&E, Architekt)<br>15 - Vermietung, Reisebüros, Vermittlung<br>16 - Öffentliche Verwaltung<br>17 - Unterrichtswesen<br>18 - Gesundheits- u. Sozialwesen<br>19 - Kunst, Unterhaltung, Sport, Museen, Bibliotheken, Casinos<br>20 - Sonst. Dienstl., Reparatur	Arbeitssituation<br><br><br>[TARSI67]<br>noga_2008	BFS Standard - SHAPE 2011 (Q 139.00/10)

	21 - Private Haushalte<br>22 - Botschaft, internationale Organisationen<br>--<br>(-1) - Weiss nicht<br>(-2) - Keine Antwort		
05240	LINK : IF 05200={2,-1,-2} AND 05201={2,-1,-2} AND 05202={2,-1,-2} AND ALTER <=69<br><br>Nur an Personen, die in der letzten Woche nicht gearbeitet haben (+w.n. / +k.A.) (TARSI49/52.00=2,-1,-2 oder TARSI50/52.01=2,-1,-2 oder TARSI51/52.02=2,-1,-2); Die anderen weiter zu Frage 56.00<br>--<br>Haben Sie in den letzten vier Wochen eine Arbeit gesucht?<br>--<br>1 - Ja<br>2 - Nein → **052.42**<br>--<br>(-1) - Weiss nicht → **052.42**<br>(-2) - Keine Antwort → **052.42**	Arbeitssituation<br><br><br>TARSI69	BFS Standard - SHAPE 2011 (Q 125.00)
05241<br><br><br>*PROXY*	LINK : IF 05240={1} AND NOT PROXY<br>--<br>Wann könnten Sie von sich aus frühestens anfangen?<br>--<br>1 - Sofort<br>2 - Innerhalb der nächsten 2 Wochen<br>3 - In 3 bis 4 Wochen<br>4 - In 5 Wochen bis 3 Monaten<br>5 - Später als in 3 Monaten<br>6 - Ist nicht verfügbar<br>7 - Hat bereits Job gefunden, beginnt später<br>--<br>(-1) - Weiss nicht<br>(-2) - Keine Antwort	Arbeitssituation<br><br><br>TARSI70	BFS Standard - SHAPE 2011 (Q 126.00)
05242	LINK : IF 05200={2,-1,-2} AND 05201={2,-1,-2} AND 05202={2,-1,-2}<br>--<br>Welche von den folgenden Situationen trifft auf Sie zu? Sind Sie ...<br><br>*INT : ALLES VORLESEN! MEHRERE ANTWORTEN MÖGLICH.*<br>*- Entlöhnte Lehrlinge/Praktikanten/Au-Pair sind NICHT in AUSBILDUNG ;*<br>*- Allein stehende Personen müssen NICHT Hausfrau/-mann angeben*<br>*- Personen, die eine RENTE erhalten (AHV/IV, Pensionskasse) müssen als RENTEN-EMPFÄNGER aufgenommen werden*	Arbeitssituation<br><br><br>TARSI71a<br>TARSI71b<br>TARSI71c<br>TARSI71d<br>TARSI71e	BFS Standard - SHAPE 2011 (Q 124.00)

	--<br>21 - Als Arbeitslos eingeschrieben<br>22 - Nicht eingeschrieben, aber auf Stellensuche<br>32 - In Ausbildung (z.B. Student, Schüler)<br>33 - Hausfrau, Hausmann<br>34 - Rentenempfänger<br>35 - Invalid<br>36 - Im Militär-, Zivildienst<br>37 - Anderes		
05600	LINK : ALL<br>--<br>Jetzt möchten ich Ihnen gerne ein paar Fragen zu Ihren abgeschlossenen Ausbildungen stellen. Welche Schule oder Ausbildung haben Sie nach der obligatorischen Schule als ERSTES ABGESCHLOSSEN?<br><br>*INT : "abgeschlossen" d.h. mit Zeugnis, Fähigkeitsausweis oder Diplom*<br>--<br>1 - Hat keine Schule besucht **→ 053.00**<br>2 - Hat die obligatorische Schule nicht abgeschlossen **→ 056.01**<br>3 - Hat nur die obligatorische Schule abgeschlossen **→ 056.01**<br>4 - 1-jährige Ausbildung: 10.Schuljahr/Berufswahlschule/Vorlehre/Sprachschule mit Zertifikat/Haushaltslehrjahr/Brückenangebote **→ 056.10**<br>5 - 2-jährige berufliche Grundbildung: eidg. Berufsattest (EBA) ehem. Anlehre 1-2 Jahre) **→ 056.10**<br>6 - 2-jährige Vollzeitberufsschule, Handelsschule **→ 056.10**<br>7 - 2-3 jährige Ausbildung: allgemeinbildende Schule (Diplommittelschule, Fachmittelschule FMS, Verwaltungsschule) **→ 056.13**<br>8 - 2-4 jährige Berufslehre, duale berufliche Grundbildung mit eidg. Fähigkeitszeugnis **→ 056.13**<br>9 - 3-4 jährige Vollzeitberufsschule, Lehrwerkstätte, Handelsmittelschule **→ 056.10**<br>10 - Lehrkräfte-Seminar (ehem. Lehrerseminar) **→ 056.10**<br>11 - Gymnasiale Maturität **→ 056.10**<br>12 - Berufs- oder Fachmaturität **→ 056.10**<br>--<br>(-1) - Weiss nicht **→ 056.10**<br>(-2) - Keine Antwort **→ 056.10**	Soziodemographie<br>Ausbildung<br><br><br>[TSODE86]	Ab 2012 BFS Standard - SHAPE 2011 (Q 110.10)<br>-> *Variable AUSBILD*
05601	LINK : IF 05600={2,3}<br><br>Nur an Personen, welche die obligatorische Schule nicht abgeschlossen oder die nur die obligatorische Schule abgeschlossen haben (TSODE86/56.00=2,3)<br><br>Die Personen mit 56.00/TSODE86=7,8, weiter zu Frage 56.13.<br>Die Personen mit den anderen abgeschlossenen Ausbildungen (56.00/TARSI86=4,5,6,9,10,11,12,-2,-1), weiter zu Frage 56.10.<br>--<br>Haben Sie das achte oder neunte Schuljahr abgeschlossen?	Soziodemographie<br>Ausbildung<br><br><br>[TSODE87]	Ab 2012 BFS Standard - SHAPE 2011 (Q 111.00)<br>-> *Variable AUSBILD*

	--<br>1 - Ja → **053.00**<br>2 - Nein → **053.00**<br>--<br>(-1) - Weiss nicht → **053.00**<br>(-2) - Keine Antwort → **053.00**		
05613	LINK : IF 05600={7, 8}<br><br>Nur an Personen, die allgemeinbildende Schule oder Berufslehre abgeschlossen haben<br>--<br>Wie lange hat diese Schule / Berufsbildung insgesamt gedauert?<br>--<br>1 - Weniger als 3 Jahre → **056.10**<br>2 - 3 Jahre oder mehr → **056.10**<br>--<br>(-1) - Weiss nicht → **056.10**<br>(-2) - Keine Antwort → **056.10**	Soziodemographie<br>Ausbildung<br><br><br>[TSODE86d]	BFS – SGB<br>-> *Variable AUSBILD*
05610	LINK : IF 05600={4,5,6, 9 TO 12, -1, -2}<br><br>Nur an Personen, die eine nachobligatorische Ausbildung absolviert haben (TSODE86/56.00= 4 - 12, -1, -2).<br>Die anderen weiter zu Frage 53.00<br>--<br>Haben Sie nach Abschluss von Ihrer ersten Ausbildung noch eine weitere Ausbildung abgeschlossen?<br><br>*INT : "abgeschlossen" d.h. mit Zeugnis, Fähigkeitsausweis oder Diplom*<br>--<br>1 - Ja<br>2 - Nein → **053.00**<br>--<br>(-1) - Weiss nicht → **053.00**<br>(-2) - Keine Antwort → **053.00**	Soziodemographie<br>Ausbildung<br><br><br>[TSODE88a]	Ab 2012 BFS Standard - SHAPE 2011 (Q 112.00)<br>-> *Variable AUSBILD*
05611	LINK : IF 05610={1}<br>--<br>Um was für eine Ausbildung hat es sich gehandelt?<br>--<br>4 - 1-jährige Ausbildung: 10.Schuljahr/Berufswahlschule/Vorlehre/Sprachschule mit Zertifikat/Haushaltslehrjahr/Brückenangebote → **056.20**<br>5 - 2-jährige berufliche Grundbildung: eidg. Berufsattest (EBA) (ehem. Anlehre 1-2 Jahre) → **056.20**<br>6 - 2-jährige Vollzeitberufsschule, Handelsschule → **056.20**<br>7 - 2-3 jährige Ausbildung: allgemeinbildende Schule (Diplommittelschule, Fachmittelschule FMS, Verwaltungsschule) → **056.14**	Soziodemographie<br>Ausbildung<br><br><br>[TSODE88b]<br>[TSODE89b]<br>[TSODE90b]<br>[TSODE91b]	Ab 2012 BFS Standard - SHAPE 2011 (Q 114.00)<br>-> *Variable AUSBILD*

	8 - 2-4 jährige Berufslehre, duale berufliche Grundbildung mit eidg. Fähigkeitszeugnis → **056.14**<br>9 - 3-4 jährige Vollzeitberufsschule, Lehrwerkstätte, Handelsschule → **056.20**<br>10 - Lehrkräfte-Seminar (ehem. Lehrerseminar) → **056.20**<br>11 - Gymnasiale Maturität → **056.20**<br>12 - Berufs- oder Fachmaturität → **056.20**<br>13 - Höhere Berufsbildung mit eidg. Fachausweis → **056.20**<br>14 - Höhere Berufsbildung mit eidg. Diplom (oder Meisterdiplom) → **056.20**<br>15 - Höhere Fachschule (HF) für Technik (bzw. Technikerschule TS), HF für Wirtschaft (bzw. HKG) (2J. Voll- od. 3J. Teilzeit) → **056.15**<br>16 - Höhere Fachschule HWV, HFG, HFS, Ingenieurschule HTL, (3J. Voll- od. 4J. Teilzeit) → **056.15**<br>17 - Fachhochschule (FH) → **056.16**<br>18 - Pädagogische Hochschule (PH) → **056.16**<br>19 - Universität, ETH → **056.17**<br>---<br>(-1) - Weiss nicht → **053.00**<br>(-2) - Keine Antwort → **053.00**		
05614	LINK : IF 05611={7, 8}<br><br>für 56.11=7, 8<br>---<br>Wie lange hat diese Schule / Berufsbildung insgesamt gedauert?<br>---<br>1 - Weniger als 3 Jahre → **056.20**<br>2 - 3 Jahre oder mehr → **056.20**<br>---<br>(-1) - Weiss nicht → **056.20**<br>(-2) - Keine Antwort → **056.20**	Soziodemographie<br>Ausbildung<br><br><br>[TSODE88d]<br>[TSODE89d]<br>[TSODE90d]<br>[TSODE91d]	BFS – SGB<br>-> *Variable AUSBILD*
05615	LINK : IF 05611={15, 16}<br><br>NUR FUR PERSONEN MIT EINER AUSBILDUNG = HÖHERE FACHSCHULE (HF, HFG, HTL) (056.11=15, 16)<br>---<br>Können Sie mir sagen, wie man diesen Abschluss genau bezeichnet? Ist das ein...<br>---<br>1 - Diplom<br>2 - Nachdiplom<br>---<br>(-1) - Weiss nicht → **053.00**<br>(-2) - Keine Antwort → **053.00**	Soziodemographie<br>Ausbildung<br><br><br>[TSODE88e]<br>[TSODE89e]<br>[TSODE90e]<br>[TSODE91e]	BFS – SGB<br>-> *Variable AUSBILD*
05616	LINK : IF 05611={17, 18}<br><br>NUR FUR PERSONEN MIT EINER AUSBILDUNG = FACHHOCHSCHULE (FH) ODER PÀDAGOGISCHE HOCHSCHULE (PH) (056.11=17, 18)	Soziodemographie<br>Ausbildung	BFS – SGB<br>-> *Variable AUSBILD*

	---<br>Können Sie mir sagen, wie man diesen Abschluss genau bezeichnet? Ist das ein...<br><br>*INT : Nachdiplome in Kategorie 3*<br>---<br>1 - Diplom, Bachelor (2-3 Jahre)<br>2 - Master (4-5 Jahre)<br>3 - Postgrade, Master of Advanced Studies (MAS), Certificate of Advanced Studies (CAS), Diploma of Advanced Studies (DAS)<br>4 - DOKTORAT, Habilitation<br>---<br>(-1) - Weiss nicht **→ 053.00**<br>(-2) - Keine Antwort **→ 053.00**	[TSODE88f]<br>[TSODE89f]<br>[TSODE90f]<br>[TSODE91f]	
05617	LINK : IF 05611={19}<br><br>NUR FUR PERSONEN MIT EINER AUSBILDUNG = UNIVERSITÀT, ETH (056.11=19)<br>---<br>Können Sie mir sagen, wie man diesen Abschluss genau bezeichnet? Ist das ein...<br><br>*INT : Nachdiplome in Kategorie 3*<br>---<br>1 - Bachelor (2-3 Jahre)<br>2 - Master, Diplom, Lizentiat (4-5 Jahre)<br>3 - Postgrade, Master of Advanced Studies (MAS), Certificate of Advanced Studies (CAS), Diploma of Advanced Studies (DAS)<br>4 - DOKTORAT, Habilitation<br>---<br>(-1) - Weiss nicht **→ 053.00**<br>(-2) - Keine Antwort **→ 053.00**	Soziodemographie<br>Ausbildung<br><br><br>[TSODE88g]<br>[TSODE89g]<br>[TSODE90g]<br>[TSODE91g]	BFS – SGB<br>*-> Variable AUSBILD*
05620	LINK : IF (05611={4,5,6, 9 TO 14}) OR (05614={1,2}) OR (05615={1,2}) OR (05616={1 TO 4}) OR (05617={1 TO 4})<br>---<br>Haben Sie nachher noch eine weitere Ausbildung abgeschlossen?<br><br>*INT : "abgeschlossen" d.h. mit Zeugnis, Fähigkeitsausweis oder Diplom*<br>---<br>1 - Ja<br>2 - Nein **→ 053.00**<br>---<br>(-1) - Weiss nicht **→ 053.00**<br>(-2) - Keine Antwort **→ 053.00**	Soziodemographie<br>Ausbildung<br><br><br>[TSODE89a]<br>TSODE90a<br>[TSODE91a]	Ab 2012 BFS Standard - SHAPE 2011 (Q113.00)<br>*Die Personen können bis zu 4 Ausbildungen angeben, wobei der Frageblock als Schlaufe abläuft (56.20,56.21, 56.22)*<br>*-> Variable AUSBILD*
05630	Höchste abgeschlossene Ausbildung	Soziodemographie<br>Ausbildung	Ab 2012 BFS Standard - SHAPE 2011 (Q113.00)

	---<br>1 - Hat keine Schule besucht<br>2 - Hat die obligatorische Schule nicht abgeschlossen<br>3 - Hat nur die obligatorische Schule abgeschlossen<br>4 - 1-jährige Ausbildung: 10.Schuljahr/Berufswahlschule/Vorlehre/Sprachschule mit Zertifikat/Haushaltslehrjahr/Brückenangebote<br>5 - 2-jährige berufliche Grundbildung: eidg. Berufsattest (EBA) ehem. Anlehre 1-2 Jahre)<br>6 - 2-jährige Vollzeitberufsschule, Handelsschule<br>7 - 2-3 jährige Ausbildung: allgemeinbildende Schule (Diplommittelschule, Fachmittelschule FMS, Verwaltungsschule)<br>8 - 2-4 jährige Berufslehre, duale berufliche Grundbildung mit eidg. Fähigkeitszeugnis<br>9 - 3-4 jährige Vollzeitberufsschule, Lehrwerkstätte, Handelsmittelschule<br>10 - Lehrkräfte-Seminar (ehem. Lehrerseminar)<br>11 - Gymnasiale Maturität<br>12 - Berufs- oder Fachmaturität<br>13 - Höhere Berufsbildung mit eidg. Fachausweis<br>14 - Höhere Berufsbildung mit eidg. Diplom (oder Meisterdiplom)<br>15 - Höhere Fachschule (HF) für Technik (bzw. Technikerschule TS), HF für Wirtschaft (bzw. HKG) (2J. Voll- od. 3J. Teilzeit)<br>16 - Höhere Fachschule HWV, HFG, HFS, Ingenieurschule HTL, (3J. Voll- od. 4J. Teilzeit)<br>17 - Fachhochschule (FH)<br>18 - Pädagogische Hochschule (PH)<br>19 - Universität, ETH<br>20 - FH, PH, Universität, ETH mit Doktorat<br>---<br>(-1) - Weiss nicht<br>(-2) - Keine Antwort	AUSBILD	*Construction de la variable AUSBILD avec les variables TSODE86 à TSODE91a-g*
i5300	Jetzt einige Fragen zu Ihrem Haushalt und zu Ihrer Herkunft.<br><br>Um das Interview zu vereinfachen, verwendet das Bundesamt für Statistik gewisse Informationen aus dem Bevölkerungsregister. Weil es aber sein kann, dass diese Daten ungenau oder nicht mehr ganz aktuell sind, möchten wir diese Daten von Ihnen bestätigen lassen.<br>---<br>1 - OK, weiter<br>2 - Verweigerung **→ 053.40**	i5300	
05300	LINK : NOT ASKED<br><br>Wieder an Alle:<br>---<br>ZIVILSTAND : Information aus dem Einwohnerregister<br>---<br>1 - Ledig<br>2 - Verheiratet<br>3 - Verwitwet<br>4 - Geschieden<br>5 - Unverheiratet	Soziodemographie Zivilstand<br><br>MARITALSTATUS	Ab 2012 BFS Standard - SHAPE 2011 (Q 102.00)

	6 - In eingetragener Partnerschaft<br>7 - Aufgelöste Partnerschaft<br>---<br>(-2) - Keine Antwort		
05311	LINK : IF i5300={1} and NBPERS_REG>=2<br><br>Für Haushalte mit mindestens 2 Personen (NBPERS_REG>=2).<br>1 Personen-Haushalte weiter zu Frage 05312<br>---<br>Ich lese Ihnen zuerst die Vornamen von allen Personen vor, wo gemäss Bevölkerungsregister zu Ihrem Haushalt gehören. Bitte antworten Sie für jede von den folgenden Personen mit JA oder NEIN, ob sie mindestens 4 Tage pro Woche mit Ihnen zusammen lebt.<br><br>*INT : Personen, die nur 2 oder 3 Tage pro Woche oder ferienhalber im befragten Haushalt anwesend sind, werden nicht mitgezählt.*<br><br>*VORNAMEN einzeln vorlesen:*<br><br>*PGM : CF. VARIABLES DES REGISTRES (FIRSTNAME, SEX, DATEOFBIRTH)*<br><br>*X1 Vorname, (Geschlecht, Alter)*<br>*X2 Vorname, (Geschlecht, Alter)*<br>*X3 Vorname, (Geschlecht, Alter)*<br>*Usw.*<br>---<br>1 - Ja, wohnt hier<br>2 - Nein, wohnt nicht hier	Soziodemografie<br>Haushaltstruktur<br><br><br>[TCONF_P01-P13]	BFS - SGB<br>*-> Variable HHTYP*
05312	LINK : IF i5300={1} and NBPERS_REG>=2<br>---<br>Leben noch andere Personen mindestens 4 Tage pro Woche in Ihrem Haushalt, wo ich nicht vorgelesen habe?<br><br>*INT : Liste der Personen im Haushalt.*<br>---<br>1 - Ja<br>2 - Nein **→ 053.30**<br>---<br>(-1) - Weiss nicht **→ 053.30**<br>(-2) - Keine Antwort **→ 053.30**	Soziodemografie<br>Haushaltstruktur<br><br><br>[TSODE100a]	BFS - SGB<br>*-> Variable HHTYP*
05313	LINK : IF i5300={1} and NBPERS_REG=1<br>---<br>Gemäss Bevölkerungsregister leben Sie alleine. Leben gleich noch andere Personen mindestens 4 Tage pro Woche in Ihrem Haushalt?	Soziodemografie<br>Haushaltstruktur<br><br><br>[TSODE100b]	BFS - SGB<br>*-> Variable HHTYP*

	--<br>1 - Ja<br>2 - Nein → **053.30**<br>--<br>(-1) - Weiss nicht → **053.30**<br>(-2) - Keine Antwort → **053.30**		
05314	LINK : IF 05312={1} OR 05313={1}<br>--<br>Sagen Sie mir bitte den Vornamen, das Geschlecht und das jetzige Alter in vollendeten Altersjahren von all diesen zusätzlichen Personen. Fangen Sie bitte mit der ältesten Person an.<br><br>*INT : Jetziges Alter 2stellig eingeben! Bitte NICHT den Jahrgang.*<br><br>*Y1 Vorname, Geschlecht, Alter*<br>*Y2 Vorname, Geschlecht, Alter*<br>*Y3 Vorname, Geschlecht, Alter*<br>*Etc*<br>--<br>(-1) - Weiss nicht → **053.30**<br>(-2) - Keine Antwort → **053.30**	Soziodemografie<br>Haushaltstruktur<br><br><br>ALTER_P1-P14<br>SEX_P1-P14<br>NBKIDS14<br>NBKIDS17	BFS - SGB<br>-> *Variable HHTYP*
05350	Haushaltstyp<br>--<br>11 - Einpersonenhaushalt<br>21 - Paare ohne Kinder<br>22 - Paare mit Kind(ern)<br>23 - Einelternhaushalte mit Kind(ern)<br>3 - Mehrfamilienhaushalt<br>12 - Nichtfamilienhaushalt mit mehreren Personen<br>--<br>(-1) - Weiss nicht<br>(-2) - Keine Antwort	Soziodemographie<br>Haushaltstyp<br><br><br>HHTYP	Ab 2012 BFS Standard - SHAPE 2011(Q 150.10)<br>*Construction de la variable HHTYP avec les questions 05311 à 05330*
05330	LINK : IF NBPERS_b>={2}<br><br>Für Haushalte mit mindestens 2 Personen (NBPERS_b>=2).<br>1 Personen-Haushalte weiter zu Frage 05340<br>--<br>Jetzt geht es darum in welchem Verwandtschaftsverhältnis die Personen in Ihrem Haushalt zu Ihnen stehen.<br>Wie steht X zu Ihnen? Ist er/sie :...<br>(Wenn Person X = weiblich):<br><br><0> Zielperson	Soziodemographie<br><br><br>VERW_P1-P14	Ab 2012 BFS Standard - SHAPE 2011 (Q 151.00 / 152.00)<br>-> *Variable HHTYP*

	<1> Ehefrau<br><2> Lebenspartnerin<br><3> Tochter<br><4> Stieftochter (Tochter von Partner-in)<br><5> Schwiegertochter (Partnerin des Sohns)<br><6> Mutter<br><7> Schwiegermutter (Mutter von Partner-in)<br><8> Stiefmutter (Lebenspartnerin des Vaters / der Mutter)<br><9> Schwester oder Halbschwester<br><10> Schwägerin<br><11> Enkelin<br><12> Grossmutter<br><13> Tante, Nichte, Cousine<br><14> andere verwandtschaftliche Beziehung<br><20> keine verwandtschaftliche Beziehung<br><br>(Wenn Person X = männlich):<br><0> Zielperson<br><1> Ehemann<br><2> Lebenspartner<br><3> Sohn<br><4> Stiefsohn (Sohn von Partner-in)<br><5> Schwiegersohn (Partner der Tochter)<br><6> Vater<br><7> Schwiegervater (Vater von Partner-in)<br><8> Stiefvater (Lebenspartner der Mutter / des Vaters)<br><9> Bruder oder Halbbruder<br><10> Schwager<br><11> Enkel<br><12> Grossvater<br><13> Onkel, Neffe, Cousin<br><14> andere verwandtschaftliche Beziehung<br><20> keine verwandtschaftliche Beziehung<br>---<br>0 - Zielperson<br>1 - Ehefrau, -mann<br>2 - Lebenspartner/in<br>3 - Tochter, Sohn<br>4 - Stieftochter, -sohn (Tochter, Sohn von Partner-in)<br>5 - Schwiegertochter, -sohn (Partnerin des Sohns, Partner der Tochter)<br>6 - Mutter, Vater<br>7 - Schwiegermutter, -vater (Mutter, Vater von Partner-in)<br>8 - Stiefmutter- Vater (Lebenspartner/in Vater, Mutter)		

	9 - Schwester, Halbschwester, Bruder, Halbbruder<br>10 - Schwäger/in<br>11 - Enkel/in<br>12 - Grossmutter, -vater<br>13 - Tante, Nichte, Cousin/e, Onkel, Neffe<br>14 - Andere verwandtschaftliche Beziehung<br>20 - Keine verwandtschaftliche Beziehung<br>---<br>(-1) - Weiss nicht<br>(-2) - Keine Antwort		
05340	LINK : ALL<br>---<br>Haben Sie Kinder, wo nicht (mehr) in Ihrem Haushalt leben?<br>---<br>1 - Ja<br>2 - Nein → **055.00**<br>---<br>(-1) - Weiss nicht → **055.00**<br>(-2) - Keine Antwort → **055.00**	Soziodemografie<br><br>TSODE83	BFS - SGB
05341	LINK : IF 05340={1}<br>---<br>Wie viele?<br><br><xx> - Anzahl Kinder (1 bis 15)<br>---<br>(-1) - Weiss nicht<br>(-2) - Keine Antwort	Soziodemografie<br><br>TSODE84	BFS - SGB
05500	LINK : ALL<br>---<br>Und zum Haus oder Wohnung, wo sie normalerweise drin wohnen. Sind Sie oder eine andere Person in Ihrem Haushalt...<br><br>*INT : Hauptkategorien vorlesen! Dann Unterkategorien je nach Hautkategorie*<br>---<br>A - MIETER(IN) oder GENOSSENSCHAFTER(IN)<br>B - EIGENTÜMER(IN) oder MITEIGENTÜMER(IN)<br>C - BEWOHNER(IN) von einem anderen WOHNUNGSTYP<br>1 - Mieter/in von dieser Wohnung/diesem Haus/Studio/Zimmer<br>2 - Mieter/in von dieser Genossenschaftswohnung<br>3 - Pächter/in<br>4 - Eigentümer/in von diesem Haus	Soziodemographie<br>Wohnsituation<br><br>TSODE101	BFS Standard - SHAPE 2011<br>(Q 157.00)

	5 - Eigentümer/in von dieser Wohnung (Stockwerkeigentümer/in)<br>6 - Bewohner/in von einer Dienstwohnung, wo dem Arbeitgeber gehört<br>7 - Bewohner/in von einer Freiwohnung, wo einem Verwandten/Freund gehört<br>8 - Nutzniesser/in<br>9 - Nutzniesser/in eines Wohnrechts<br>---<br>(-1) - Weiss nicht<br>(-2) - Keine Antwort		
i5700	LINK : ALL<br>---<br>Bei den nächsten Fragen geht es um Ihre Herkunft.<br>---<br>1 - Weiter	Soziodemographie<br>Nationalität<br><br>i5700	BFS – SGB
05700	LINK : NOT ASKED<br>---<br>- NationalityID (CH=8100 / <> 8100 andere!) _ _ _ _<br><br>*INT : Die Nationalität stammt aus dem kommunalen oder kantonalen Einwohnerregister. Sie wird nicht abgefragt.*	Soziodemographie<br>Nationalität<br><br>NationalityId	Ab 2012 BFS Standard - SHAPE 2011 (Q 103.01)<br>*Info aus Register*
05701	LINK : NOT ASKED<br>---<br>Das Geburtsland stammt aus dem kommunalen oder kantonalen Einwohnerregister. Es wird nicht abgefragt<br><br>- CountryIdOfBirth (CH=8100 / <> 8100 andere!) _ _ _ _	Soziodemographie<br>Nationalität<br><br>CountryIdOfBirth	BFS Standard - SHAPE 2011 (Q 103.02)<br>*Info aus Register*
05702	LINK : IF 05700={8100}<br><br>Falls Nationalität Schweiz ist (NATIONALITYID =8100)<br>---<br>Haben Sie neben der Schweizer noch eine andere Nationalität?<br>---<br>1 - Ja<br>2 - Nein → **057.20**<br>---<br>(-1) - Weiss nicht → **057.20**<br>(-2) - Keine Antwort → **057.20**	Soziodemographie<br>Nationalität<br><br>TSODE94	Ab 2012 BFS Standard - SHAPE 2011 (Q 105.00)

05703	LINK : IF 05702={1}<br><br>Falls Nationalität Schweiz ist (NATIONALITYID =8100) und die Person noch eine weitere Nationalität hat (TSODE94/05702=1)<br>--<br>Was haben Sie neben der Schweizer Nationalität noch für eine andere Nationalität?<br><br>TSODE95 :<br><91> - Suche LAND oder STAAT in Datenbank<br><br>TSODE95A :<br><xxxx> LAND<br>--<br>(-1) - Weiss nicht<br>(-2) - Keine Antwort	Soziodemographie Nationalität<br><br><br>TSODE95<br>[TSODE95a]	Ab 2012 BFS Standard - SHAPE 2011 (Q 105.01)
05710<br><br><br>*PROXY*	LINK : IF 05701≠{8100} AND NOT PROXY<br><br>Diese Frage geht an Personen die nicht in der Schweiz geboren sind (COUNTRYIDOFBIRTH < > 8100)<br>--<br>Seit welchem Jahr leben Sie in der Schweiz?<br><br>- Jahr _ _ _ _<br><br>*INT : Jahr eingeben!*<br>--<br>(-1) - Weiss nicht<br>(-2) - Keine Antwort	Soziodemographie Nationalität<br><br><br>TSODE96	Ab 2012 BFS Standard - SHAPE 2011 (Q 142.00)
05720	LINK : ALL<br><br>Wieder an alle<br>--<br>Sind Sie bei Ihrer Geburt Schweizer/-in, ausländischer Nationalität oder Doppelbürger/-in gewesen?<br>--<br>50 - Schweizer/-in → **057.40**<br>51 - Ausländischer Nationalität<br>52 - Doppelbürger/-in (Schweizer/-in und ausländ. National.) → **057.40**<br>53 - Staatenlos → **057.31**<br>--<br>(-1) - Weiss nicht → **057.31**<br>(-2) - Keine Antwort → **057.31**	Soziodemographie Nationalität<br><br><br>TSODE97	BFS Standard - SHAPE 2011 (Q 143.00)

05721	LINK : IF 05720={51}<br><br>Nur Personen die bei Geburt eine ausländische Nationalität hatten (TSODE97/57.20=51)<br>---<br>Was haben Sie bei Ihrer Geburt für eine Nationalität gehabt?<br><br>TSODE98<br>91 - Suche LAND oder STAAT in Datenbank<br><br>TSODE98A<br><xxxx> LAND<br><br>*INT : Land oder Staat eingeben!*<br>---<br>(-1) - Weiss nicht<br>(-2) - Keine Antwort	Soziodemographie<br>Nationalität<br><br><br>TSODE98<br>[TSODE98a]	BFS Standard - SHAPE 2011<br>(Q 144.00)
05730<br><br><br>*PROXY*	LINK : IF 05720={51,53,-1,-2} AND 05700={8100} AND NOT PROXY<br>---<br>In welchem Jahr haben Sie das Schweizer Bürgerrecht bekommen?<br><br><xxxx> Jahr<br><br>*INT : Jahr eingeben!*<br><br><br>---<br>(-1) - Weiss nicht<br>(-2) - Keine Antwort	Soziodemographie<br>Nationalität<br><br><br>TSODE102	BFS Standard - SHAPE 2011<br>(Q 145.00)
05740<br><br><br>*PROXY*	LINK : ALL AND NOT PROXY<br>---<br>In welchem Land ist Ihr Vater geboren?<br><br>TSODE103a<br><xxxx> LAND<br><br>*INT : Gegebenenfalls der Adoptivvater.*<br>---<br>50 - In der Schweiz<br>91 - Suche LAND oder STAAT in Datenbank<br>---<br>(-1) - Weiss nicht<br>(-2) - Keine Antwort	Soziodemographie<br>Nationalität<br><br><br>TSODE103<br>[TSODE103a]	BFS – SGB

05741<br><br>*PROXY*	LINK : ALL AND NOT PROXY<br>--<br>In welchem Land ist Ihre Mutter geboren?<br><br>TSODE104a<br><xxxx> LAND<br><br>*INT : Gegebenenfalls der Adoptivmutter*<br>--<br>50 - In der Schweiz<br>91 - Suche LAND oder STAAT in Datenbank<br>--<br>(-1) - Weiss nicht<br>(-2) - Keine Antwort	Soziodemographie<br>Nationalität<br><br><br>TSODE104<br>[TSODE104a]	BFS – SGB
05810	LINK : ALL<br>--<br>Wie hoch ist Ihr persönliches monatliches Nettoeinkommen, nach Abzug von den obligatorischen Sozialversicherungsbeiträgen und den Pensionskassenbeiträgen, zuzüglich oder abzüglich von allfälligen Alimenten?<br>Wieviel ist es ungefähr?<br><br><xxxxxx>- Franken pro Monat weiter zu 058.00<br>--<br>(-1) - Weiss nicht<br>(-2) - Keine Antwort	Soziodemographie<br>Einkommen<br><br><br>TSODE42	BFS – SGB
05811	LINK : IF 05810={-1,-2}<br><br>Nur falls die Person ihr persönliches Nettoeinkommen nicht kennt oder nicht angeben will (TSODE42/58.10 = -1, -2).<br>--<br>Ist es mehr als 4'500.- Fr. pro Monat oder weniger?<br>--<br>1 - Weniger als 4500.- fr.<br>2 - Genau 4500.- fr. → **058.00**<br>3 - Mehr als 4500.- fr. → **058.13**<br>--<br>(-1) - Weiss nicht → **058.00**<br>(-2) - Keine Antwort → **058.00**	Soziodemographie<br>Einkommen<br><br><br>TSODE48	BFS – SGB
05812	LINK : IF 05811={1}<br><br>Falls das persönliche Einkommen unter 4'500 Franken liegt.	Soziodemographie<br>Einkommen	VWI BE (R. Leu)

	--<br>Liegt es unter oder über 3'000 Franken pro Monat?<br>--<br>1 - Weniger als 3000.- fr. → **058.00**<br>2 - Genau 3000.- fr. → **058.00**<br>3 - Mehr als 3000.- fr. → **058.00**<br>--<br>(-1) - Weiss nicht → **058.00**<br>(-2) - Keine Antwort → **058.00**	TSODE49	
05813	LINK : IF 05811={3}<br><br>Falls das persönliche Einkommen unter 4'500 Franken liegt.<br>--<br>Liegt es unter oder über 6'000 Franken pro Monat?<br>--<br>1 - Weniger als 6000.- fr. → **058.00**<br>2 - Genau 6000.- fr. → **058.00**<br>3 - Mehr als 6000.- fr.<br>--<br>(-1) - Weiss nicht → **058.00**<br>(-2) - Keine Antwort → **058.00**	Soziodemographie<br>Einkommen<br><br><br>TSODE50	VWI BE(R. Leu)<br>*SGB97: TSODE41*
05814	LINK : IF 05813={3}<br><br>Falls das persönliche Einkommen über 6000 Franken liegt.<br>--<br>Liegt es unter oder über 9'000 Franken pro Monat?<br>--<br>1 - Weniger als 9000.- fr.<br>2 - Genau 9000.- fr.<br>3 - Mehr als 9000.- fr.<br>--<br>(-1) - Weiss nicht<br>(-2) - Keine Antwort	Soziodemographie<br>Einkommen<br><br><br>TSODE81	ISPM (M. Zwahlen)
05800	LINK : IF NBPERS_b>={2}<br><br>Für Haushalte mit mindestens 2 Personen (NBPERS_b>=2).<br>1 Personen-Haushalte weiter zu Frage 59.00<br>--<br>Und wie hoch ungefähr ist das gesamte monatliche Nettoeinkommen von Ihrem Haushalt?<br>Das heisst die Summe von allen Einkommen von allen Haushaltsmitgliedern zusammengezählt, wieder nach Abzug von den obligatorischen	Soziodemographie<br>Einkommen<br><br><br>TSODE40	BFS – SGB<br>*SGB92: TSODE17*

	Sozialversicherungsbeiträgen und den Pensionskassenbeiträgen, zuzüglich oder abzüglich von allfälligen Alimente.<br>Wieviel ist es ungefähr?<br><br><xxxxxx>- Franken pro Monat weiter zu 059.00<br>--<br>(-1) - Weiss nicht<br>(-2) - Keine Antwort		
05801	LINK : IF 05800={-1,-2}<br><br>Nur falls die Person das genaue Haushaltseinkommen nicht kennt oder nicht angeben will (TSODE40/58.00 = -1, -2).<br>--<br>Ist es mehr als 6'000.- Frs. pro Monat oder weniger?<br>--<br>1 - Weniger als 6000.- fr.<br>2 - Genau 6000.- fr. → **059.00**<br>3 - Mehr als 6000.- fr. → **058.03**<br>--<br>(-1) - Weiss nicht → **059.00**<br>(-2) - Keine Antwort → **059.00**	Soziodemographie<br>Einkommen<br><br><br>TSODE44	BFS – SGB
05802	LINK : IF 05801={1}<br><br>Falls das Einkommen weniger als 6'000 Franken beträgt.<br>--<br>Liegt es unter oder über 4'500 Franken pro Monat?<br>--<br>1 - Weniger als 4500.- fr. → **059.00**<br>2 - Genau 4500.- fr. → **059.00**<br>3 - Mehr als 4500.- fr. → **059.00**<br>--<br>(-1) - Weiss nicht → **059.00**<br>(-2) - Keine Antwort → **059.00**	Soziodemographie<br>Einkommen<br><br><br>TSODE45	VWI BE (R. Leu)
05803	LINK : IF 05801={3}<br><br>Falls das Einkommen über 6'000 liegt.<br>--<br>Liegt es unter oder über 9‘000 Franken pro Monat?<br>--<br>1 - Weniger als 9000.- fr.<br>2 - Genau 9000.- fr.<br>3 - Mehr als 9000.- fr.	Soziodemographie<br>Einkommen<br><br><br>TSODE46	VWI BE (R. Leu)<br>*SGB97: TSODE39*

	---<br>(-1) - Weiss nicht<br>(-2) - Keine Antwort		
05900<br><br>*PROXY*	LINK : IF NOT PROXY<br>---<br>Jetzt sind wir am Ende von dieser Umfrage. Wir danken Ihnen sehr fürs Mitmachen und dass Sie sich die Zeit genommen haben. Wie im Brief angekündigt, gibt es noch einen zweiten Teil in Form von einem kurzen Fragebogen. Sie können ihn auch übers Internet ausfüllen. Dazu bräuchte ich aber noch Ihre E-Mail-Adresse. Darf ich diese aufnehmen, damit wir Ihnen per Mail die Zugangsdaten zum Fragebogen schicken können?<br><br>*INT : Selbstverständlich geben wir keine persönlichen Daten oder Ihre Maildresse an Dritte weiter*<br>---<br>1 - OK für die Aufnahme Email-Adresse<br>2 - Nein, will nicht / kann nicht im Internet ausfüllen<br>---<br>(-1) - Weiss nicht<br>(-2) - Keine Antwort	Abschluss<br><br>TABSC04	BFS – SGB
05901<br><br>*PROXY*	LINK : IF 05900={2,-1,-2}<br>---<br>In dem Fall schicken wir Ihnen den schriftlichen Fragebogen per Post zu. Damit dieser schriftliche Fragebogen auch wirklich richtig ankommt, sollte ich noch einmal Ihre Adresse kontrollieren.<br>---<br>1 - OK für Adressverifikation<br>2 - Verweigerung des schriftlichen Fragebogen	Abschluss<br><br>TABSC05	BFS – SGB
05905<br><br>*PROXY*	LINK : IF 05901={1} OR 05906={1}<br>---<br>Familien-Namen<br>Vornamen<br>Strasse + Nummer<br>c/o Anschrift (wohnt bei.)<br>PLZ/Ort<br><br>*INT : EINGEBLENDETE ADRESSE VERGLEICHEN & GGF. KORRIGIEREN*<br>---<br>90 - Adresse Korrekt	Abschluss<br><br>[T_ADRESS]	BFS – SGB
05902<br><br>*PROXY*	LINK : IF 05900={1}<br>---<br>Bitte geben Sie mir den Adressteil an, wo VOR dem @-Zeichen ("@ = Sprich: ät") kommt.	Abschluss<br><br>[T_EMAIL_a]	BFS – SGB

	INT : Adresse Buchstabe für Buchstabe erfassen!<br>*Achtung: "-" = Bindestrich/Minus | "_" = Underscore/Unterstrich*		
05903<br><br>*PROXY*	LINK : IF 05900={1}<br>---<br>Bitte geben Sie mir den Adressteil an, wo NACH dem @-Zeichen ("@ = Sprich: ät") kommt.<br><br>*INT : Auswahl aus Liste der häufigsten Provider!*<br>*Falls Provider nicht in Liste - Manuell erfassen mit code <95> !*<br>*Achtung: "-" = Bindestrich/Minus | "_" = Underscore/Unterstrich*	Abschluss<br><br>[T_EMAIL_b]	BFS – SGB
05904<br><br>*PROXY*	LINK : IF 05900={1}<br>---<br>VERIFIKATION DER E-MAIL-ADRESSE<br><br>E-Mail:<br>******* [Einblendung E-Mail Adresse {Teil1} @ {Teil2}]<br><br>*INT : E-Mail Adresse buchstabieren!*<br>---<br>1 - E-Mail Adresse OK<br>2 - Korrigieren -> Zurück zus Aufnahme E-Mail Adresse 1. Teil	Abschluss<br><br>[T_CONF_EMAIL]	BFS – SGB
05906<br><br>*PROXY*	LINK : IF 05904 ={1}<br>---<br>Sie erhalten in den nächsten 30 Minuten eine E-Mail mit dem Zugangscode. Sie bekommen den Zugangscode zusätzlich per Post zugestellt. Damit der Brief auch wirklich ankommt, sollte ich noch Ihre Adresse kontrollieren.<br>---<br>1 - OK für Adressverifikation<br>2 - Verweigerung	Abschluss<br><br>TABSC06	BFS – SGB
05907	LINK : ALL<br>---<br>Damit sind wir am Schluss vom Interview. Wir wünschen Ihnen also noch einen schönen Tag/Abend und bedanken uns bei Ihnen für Ihre wertvollen Auskünfte	Abschluss<br><br>[T_FIN]	BFS – SGB

Schriftlicher Fragebogen

Nr.	Fragen	Thema	Quelle / Bemerkungen
S0010<br><br><br>*PAPER*	Wer sind Sie?<br>Mann / Frau<br><br>Geburtsdatum<br>TT / MM / JJJJ	Soziodemografie<br><br><br>SSEX<br>SALTER<br>[SGEBTT]<br>[SGEBMM]<br>[SGEBJJ]	BFS – SGB<br>*Kontrollvariablen*<br>*SGB17: Im papier Version wurde das vollständige Geburtsdatum erhoben*
S0010_WEB<br><br><br>*ESURVEY*	Bitte bestätigen Sie folgende Angaben: Sie sind {Vorname} {Name}, {Sex}, {Geburtsdatum}<br>---<br>1 - Ja<br>2 - Nein	Soziodemografie<br><br><br>SSEX<br>SALTER<br>[SGEBTT]<br>[SGEBMM]<br>[SGEBJJ]	BFS – SGB<br>*Kontrollvariablen*<br>*SGB07 und SGB12 wurde nur das Geburtsdatum erhoben*
S0100<br><br><br>1 / {1}	Wie wichtig ist die Gesundheit für Sie? Sie sehen hier 3 verschiedene Meinungen. Geben Sie bitte an, welche mit Ihrer eigenen Meinung am ehesten übereinstimmt.<br><br>*INT : Bitte nur ein einziges Kästchen ankreuzen!*<br><br>*WEB : Bitte nur eine einzige Antwort auswählen!*<br>---<br>1 - Ich lebe, ohne mich um mögliche Folgen für meine Gesundheit zu kümmern<br>2 - Gedanken an die Erhaltung meiner Gesundheit beeinflussen meinen Lebensstil<br>3 - Gesundheitliche Überlegungen bestimmen weitgehend, wie ich lebe	Gesundheitsbedeutung<br><br><br>SGEBE01	SOMIPOPS écrit 2
S6400<br><br><br>2 / {2}	Wie haben Sie sich in den letzten 4 Wochen gefühlt?<br>a) Voller Leben<br>b) Voller Energie<br>c) Erschöpft<br>d) Müde<br><br>*INT : Bitte für jede Linie die entsprechende Antwort ankreuzen!*<br><br>*WEB : Bitte für jede Zeile die entsprechende Antwort auswählen!*<br>---<br>1 - Immer<br>2 - Meistens<br>3 - Manchmal<br>4 - Selten<br>5 - Nie	Psychische Gesundheit<br><br><br>SPSYG07a<br>SPSYG07b<br>SPSYG07c<br>SPSYG07d	MOS SF-36 VT<br>*SGB17: im schriftlich verschoben*<br>*(SGB12: TPSYG16/20/22/24)*<br>*(Indizes EVI)*

S0200<br><br>3 / {3}	Wenn Sie über Ihr Leben nachdenken, inwiefern stimmen Sie folgenden Aussagen zu?<br><br>a) Ich werde mit einigen meiner Probleme nicht fertig<br>b) Ich fühle mich im Leben gelegentlich hin- und hergeworfen<br>c) Ich habe wenig Kontrolle über die Dinge, die ich erlebe<br>d) Oft fühle ich mich meinen Problemen ausgeliefert<br><br>*INT : Bitte für jede Linie die entsprechende Antwort ankreuzen!*<br><br>*WEB : Bitte für jede Zeile die entsprechende Antwort auswählen!*<br>--<br>1 - Stimme voll und ganz zu<br>2 - Stimme eher zu<br>3 - Stimme eher nicht zu<br>4 - Stimme überhaupt nicht zu	Psychische Gesundheit<br><br><br>SPSYG01<br>SPSYG02<br>SPSYG03<br>SPSYG04	IGIP écrit 4<br>*(Indizes: MASTERY)*
S0500<br><br>4 / {4}	Wie häufig passiert es Ihnen, dass Sie...<br><br>a) Schlecht einschlafen?<br>b) Einen unruhigen Schlaf haben?<br>c) Nachts mehrmals erwachen?<br>d) Morgens zu früh erwachen?<br><br>*INT : Bitte für jede Linie die entsprechende Antwort ankreuzen!*<br><br>*WEB : Bitte für jede Zeile die entsprechende Antwort auswählen!*<br>--<br>1 - Häufig<br>2 - Manchmal<br>3 - Selten<br>4 - Nie	Körperliche Beschwerden<br><br><br>SKRSY02a<br>SKRSY02b<br>SKRSY02c<br>SKRSY02d	Leben und Gesundheit in Deutschland<br>*(Indizes: SOMMEIL)*
S0400<br><br><br>5 / {5}<br><br><br>*PAPER*	Hatten Sie in den letzten 12 Monaten eine Unfallverletzung? Falls ja, welche Behandlung haben Sie gebraucht, und wie lange waren Sie deshalb arbeitsunfähig?<br><br>a) Arbeitsunfall<br>b) Verkehrsunfall<br>c) Unfall im Haus oder Garten<br>d) Unfall bei Sport, Spiel, usw<br><br>Arbeitsunfähigkeit: _ _ _ Tage (SUNFA01-04b)<br><br><br>*INT : Hausfrauen und Rentner: unfähig, die üblichen Arbeiten im Haushalt zu verrichten.*<br>*Schüler/Studenten: unfähig, zum Unterricht zu gehen.*	Unfälle<br><br><br>SUNFA01a<br>SUNFA02a<br>SUNFA03a<br>SUNFA04a<br>SUNFA01b<br>SUNFA02b<br>SUNFA03b<br>SUNFA04b	IGIP écrit 6<br>*(SGB 1997 Anzahl Tage nur 2stellig)*

	--<br>1 - Nein<br>2 - Ja, selbst behandelt<br>3 - Ja, ambulant behandelt<br>4 - Ja, im Spital behandelt		
S0400_WEB_2<br><br><br>5.1 / {5.1}<br><br><br>*ESURVEY*	LINK : S0400_web_2_a IF S0400_WEB_1_a={2,3,4}<br>S0400_web_2_b IF S0400_WEB_1_b={2,3,4}<br>S0400_web_2_c IF S0400_WEB_1_c={2,3,4}<br>S0400_web_2_d IF S0400_WEB_1_d={2,3,4}<br>--<br>Wie lange waren Sie aufgrund dieses Unfalls arbeitsunfähig?<br><br>a) Arbeitsunfall<br>b) Verkehrsunfall<br>c) Unfall im Haus oder Garten<br>d) Unfall bei Sport, Spiel, usw.<br><br>Arbeitsunfähigkeit: _ _ _ Tage<br><br>*WEB : Nicht-Erwerbstätige: unfähig, die üblichen Arbeiten im Haushalt zu verrichten.*<br>*Schüler/Studenten: unfähig, zum Unterricht zu gehen.*	Unfälle<br><br><br>SUNFA01b<br>SUNFA02b<br>SUNFA03b<br>SUNFA04b	IGIP écrit 6<br>*(SGB 1997 Anzahl Tage nur 2stellig)*
S0400_WEB_1<br><br><br>5 / {5}<br><br><br>*ESURVEY*	Hatten Sie in den letzten 12 Monaten eine Unfallverletzung? Falls ja, welche Behandlung haben Sie gebraucht?<br><br>a) Arbeitsunfall<br>b) Verkehrsunfall<br>c) Unfall im Haus oder Garten<br>d) Unfall bei Sport, Spiel, usw<br>--<br>1 - Nein<br>2 - Ja, selbst behandelt<br>3 - Ja, ambulant behandelt<br>4 - Ja, im Spital behandelt	Unfälle<br><br><br>SUNFA01a<br>SUNFA02a<br>SUNFA03a<br>SUNFA04a	IGIP écrit 6
S0600<br><br><br>6 / {6}	Wieviele Male sind Sie in den letzten 12 Monaten wegen Gesundheitsproblemen oder für Kontrolluntersuchungen bei einem der folgenden Spezialisten gewesen?<br><br>a) Zahnarzt/Zahnärztin _ _ mal<br>b) Dentalhygieniker/in _ _ mal<br>c) Physiotherapeut/in _ _ mal<br>d) Chiropraktiker/in _ _ mal<br>e) Psychologe/in, Psychotherapeut/in _ _ mal<br>f) Heilpraktiker/in, Naturheiler/in _ _ mal	Inanspruchnahme mediz. Dienstleistungen Paramedizin<br><br><br>SINAN01<br>SINAN09<br>SINAN02<br>SINAN03	SOMIPOPS écrit 13<br>*Ergänzt mit:*<br>*SGB02: SINAN09*

	INT : Wenn Sie nie bei dem betreffenden Spezialisten waren, tragen Sie bitte jedesmal die Zahl "0" ein.<br><br>*WEB : Wenn Sie nie bei dem betreffenden Spezialisten waren, tragen Sie bitte jedesmal die Zahl "0" ein.*<br>*Die maximale Anzahl Konsultationen beträgt 99.*	SINAN05<br>SINAN08	
S0700<br><br><br>7 / {7}	Wieviele Male haben Sie in den letzten 12 Monaten eine der folgenden Therapien in Anspruch genommen?<br><br>a) Akupunktur _ _ mal<br>b) Traditionelle chinesische Medizin (ohne Akupunktur) _ _ mal<br>c) Homöopathie _ _ mal<br>d) Kräutermedizin bzw. Pflanzenheilkunde _ _ mal<br>e) Shiatsu / Fussreflexzonenmassage _ _ mal<br>f) Indische Medizin / Ayurveda _ _ mal<br>g) Osteopathie _ _ mal<br>h) Andere Therapien, z.B. Kinesiologie, Feldenkrais, Autogenes Training Neuraltherapie, Bioresonanztherapie, Antroposophische Medizin. _ _ mal<br><br>*INT : Wenn Sie nie eine der folgenden Therapien besucht haben, tragen Sie bitte jedesmal die Zahl "0" ein.*<br><br>*WEB : Wenn Sie nie eine der folgenden Therapien besucht haben, tragen Sie bitte jedesmal die Zahl "0" ein.*<br>*Die maximale Anzahl Therapien beträgt 99.*	Inanspruchnahme Komplementärmedizin<br><br><br>SINAN20<br>SINAN27<br>SINAN21<br>SINAN22<br>SINAN24<br>SINAN30<br>SINAN31<br>SINAN33	BFS – SGB<br>*ergänzt mit:*<br>*SGB97: SINAN22/24*<br>*SGB02: SINAN26-28*<br>*SGB07: SINAN29-32*<br>*SGB12: SINAN33*
S0800<br><br><br>8 / {8}	Wieviele Male haben Sie sich in den letzten 12 Monaten in einer Apotheke wegen einem Gesundheitsproblem beraten lassen?<br><br>- _ _ mal<br><br>*INT : Falls Sie sich dort nicht beraten liessen, tragen Sie bitte die Zahl "0" ein!*<br><br>*WEB : Falls Sie sich dort nicht beraten liessen, tragen Sie bitte die Zahl "0" ein!*<br>*Die maximale Anzahl Beratungen beträgt 99.*	Inanspruchnahme Apotheke<br><br><br>SINAN13	BFS – SGB
S1600<br><br><br>9 / {9}	Haben Sie sich schon einmal gegen die Grippe impfen lassen?<br>Falls ja, wann das letzte Mal?<br><br>Das letzte Mal am: _ _ Monat (SIMPF05a) _ _ _ _ Jahr (SIMPF05b<br><br>*INT : Bitte geben Sie den Monat und das Jahr als Zahl an!*<br><br>*WEB : Bitte geben Sie den Monat und das Jahr an!*<br>---<br>1 - Ja<br>2 - Nein	Impfung gegen Grippe<br><br><br>SIMPF04<br>SIMPF05a<br>SIMPF05b	BFS – SGB
S1700	Bitte geben Sie für die zwei folgenden Aussagen an, inwieweit die Aussage für Sie zutrifft oder nicht zutrifft.	Organspende	BAG - Sozialforschungsstelle Uni Zürich (2005)

10 / {10}	a) Ich habe mich persönlich schon mit dem Thema Organspende auseinandergesetzt.<br>b) Ich persönlich wäre dazu bereit, unmittelbar nach meinem Tod eines meiner Organe (Gewebe oder Zellen) zu spenden.<br><br>*INT : Ihre Antworten können Sie mit den Werten zwischen 1 (trifft überhaupt nicht zu) bis 6 (trifft voll und ganz zu) variieren.*<br><br>*WEB : Ihre Antworten können Sie mit den Werten zwischen 1 (trifft überhaupt nicht zu) bis 6 (trifft voll und ganz zu) variieren.*<br>---<br>1 - 1.Trifft überhaupt nicht zu<br>2 - 2.<br>3 - 3.<br>4 - 4.<br>5 - 5.<br>6 - 6.Trifft voll und ganz zu	SOSPD01a<br>SOSPD01b	
S1800<br><br>11 / {11}	Besitzen Sie eine Spendekarte (Organspendeausweis)?<br>---<br>1 - Ja<br>2 - Nein	Organsspende<br><br>SOSPD02	BAG - Sozialforschungsstelle Uni Zürich (2005)
S1900<br><br>12 / {12}	Wissen Ihre nächsten Angehörigen (Familie), ob Sie nach Ihrem Tod Organe spenden möchten oder nicht?<br>---<br>1 - Ja<br>2 - Nein<br>---<br>(-1) - Weiss nicht	Organsspende<br><br>SOSPD03	BAG - Sozialforschungsstelle Uni Zürich (2005)
S2000<br><br>13 / {13}	LINK : IF ALTER <=74<br><br>Die Frage 13 ist nur von 15-74-jährigen Personen zu beantworten. Alle 75-jährigen und älteren Personen gehen bitte weiter zu Frage 14.<br>---<br>Benützen Sie bzw. Ihr/e Partner/in irgendeine Methode zur Empfängnisverhütung?<br>---<br>1 - Ja<br>2 - Nein **→Weiter zu Frage 14 S22.00**	Empfängnisverhütung<br><br>SFAPL01	BFS – SGB
S2100<br><br>13.1 / {13.1}	LINK : IF S2000={1}<br>---<br>Welche Mittel benützen Sie - Sie selber oder Ihr/e Partner/in - zur Empfängnisverhütung?<br><br>a) Pille<br>b) Verhütungspflaster<br>c) Verhütungsring<br>d) 3-Monatsspritze<br>e) Verhütungsstäbchen	Empfängnisverhütung<br><br>SFAPL22a<br>SFAPL22b<br>SFAPL22c<br>SFAPL22d<br>SFAPL22e<br>SFAPL22f	Anal. IGIP tel 75.00<br>*SGB17: Trennung Sterilisation der Frau / des Mannes;*<br>*Trennung Hormonspirale / Kupferspirale*

	f) "Pille danach"<br>g) Hormonspirale<br>h) Kupferspirale, Kupferkette<br>i) Diaphragma oder Portiokappe<br>j) Frauenkondom (Femidom)<br>k) Präservativ / Kondom<br>l) Sterilisation der Frau (Tubenligatur, etc.)<br>m) Sterilisation des Mannes (Vasektomie, etc.)<br>n) Natürliche Empfängnisverhütung (Temperatur messen, Schleim beobachten, etc.)<br>o) Andere Methoden<br><br>*INT : Bitte alles Zutreffende ankreuzen!*<br><br>*WEB : Bitte alles Zutreffende auswählen!*<br>---<br>1 - Trifft zu<br>0 - Trifft nicht zu	SFAPL22p<br>SFAPL22q<br>SFAPL22h<br>SFAPL22i<br>SFAPL22j<br>SFAPL22n<br>SFAPL22o<br>SFAPL22l<br>SFAPL22m	
S2200<br><br>14 / {14}	WIEDER AN ALLE<br>---<br>Welche Art von Krankenversicherung haben Sie persönlich für die obligatorische Grundversicherung?<br><br>*INT : Bitte nur ein einziges Kästchen ankreuzen!*<br><br>*WEB : Bitte nur eine einzige Antwort auswählen!*<br>---<br>1 - Gewöhnliche Krankenversicherung mit Franchise<br>2 - Bonus-Versicherung<br>3 - HMO-Versicherung<br>4 - Hausarztmodell<br>5 - Versicherungsmodell mit vorgängiger telefonischer Beratung vor jedem Arztbesuch (Telefonmodell)<br>---<br>(-1) - Weiss nicht	Soziale Sicherheit<br>Krankenversicherung<br><br><br>SSOSI11	BFS – SGB<br>*SGB12: in schr. FB verschoben (SGB07= TSOSI18)*
S2500<br><br>15 / {15}	LINK : IF GEBJAHR>=1900 AND GEBJAHR <=1998<br><br>Personen mit Jahrgang 1900 bis 1998<br>---<br>Wie hoch ist Ihre persönliche Jahresfranchise?<br><br>*INT : Bitte nur ein einziges Kästchen ankreuzen!*<br><br>*WEB : Bitte nur eine einzige Antwort auswählen!*	Soziale Sicherheit<br>Krankenversicherung<br><br><br>SSOSI15	BFS – SGB<br>*SGB12: in schr. FB verschoben (SGB07= TSOSI27/28)*<br>**NB. Die Jahresfranchise ändert zum darauffolgenden Jahr des erreichten 18. Altersjahr.*

	---<br>1 - CHF 300.-<br>2 - CHF 500.-<br>3 - CHF 1'000.-<br>4 - CHF 1'500.-<br>5 - CHF 2'000.-<br>6 - CHF 2'500.-<br>---<br>(-1) - Weiss nicht		
S2501<br><br><br>15.1 / {15.1}	LINK : F GEBJAHR>=1999 AND GEBJAHR <=2002<br>NOT_FL<br><br>Junge Erwachsene mit Jahrgang 1999 bis 2002<br>---<br>Wie hoch ist Ihre persönliche Jahresfranchise?<br><br>*INT : Bitte nur ein einziges Kästchen ankreuzen!*<br><br>*WEB : Bitte nur eine einzige Antwort auswählen!*<br>---<br>1 - CHF 0.-<br>2 - CHF 100.-<br>3 - CHF 200.-<br>4 - CHF 300.-<br>5 - CHF 400.-<br>6 - CHF 500.-<br>7 - CHF 600.-<br>---<br>(-1) - Weiss nicht	Soziale Sicherheit<br>Krankenversicherung<br><br><br>SSOSI16	BFS – SGB<br>*SGB12: in schr. FB verschoben (SGB07= TSOSI27/28)*<br>**NB. Die Jahresfranchise ändert zum darauffolgenden Jahr des erreichten 18. Altersjahr.*
S2700<br><br><br>16 / {16}	Haben Sie eine Zusatzversicherung für die Komplementärmedizin?<br>---<br>1 - Ja<br>2 - Nein<br>---<br>(-1) - Weiss nicht	Soziale Sicherheit<br>Krankenversicherung<br><br><br>SSOSI18	BAG (T. Bandi)
S2800<br><br><br>17 / {17}	Wie sind Sie versichert, wenn Sie ins Spital gehen müssen?<br><br>*INT : Bitte nur ein einziges Kästchen ankreuzen!*<br><br>*WEB : Bitte nur eine einzige Antwort auswählen!*	Soziale Sicherheit<br>Krankenversicherung<br><br><br>SSOSI19	SGB 1992<br>*SGB12: Pkt2 gestrichen / + in schr. FB verschoben (SGB07= TSOSI25)*

	---<br>1 - Allgemeine Abteilung<br>3 - Halbprivate Abteilung<br>4 - Private Abteilung<br>5 - Anderes Modell<br>---<br>(-1) - Weiss nicht		
S2900<br><br>18 / {18}	Wie wichtig ist es für Sie, den Spezialisten, den man konsultieren will, selber auszuwählen?<br>---<br>1 - Sehr wichtig<br>2 - Eher wichtig<br>3 - Eher nicht wichtig<br>4 - Überhaupt nicht wichtig	Soziale Sicherheit<br>Krankenversicherung<br><br>SSOSI20	SGB 1997<br>*SGB12: in schr. FB verschoben (SGB97-07=TSOSI11)*
S3300<br><br>19 / {19}	Viele Leute - Sie vielleicht auch - legen Wert auf eine gesunde Ernährung. Sehen Sie Hindernisse für jemanden, der sich gesund ernähren möchte?<br><br>a) Hoher Zeitaufwand für Einkaufen und Zubereitung<br>b) Zu wenig Angebote in den Geschäften<br>c) Zu wenig Angebote in Restaurants, Kantinen, usw.<br>d) Gesundes Essen ist relativ teuer<br>e) Zu wenig Unterstützung durch Mitmenschen<br>f) Mitmenschen halten davon ab<br>g) Grosse Vorliebe für gutes Essen<br>h) Grosse Vorliebe für reichliches Essen<br>i) Gewohnheiten und Zwänge des Alltags<br>j) Fehlender Wille, fehlender Glaube an Erfolg<br><br>*INT : Bitte alles Zutreffende ankreuzen!*<br><br>*WEB : Bitte alles Zutreffende auswählen!*<br>---<br>1 - Trifft zu<br>0 - Trifft nicht zu	Ernährung<br><br>SERNA01a<br>SERNA01b<br>SERNA01c<br>SERNA01d<br>SERNA01e<br>SERNA01f<br>SERNA01g<br>SERNA01h<br>SERNA01i<br>SERNA01j	IGIP écrit 21
S6500<br><br>20 / {20}	Achten Sie derzeit auf Ihren Salzkonsum oder reduzieren Sie ihn?<br>---<br>1 - Ja<br>2 - Nein	Ernährung<br><br>SERNA02	BRFSS 2014
S6600<br><br>21 / {21}	Machen Sie Gymnastik, Fitness oder Sport (z.B. Fussball, Volleyball, Wandern, Reiten, Yoga etc.)?<br>---<br>1 - Ja<br>2 - Nein →**Weiter zu Frage 22 S69.00**	Körperliche Aktivität<br><br>SKOBW10	BFS – SGB<br>*SGB17: im schriftlich verschoben (SGB12: TKOBW06)*

S6700<br><br>21.1 / {21.1}	LINK : IF S6600=1<br>---<br>Wie häufig treiben Sie Gymnastik, Fitness oder Sport?<br><br>*INT : Bitte nur ein einziges Kästchen ankreuzen!*<br><br>*WEB : Bitte nur eine einzige Antwort auswählen!*<br>---<br>1 - Seltener als 1 Mal pro Monat **→Weiter zu Frage 22 S69.00**<br>2 - Etwa 1-3 Mal pro Monat **→Weiter zu Frage 22 S69.00**<br>3 - Etwa 1 Mal pro Woche<br>4 - Mehrmals wöchentlich<br>5 - Täglich oder beinahe täglich	Körperliche Aktivität<br><br>SKOBW11	BASPO<br>*SGB17: im schriftlich verschoben*<br>*(SGB12: TKOBW13)*
S6800<br><br>21.2 / {21.2}	LINK : IF S6700={3,4,5}<br>---<br>Wie viel Zeit verbringen Sie normalerweise in einer Woche insgesamt mit Gymnastik, Fitness oder Sport?<br><br>Insgesamt pro Woche _ _ Stunden _ _ Minuten<br><br>*INT : Geben Sie bitte einen Durchschnitt pro Woche an!*<br><br>*WEB : Geben Sie bitte einen Durchschnitt pro Woche in Stunden und Minuten an!*	Körperliche Aktivität<br><br>[SKOBW12a]<br>[SKOBW12b]<br>SKOBW12	BFS – SGB<br>*Variable im Datensatz ist in Minuten*
S6900<br><br>22 / {22}	WIEDER AN ALLE<br>---<br>Wie bewegen Sie sich normalerweise fort, um sich an verschiedene Orte zu begeben (hin und zurück) z.B. zur Arbeit, zur Schule, zum Einkaufen oder zum Markt, zum Ausgehen?<br><br>a) Mit öffentlichen Verkehrsmitteln<br>b) Mit einem motorisierten Fahrzeug<br>c) Mit dem Velo<br>d) Zu Fuss<br><br>*INT : Bitte alles Zutreffende ankreuzen!*<br><br>*WEB : Bitte alles Zutreffende auswählen!*<br>---<br>1 - Trifft zu<br>0 - Trifft nicht zu	Körperliche Aktivität<br><br>SKOBW13a<br>SKOBW13b<br>SKOBW13c<br>SKOBW13d	BFS – SGB<br>*SGB17: im schriftlich verschoben*<br>*(SGB12: TKOBW03a-c)*
S7000<br><br>22.1 / {22.1}	LINK : IF S6900_c={1} or IF S6900_d={1}<br><br>Die Frage richtet sich an Personen, die das Velo benützen oder zu Fuss unterwegs sind. Die anderen gehen weiter zu Frage S3810/23	Körperliche Aktivität<br><br>SKOBW14	BASPO<br>*SGB17: angepasst und im schriftlich eingefügt*<br>*(SGB12: TKOBW12)*

	---<br>Wie lange sind Sie normalerweise täglich insgesamt zu Fuss oder mit dem Velo unterwegs?<br><br>*INT : Bitte nur ein einziges Kästchen ankreuzen!*<br><br>*WEB : Bitte nur eine einzige Antwort auswählen!*<br>---<br>1 - 00 - 14 Minuten pro Tag<br>2 - 15 - 29 Minuten pro Tag<br>3 - 30 - 59 Minuten pro Tag<br>4 - 1 Stunde bis weniger als 2 Stunden pro Tag<br>5 - 2 Stunden bis weniger als 3 Stunden pro Tag<br>6 - 3 Stunden oder mehr pro Tag		
S3810<br><br>23 / {-}	LINK : IF 05200={1} OR 05201={1} OR 05202={1}<br>---<br>Die folgenden Fragen beziehen sich auf Ihre aktuelle Erwerbstätigkeit.<br><br>Geben Sie bei jeder Aussage die Antwort an, die am ehesten auf Ihre Situation bei der Arbeit zutrifft:<br><br>a) Ich muss mich beeilen, um meine Arbeit zu erledigen<br>b) Ich muss an zu viele Dinge gleichzeitig denken<br>c) Ich habe Mühe, Arbeit und Familienpflichten zu vereinbaren<br>d) Ich erlebe Spannungen im Umgang mit Leuten wie Kunden, Patienten, Schülern, Fahrgästen, Klienten<br>f) Meine Arbeit erfordert, dass ich meine Gefühle verberge<br>g) Ich habe manchmal Angst bei der Arbeit (um meine Sicherheit oder die der anderen usw.)<br>h) Ich kann bei meiner Aufgabe sehr wenig mitbestimmen, wie ich die Arbeit erledige<br>i) Ich kann dann Pause machen, wann ich möchte<br>e) Ich erlebe Stress bei der Arbeit<br>---<br>1 - Immer<br>2 - Meistens<br>3 - Manchmal<br>4 - Selten<br>5 - Nie<br>6 - Trifft nicht zu	Arbeitssituation<br><br><br>SARSI20a<br>SARSI20b<br>SARSI20c<br>SARSI20d<br>SARSI20f<br>SARSI20g<br>SARSI20h<br>SARSI20i<br>SARSI20e	ESPS2010; Fragen 1-4, 7-9<br>EWCS 51: Fragen 5-6<br>Antwortskala: EWCS 51 (5 Stufen)<br>*SGB17: Für die Fragen S3810 à S4900 wurde ein Filter auf Grundlage des CATI angewandt*
S7100<br><br>24 / {-}	LINK : IF S3810_e={1,2,3,4}<br>---<br>Wie sehr fühlen Sie sich im Stande, Ihren Stress bei der Arbeit zu bewältigen?<br>---<br>1 - Voll und ganz<br>2 - Ziemlich gut	Arbeitssituation<br><br><br>SARSI30	SECO, Stressstudie, 2010

	3 - Eher schlecht<br>4 - Überhaupt nicht		
S3900<br><br>25 / {-}	LINK : IF 05200={1} OR 05201={1} OR 05202={1}<br>--<br>Geben Sie bitte bei jeder Aussage die Antwort an, die am ehesten auf Ihre Situation bei der Arbeit zutrifft:<br><br>a) Ich kann bei meiner Arbeit immer wieder etwas Neues lernen<br>b) Ich kann meine Fähigkeiten voll einsetzen<br>c) Meine Kollegen/Kolleginnen helfen mir und unterstützen mich<br>d) Mein/e Vorgesetzte/r nimmt ernst, was ich sage<br>e) Mein/e Vorgesetzte/r hilft mir und unterstützt mich<br>f) Ich erhalte widersprüchliche Anordnungen oder Angaben<br>g) Ich habe das Gefühl, eine sinnvolle Arbeit zu machen<br>h) Meine Arbeit wird angemessen gewürdigt<br>i) Zu meiner Arbeit gehören Aufgaben, die meinen persönlichen Werten widersprechen<br>j) Ich habe die Mittel, um eine gute Arbeit zu leisten<br>--<br>1 - Immer<br>2 - Meistens<br>3 - Manchmal<br>4 - Selten<br>5 - Nie<br>6 - Trifft nicht zu	Arbeitssituation<br><br><br>SARSI21a<br>SARSI21b<br>SARSI21c<br>SARSI21d<br>SARSI21e<br>SARSI21f<br>SARSI21g<br>SARSI21h<br>SARSI21i<br>SARSI21j	ESPS2010; questions 1-2, 4, 7-8,10;<br>EWCS 51: Frage 3, 5, 7, 9;<br>Arbeitsbedingungen DARES 2005: Frage 6<br>Antwortskala: EWCS 51 (5 Stufen)
S4000<br><br>26 / {-}	LINK : IF 05200={1} OR 05201={1} OR 05202={1}<br>--<br>Geben Sie bitte anhand der Skala an, wie häufig beinhaltet Ihre Arbeit...<br><br>a) Ein hohes Arbeitstempo<br>b) Arbeiten unter Termindruck<br>--<br>1 - Die ganze Zeit oder fast die ganze Zeit<br>2 - Ungefähr drei Viertel der Zeit<br>3 - Ungefähr die Hälfte der Zeit<br>4 - Ungefähr ein Viertel der Zeit<br>5 - Nie oder fast nie	Arbeitssituation<br><br><br>SARSI22a<br>SARSI22b	EWCS 2010 Q45
S7200<br><br>27 / {-}	LINK : IF 05200={1} OR 05201={1} OR 05202={1}<br>--<br>Wie oft haben Sie in den letzten 12 Monaten in Ihrer Freizeit gearbeitet, um die Arbeitsanforderungen zu erfüllen?<br><br>*INT : Bitte nur ein einziges Kästchen ankreuzen!*<br><br>*WEB : Bitte nur eine einzige Antwort auswählen!*	Arbeitssituation<br><br><br>SARSI31	EWCS 2015 - Q46

	---<br>1 - Täglich<br>2 - Mehrmals pro Woche<br>3 - Mehrmals pro Monat<br>4 - Seltener<br>5 - Nie		
S7300<br><br>28 / {-}	LINK : IF 05200={1} OR 05201={1} OR 05202={1}<br>---<br>Haben Sie in den letzten 12 Monaten gearbeitet, obwohl Sie krank waren?<br>Wenn ja, wie viele Arbeitstage?<br>---<br>1 - Ja, ich habe gearbeitet, obwohl ich krank war<br>2 - Nein, ich habe nicht gearbeitet, als ich krank war<br>3 - Ich war nicht krank	Arbeitssituation<br><br>SARSI32a<br>SARSI32b	EWCS 2015 - Q84a Q84b
S4100<br><br>29 / {-}	LINK : IF 05200={1} OR 05201={1} OR 05202={1}<br>---<br>Wie häufig müssen Sie Ihre Arbeit wegen einer anderen unvorhergesehenen Aufgabe unterbrechen?<br><br>*INT : Bitte nur ein einziges Kästchen ankreuzen!*<br><br>*WEB : Bitte jeweils nur eine einzige Antwort auswählen!*<br>---<br>1 - Nie **→Weiter zur Frage 30 S42.00**<br>2 - Gelegentlich<br>3 - Ziemlich häufig<br>4 - Sehr häufig	Arbeitssituation<br><br>SARSI23	EWCS 2010 Q47
S4110<br><br>29.1 / {-}	LINK : IF S4100={2,3,4}<br>---<br>Sind diese Unterbrechungen für Ihre Arbeit:<br><br>*INT : Bitte nur ein einziges Kästchen ankreuzen!*<br>---<br>1 - Störend<br>2 - Ohne Auswirkungen<br>3 - Positiv	Arbeitssituation<br><br>SARSI24	EWCS 2010 Q48
S4200<br><br>30 / {-}	LINK : IF 05200={1} OR 05201={1} OR 05202={1}<br>---<br>Bitte geben Sie für die folgende Aussage an, inwieweit sie für Sie zutrifft: «Bei meiner Arbeit habe ich immer öfter das Gefühl, emotional verbraucht zu sein». Die Aussage...	Arbeitssituation<br><br>SARSI25	Seco 2010 – 20100

	---<br>1 - Trifft überhaupt nicht zu<br>2 - Trifft eher nicht zu<br>3 - Trifft eher zu<br>4 - Trifft voll und ganz zu		
S4300<br><br>31 / {-}	LINK : IF 05200={1} OR 05201={1} OR 05202={1}<br>---<br>Geben Sie bitte wieder anhand der Skala an, inwieweit Ihr Beruf Folgendes einschliesst. Wie ist es mit:<br><br>a) Schmerzhaften oder ermüdenden Körperhaltungen<br>b) Tragen oder Bewegen von Personen<br>c) Tragen oder Bewegen schwerer Lasten<br>d) Stehen<br>e) Stets gleichen Hand- oder Armbewegungen<br><br>*INT : Bitte für jede Linie die entsprechende Antwort ankreuzen!*<br><br>*WEB : Bitte für jede Zeile die entsprechende Antwort auswählen!*<br>---<br>1 - Die ganze Zeit oder fast die ganze Zeit<br>2 - Ungefähr drei Viertel der Zeit<br>3 - Ungefähr die Hälfte der Zeit<br>4 - Ungefähr ein Viertel der Zeit<br>5 - Nie oder fast nie	Arbeitssituation<br><br><br>SARSI26a<br>SARSI26b<br>SARSI26c<br>SARSI26d<br>SARSI26e	SECO (M. Graf)<br>*Q11 EWCS (Switzerland) gekürzt*
S4400<br><br>32 / {-}	LINK : IF 05200={1} OR 05201={1} OR 05202={1}<br>---<br>Geben Sie bitte wieder anhand der Skala an, inwieweit Sie bei Ihrer Arbeit den folgenden Bedingungen ausgesetzt sind:<br><br>a) Vibrationen von Werkzeugen, Maschinen usw.<br>b) Starker Lärm, so dass man sich nur mit lauter Stimme mit anderen unterhalten kann<br>c) Hohe Temperaturen, so dass man schwitzt, auch wenn man nicht arbeitet<br>d) Niedrige Temperaturen am Arbeitsplatz drinnen oder draussen<br>e) Schädliche oder giftige Produkte oder Substanzen: Staub, Industrierauch, Mikroben, chemische Substanzen<br>f) Passivrauchen: Tabakrauch von anderen Personen<br><br>*INT : Bitte für jede Linie die entsprechende Antwort ankreuzen!*<br><br>*WEB : Bitte für jede Zeile die entsprechende Antwort auswählen!*<br>---<br>1 - Die ganze Zeit oder fast die ganze Zeit<br>2 - Ungefähr drei Viertel der Zeit<br>3 - Ungefähr die Hälfte der Zeit	Arbeitssituation<br>Immissionen<br><br><br>SARSI27a<br>SARSI27b<br>SARSI27c<br>SARSI27d<br>SARSI27e<br>SARSI27f	SECO (M. Graf)<br>Q10 EWCS (Switzerland)

	4 - Ungefähr ein Viertel der Zeit<br>5 - Nie oder fast nie		
S4500<br><br>33 / {-}	LINK : IF 05200={1} OR 05201={1} OR 05202={1}<br>---<br>Haben Sie in den letzten 12 Monaten am Arbeitsplatz Folgendes erfahren:<br><br>a) Benachteiligung aufgrund Ihres Alters<br>b) Benachteiligung aufgrund Ihres Geschlechts<br>c) Benachteiligung aufgrund Ihrer Nationalität, ethnischen Zugehörigkeit oder Hautfarbe<br>d) Benachteiligung aufgrund einer Behinderung<br>e) Verbale Gewalt<br>f) Drohungen und Erniedrigungen<br>g) Körperliche Gewalt<br>h) Einschüchterung, Belästigung, Mobbing<br>i) Sexuelle Belästigung<br>j) Ich habe keine dieser Benachteiligungen oder keine Gewalt erfahren<br><br>*INT : Bitte alles Zutreffende ankreuzen!*<br><br>*WEB : Bitte alles Zutreffende auswählen!*<br>---<br>1 - Trifft zu<br>0 - Trifft nicht zu	Arbeitssituation<br><br><br>SARSI28a<br>SARSI28b<br>SARSI28c<br>SARSI28d<br>SARSI28e<br>SARSI28f<br>SARSI28g<br>SARSI28h<br>SARSI28i<br>SARSI28j	SECO 2010 -11210 Frageitem 3 angepasst (Zusammenführung von EWCS Q65 B und C) und 4 (EWCS Q65 F)
S4700<br><br>34 / {-}	LINK : IF 05200={1} OR 05201={1} OR 05202={1}<br>---<br>Hier interessiert uns, wie Sie in letzter Zeit über Ihre Arbeit denken:<br><br>a) Meine Arbeit ist zwar nicht gerade ideal, aber schliesslich könnte sie noch schlimmer sein<br>b) Wenn sich bei meiner Arbeit nicht bald gewisse Sachen ändern, suche ich eine neue Stelle<br>c) Eine Kündigung würde mir noch mehr Nachteile bringen, darum bleibe ich trotz allem hier<br>d) Hoffentlich bleibt meine Arbeitssituation immer so gut, wie sie jetzt ist<br>e) Am besten schlucke ich den Ärger hinunter, wenn mich bei der Arbeit etwas stört<br>f) Nach arbeitsfreien Tagen freue ich mich richtig darauf, wieder an die Arbeit zu gehen<br>g) Als Arbeitnehmer/in kann ich wirklich nicht viel erwarten<br><br>*INT : Bitte für jede Linie die entsprechende Antwort ankreuzen!*<br><br>*WEB : Bitte für jede Zeile die entsprechende Antwort auswählen!*<br>---<br>1 - Fast immer<br>2 - Sehr oft<br>3 - Oft	Arbeitssituation<br><br><br>SARSI01<br>SARSI02<br>SARSI03<br>SARSI04<br>SARSI05<br>SARSI06<br>SARSI07	Anal. "Arbeits-zufriedenheit" 1995 (Semmer)<br>(Indizes SATRAV)

	4 - Hin und wieder<br>5 - Selten<br>6 - Sehr selten<br>7 - Fast nie		
S4800<br><br>35 / {-}	LINK : IF 05200={1} OR 05201={1} OR 05202={1}<br>---<br>Haben Sie Angst, Ihren heutigen Arbeitsplatz zu verlieren?<br>---<br>1 - Ja, sehr stark<br>2 - Ja, ziemlich<br>3 - Nein, eher nicht<br>4 - Nein, gar nicht	Arbeitssituation<br><br>SARSI09	BFS – SGB
S4900<br><br>36 / {-}	LINK : IF 05200={1} OR 05201={1} OR 05202={1}<br>---<br>Wenn Sie Ihren heutigen Arbeitsplatz / Lehrstelle verlieren würden: Wie leicht bzw. wie schwer wäre es für Sie, wieder eine gleichwertige Stelle zu finden?<br>---<br>1 - Sehr leicht<br>2 - Eher leicht<br>3 - Eher schwer<br>4 - Sehr schwer	Arbeitssituation<br><br>SARSI11	BFS – SGB
S5000<br><br>37 / {23}	PAPER: WIEDER AN ALLE (BEI NICHT ARBEIT)<br>---<br>Wie steht es mit Störungen bei Ihnen zu Hause? Gibt es häufig oder regelmässig Störungen, die Sie persönlich als lästig empfinden?<br><br>a) Lärm durch Strassenverkehr<br>b) Lärm von der Eisenbahn<br>c) Lärm von Flugzeugen<br>d) Lärm durch Industrie und Gewerbe<br>e) Lärm von Leuten oder Kindern, die nicht zum eigenen Haushalt gehören<br>f) Verkehrsabgase<br>g) Industrieabgase / Gestank<br>h) Störungen durch die Landwirtschaft<br>i) Elektromagnetische Felder von Hochspannungsleitungen<br>j) Strahlung von Mobilfunkantennen<br>k) Licht von Strassenbeleuchtungen, beleuchteten Nachbarhäusern oder Gärten, Sportplätzen, Leuchtreklamen usw.<br>l) Andere Störungen<br>m) Keine Störungen<br><br>*INT : Bitte alles Zutreffende ankreuzen!*<br><br>*WEB : Bitte alles Zutreffende auswählen!*	Umwelt<br><br>SUMWE01i<br>SUMWE01j<br>SUMWE01k<br>SUMWE01b<br>SUMWE01c<br>SUMWE01d<br>SUMWE01e<br>SUMWE01l<br>SUMWE01m<br>SUMWE01n<br>SUMWE01o<br>SUMWE01g<br>SUMWE01h	Anal. IGIP écrit 24<br>*ergänzt mit:*<br>*(SGB02: SUMWE01h-k)*<br>*(SGB07: SUMWE01l-n)*<br>*(SGB12: Pt a) angepasst)*<br>*(SGB17: SUMWE01o)*

	---<br>1 - Trifft zu<br>0 - Trifft nicht zu		
S7500<br><br><br>38 / {24}	Bitte geben Sie bei jeder Aussage an, inwieweit diese auf Sie persönlich zutrifft.<br><br>a) In schwierigen Situationen kann ich mich auf meine Fähigkeiten verlassen<br>b) Die meisten Probleme kann ich aus eigener Kraft gut meistern<br>c) Auch anstrengende und komplizierte Aufgaben kann ich in der Regel gut lösen<br>d) Ich versuche, mir etwas einfallen zu lassen, wie ich schwierige Situationen meistern kann<br>e) Egal, was mir passiert, ich glaube, ich habe meine Reaktionen unter Kontrolle<br>f) Ich glaube, ich kann mich weiterentwickeln, wenn ich mich mit schwierigen Situationen auseinandersetze<br>g) Ich suche aktiv nach Wegen, um die Verluste auszugleichen, die mir in meinem Leben widerfahren sind<br><br>*INT : Bitte für jede Linie die entsprechende Antwort ankreuzen!*<br><br>*WEB : Bitte für jede Zeile die entsprechende Antwort auswählen!*<br>---<br>1 - Trifft überhaupt nicht zu<br>2 - Trifft eher nicht zu<br>3 - Teils - teils<br>4 - Trifft eher zu<br>5 - Trifft voll und ganz zu	Psychische Gesundheit<br><br><br>SPSYG08a<br>SPSYG08b<br>SPSYG08c<br>SPSYG08d<br>SPSYG08e<br>SPSYG08f<br>SPSYG08g	Allgemeine Selbstwirksamkeit Kurzskala (ASKU) (GESIS DE)<br>Brief Resilient Coping Scale (BRCS);<br>(Sinclair & Wallston, 2004)
S5100<br><br><br>39 / {25}	Wie oft haben Sie sich im Verlauf der letzten 2 Wochen durch die folgenden Beschwerden beeinträchtigt gefühlt:<br><br>a) Wenig Interesse oder Freude an Ihren Tätigkeiten<br>b) Niedergeschlagenheit, Schwermut oder Hoffnungslosigkeit<br>c) Schwierigkeiten, ein- oder durchzuschlafen, oder vermehrter Schlaf<br>d) Müdigkeit oder Gefühl, keine Energie zu haben<br>e) Verminderter Appetit oder übermäßiges Bedürfnis zu essen<br>f) Schlechte Meinung von sich selbst; Gefühl, ein Versager zu sein oder die Familie enttäuscht zu haben<br>g) Schwierigkeiten, sich auf etwas zu konzentrieren, z. B. beim Zeitungslesen oder Fernsehen<br>h) Waren Ihre Bewegungen oder Ihre Sprache so verlangsamt, dass es auch anderen auffallen würde? Oder waren Sie im Gegenteil „zappelig“ oder ruhelos und hatten dadurch einen stärkeren Bewegungsdrang als sonst?<br>i) Gedanken, dass Sie lieber tot wären oder sich Leid zufügen möchten<br><br>*INT : Bitte für jede Linie die entsprechende Antwort ankreuzen!*<br><br>*WEB : Bitte für jede Zeile die entsprechende Antwort auswählen!*<br>---<br>1 - Überhaupt nicht<br>2 - An einzelnen Tagen	Psychische Gesundheit<br><br><br>SPSYG06a<br>SPSYG06b<br>SPSYG06c<br>SPSYG06d<br>SPSYG06e<br>SPSYG06f<br>SPSYG06g<br>SPSYG06h<br>SPSYG06i	PHQ - 9 French for Switzerland (écrit)

	3 - An mehr als der Hälfte der Tage<br>4 - Beinahe jeden Tag		
S7600<br><br>40 / {26}	Haben Sie jemals versucht, sich das Leben zu nehmen?<br>---<br>1 - Ja<br>2 - Nein **→Weiter zu Frage 41 S79.00**	Psychische Gesundheit<br><br>SSUIZ01	SMASH 2002 (Swiss multicenter adolescent survey on health)<br>F: SANTÉ ET STYLES DE VIE DES ADOLESCENTS ÂGÉS DE 16 À 20 ANS EN SUISSE (2002)
S7700<br><br>40.1 / {26.1}	LINK : IF S7600={1}<br>---<br>Haben Sie in den letzten 12 Monaten versucht, sich das Leben zu nehmen?<br>---<br>1 - Ja<br>2 - Nein	Psychische Gesundheit<br><br>SSUIZ02	SMASH 2002 (Swiss multicenter adolescent survey on health)<br>F: SANTÉ ET STYLES DE VIE DES ADOLESCENTS ÂGÉS DE 16 À 20 ANS EN SUISSE (2002)
S7800<br><br>40.2 / {26.2}	LINK : IF S7600={1}<br>---<br>Haben Sie nach Ihrem Suizidversuch mit jemandem darüber gesprochen?<br><br>a) Ja, mit jemandem aus meinem privaten Umfeld<br>b) Ja, mit einem Arzt/einer Ärztin oder einer Gesundheitsfachperson<br>c) Nein, mit niemandem<br><br>*INT : Bitte alles Zutreffende ankreuzen!*<br><br>*WEB : Bitte alles Zutreffende auswählen!*<br>---<br>1 - Trifft zu<br>0 - Trifft nicht zu	Psychische Gesundheit<br><br>SSUIZ03a<br>SSUIZ03b<br>SSUIZ03c	SMASH 2002 (Swiss multicenter adolescent survey on health)<br>F: SANTÉ ET STYLES DE VIE DES ADOLESCENTS ÂGÉS DE 16 À 20 ANS EN SUISSE (2002)
S7900<br><br>41 / {27}	WIEDER AN ALLE<br>---<br>Bei den folgenden Fragen geht es ausschließlich um das, was Sie in Ihrer Freizeit oder privat im Internet tun - also nicht um berufliche oder schulische Aktivitäten im Internet.<br><br>Nutzen Sie das Internet in Ihrer Freizeit oder zu privaten Zwecken? Mit dem Internet ist das Onlinegehen mit einem Computer, einem Smartphone, einem Tablet oder iPad gemeint, um zu surfen, zu mailen, zu chatten oder zu gamen.<br>---<br>1 - Ja<br>2 - Nein **→Weiter zu Frage 42 S82.00**	Internetnutzung<br><br>SINNU01	COROLAR Split A Welle 9 2015 (angepasst)

S8000<br><br>41.1 / {27.1}	LINK : IF S7900={1}<br>---<br>Wie oft benützen Sie zu privaten Zwecken das Internet?<br>---<br>1 - Mindestens 1 Mal am Tag<br>2 - Mindestens 1 Mal in der Woche (aber nicht täglich)<br>3 - Mindestens 1 Mal im Monat (aber nicht wöchentlich)<br>4 - Weniger als 1 Mal im Monat	Internetnutzung<br><br>SINNU02	STATCAN, Enquête canadienne sur l'utilisation d'internet (ECUI), 2012 (angepasst)
S8100<br><br>41.2 / {27.2}	LINK : IF S7900={1}<br>---<br>Bitte geben Sie an, wie häufig Sie jeweils die geschilderten Situationen in den letzten 2 Wochen selbst erlebt haben.<br><br>a) Mir fällt es schwer, die Internetsitzung zu beenden, wenn ich online bin<br>b) Nahestehende Personen (z.B. Partner, Freunde, Familie) sagen mir, ich solle das Internet weniger häufig nutzen<br>c) Ich nutze lieber das Internet, anstatt mit anderen (z.B. Partner, Freunde, Familie) Zeit zu verbringen<br>d) Ich bin wegen meiner Internetnutzung unausgeschlafen<br>e) Ich sehne mich nach der nächsten Internetsitzung<br>f) Ich habe erfolglos versucht, weniger Zeit im Internet zu verbringen<br>g) Ich vernachlässige gewisse Sachen (z.B. Studium, Arbeit, Aktivitäten mit Freunden oder mit der Familie), weil ich lieber online bin<br>h) Ich nutze das Internet, wenn ich mich nicht gut fühle (traurig, deprimiert)<br>i) Ich fühle mich ruhelos, frustriert oder gereizt, wenn ich das Internet nicht nutzen kann<br><br>*INT : Bitte für jede Linie die entsprechende Antwort ankreuzen!*<br><br>*WEB : Bitte für jede Zeile die entsprechende Antwort auswählen!*<br>---<br>1 - Nie<br>2 - Selten<br>3 - Manchmal<br>4 - Häufig<br>5 - Sehr häufig	Internetnutzung<br><br>SINNU03a<br>SINNU03b<br>SINNU03c<br>SINNU03d<br>SINNU03e<br>SINNU03f<br>SINNU03g<br>SINNU03h<br>SINNU03i	Compulsive Internet Use Scale (CIUS-9)
S8200<br><br>42 / {28}	WIEDER AN ALLE<br>---<br>Wieviel Zeit pro Tag verbringen Sie in Ihrer Freizeit normalerweise mit folgenden Aktivitäten?<br><br>a) Fernsehen oder Videos schauen<br>b) Computer- oder Videospiele spielen<br>c) Anderweitige Benützung eines Computers, Smartphones, Tablets aber nicht zum Fernsehen oder Video schauen, noch zum Computer- oder Videospielen, noch zum Telefonieren<br><br>*INT : Bitte für jede Linie die entsprechende Antwort ankreuzen!*	Elektronische Mediennutzung<br><br>SEMNU01a<br>SEMNU01b<br>SEMNU01c	Enquête sur le tabagisme chez les jeunes (ETJ) 2008-2009 Canada (angepasst)

	WEB : Bitte für jede Zeile die entsprechende Antwort auswählen!<br>---<br>1 - Nie / nicht täglich<br>2 - Weniger als 1 Stunde am Tag<br>3 - 1 bis 2 Stunden am Tag<br>4 - Über 2 Stunden bis unter 5 Stunden am Tag<br>5 - 5 Stunden am Tag oder mehr		
S5300<br><br><br>43 / {29}<br><br><br>*PAPER*	Welche Spiele haben Sie im Laufe Ihres Lebens schon gespielt ? Geben Sie für jedes dieser Spiele an, wie häufig Sie dieses in den letzten 12 Monaten gespielt haben :<br><br>Glücksspiele in Schweizer Casinos:<br>a) Tischspiele (z.B. Roulette, Black-Jack)<br>b) Automaten, Slot Maschinen<br><br>Glücksspiele bei Schweizer Lotteriegesellschaften (Swisslos, Loterie Romande):<br>a) Lotterien (z.B. Swiss Lotto Euro Millions, Rubbellose, Loterie Electronique)<br>b) Sportwetten<br><br>Andere Glücksspiele in der Schweiz:<br>a) z.B. Tombola, privat, Spiel in “Hinterzimmern”<br><br>Ausländische Glücksspiele:<br>a) Bei internationalen Onlineanbietern (z.B. bwin, Interwetten, Betfair, Partypoker)<br>b) Spielhallen und Casinos im Ausland, ausländische Lotterien<br><br>Im Laufe des Lebens:<br>1=Ja<br>2=Nein<br><br>*INT : Bitte für jede Linie das Zutreffende ankreuzen!*<br>---<br>1 - Nie<br>2 - Weniger als 1 Mal pro Monat<br>3 - 1 bis 3 Mal pro Monat<br>4 - 1 Mal pro Woche<br>5 - Mehr als 1 Mal pro Woche	Glücksspiel<br><br><br>SGLSP17a<br>SGLSP18a<br>SGLSP19a<br>SGLSP20a<br>SGLSP21a<br>SGLSP22a<br>SGLSP23a<br>SGLSP17b<br>SGLSP18b<br>SGLSP19b<br>SGLSP20b<br>SGLSP21b<br>SGLSP22b<br>SGLSP23b	ISGF ZH; ESBK<br>*SGB17: Antwortmöglichkeiten “Ja - Nein” sind in der Papierversion in der umgekehrten Reihenfolge*
S5300_WEB_1<br><br><br>43 / {29}	Welche Spiele haben Sie im Laufe Ihres Lebens schon gespielt?<br><br>Glücksspiele in Schweizer Casinos:<br>a) Tischspiele (z.B. Roulette, Black-Jack)<br>b) Automaten, Slot Maschinen	Glücksspiel<br><br><br>SGLSP17a<br>SGLSP18a<br>SGLSP19a	ISGF ZH; ESBK

ESURVEY	Glücksspiele bei Schweizer Lotteriegesellschaften (Swisslos, Loterie Romande):<br>a) Lotterien (z.B. Swiss Lotto Euro Millions, Rubbellose, Loterie Electronique)<br>b) Sportwetten<br><br>Andere Glücksspiele in der Schweiz:<br>a) z.B. Tombola, privat, Spiel in "Hinterzimmern"<br><br>Ausländische Glücksspiele:<br>a) Bei internationalen Onlineanbietern (z.B. bwin, Interwetten, Betfair, Partypoker)<br>b) Spielhallen und Casinos im Ausland, ausländische Lotterien<br><br>*INT : Bitte für jede Linie das Zutreffende ankreuzen!*<br><br>*WEB : Bitte für jede Zeile das Zutreffende auswählen!*<br>---<br>1 - Ja<br>2 - Nein →**Wenn nie gespielt, weiter zu Frage 46 S58.00**	SGLSP20a<br>SGLSP21a<br>SGLSP22a<br>SGLSP23a	
S5300_WEB_2<br><br>43.1 / {29}<br><br>*ESURVEY*	LINK : IF S5300_WEB_1_a={1} or S5300_WEB_1_b={1} or S5300_WEB_1_c={1} or S5300_WEB_1_d={1} or S5300_WEB_1_e={1} or S5300_WEB_1_f={1} or S5300_WEB_1_g={1}<br>---<br>Wie häufig haben Sie diese Spiele in den letzten 12 Monaten gespielt?<br><br>Glücksspiele in Schweizer Casinos:<br>a) Tischspiele (z.B. Roulette, Black-Jack)<br>b) Automaten, Slot Maschinen<br><br>Glücksspiele bei Schweizer Lotteriegesellschaften (Swisslos, Loterie Romande):<br>a) Lotterien (z.B. Swiss Lotto Euro Millions, Rubbellose, Loterie Electronique)<br>b) Sportwetten<br><br>Andere Glücksspiele in der Schweiz:<br>a) z.B. Tombola, privat, Spiel in "Hinterzimmern"<br><br>Ausländische Glücksspiele:<br>a) Bei internationalen Onlineanbietern (z.B. bwin, Interwetten, Betfair, Partypoker)<br>b) Spielhallen und Casinos im Ausland, ausländische Lotterien<br><br>*INT : Bitte für jede Linie das Zutreffende ankreuzen!*<br><br>*WEB : Bitte für jede Zeile das Zutreffende auswählen!*<br>---<br>1 - Nie → **S84.00**	Glücksspiel<br><br>SGLSP17b<br>SGLSP18b<br>SGLSP19b<br>SGLSP20b<br>SGLSP21b<br>SGLSP22b<br>SGLSP23b	ISGF ZH; ESBK

	2 - Weniger als 1 Mal pro Monat<br>3 - 1 bis 3 Mal pro Monat<br>4 - 1 Mal pro Woche<br>5 - Mehr als 1 Mal pro Woche		
S8300<br><br>44 / {30}<br><br>*PAPER*	Personen, die nie im Leben Glücksspiele gespielt haben, weiter mit Frage S5800/46<br>---<br>Welchen Geldbetrag haben Sie in den letzten 12 Monaten durchschnittlich pro Monat für Glücksspiele ausgegeben?<br><br>*INT : Bitte nur ein einziges Kästchen ankreuzen!*<br>---<br>1 - Weniger als CHF 10.-<br>2 - Zwischen CHF 10.- und 99.-<br>3 - Zwischen CHF 100.- und 299.-<br>4 - Zwischen CHF 300.- und 999.-<br>5 - Zwischen CHF 1'000.- und 2'499.-<br>6 - Zwischen CHF 2'500.- und 9'999.-<br>7 - CHF 10'000.- oder mehr<br>8 - Ich habe in den letzten 12 Monaten nicht gespielt	Glücksspiel<br><br>SGLSP24	ESBK (F. Soum)<br>*ESS17: énoncé de la question précisé analog SGB07: SGLSP05*
S8300_WEB<br><br>44 / {30}<br><br>*ESURVEY*	LINK : IF S5300_WEB_2_a={2,3,4,5} or S5300_WEB_2_b={2,3,4,5} or S5300_WEB_2_c={2,3,4,5} or S5300_WEB_2_d={2,3,4,5} or S5300_WEB_2_e={2,3,4,5} or S5300_WEB_2_f={2,3,4,5} or S5300_WEB_2_g={2,3,4,5}<br><br>Personen, die in den letzten 12 Monaten keine Glücksspiele gespielt haben, weiter mit Frage S8400/45<br>---<br>Welchen Geldbetrag haben Sie in den letzten 12 Monaten durchschnittlich pro Monat für Glücksspiele ausgegeben?<br><br>*WEB : Bitte nur eine einzige Antwort auswählen!*<br>---<br>1 - Weniger als CHF 10.-<br>2 - Zwischen CHF 10.- und 99.-<br>3 - Zwischen CHF 100.- und 299.-<br>4 - Zwischen CHF 300.- und 999.-<br>5 - Zwischen CHF 1'000.- und 2'499.-<br>6 - Zwischen CHF 2'500.- und 9'999.-<br>7 - CHF 10'000.- oder mehr	Glücksspiel<br><br>SGLSP24	ESBK (F. Soum)<br>*ESS17: énoncé de la question précisé analog SGB07: SGLSP05*
S8400<br><br>45 / {31}	LINK : IF S5300_WEB_1_a={1} or S5300_WEB_1_b={1} or S5300_WEB_1_c={1} or S5300_WEB_1_d={1} or S5300_WEB_1_e={1} or S5300_WEB_1_f={1} or S5300_WEB_1_g={1}<br>---<br>Bitte beantworten Sie die folgenden Fragen zu Glücksspielen um Geld :<br><br>a) Haben Sie jemals versucht, das Spielen aufzugeben, einzuschränken oder zu kontrollieren?<br>b) Haben Sie jemals Familienmitglieder, Freunde oder andere darüber belogen, wie häufig Sie spielen oder wie viel Geld Sie beim Spielen verloren haben?	Glücksspiel<br><br>SGLSP25a<br>SGLSP25b<br>SGLSP25c<br>SGLSP25d<br>SGLSP25e	Items a-c aus NODS-CliP (ISGF ZH; ESBK)<br><br>*SGBS17: Fragen ergänzt und Antworten zeitlich angepasst*

	c) Haben Sie jemals eine Phase von mindestens zwei Wochen erlebt, wo Sie viel Zeit verbracht haben mit dem Nacherleben von früheren Spielerfahrungen oder mit dem Planen von den nächsten Spielgelegenheiten?<br>d) Haben Sie jemals das Bedürfnis verspürt, mit einem immer höheren Geldeinsatz zu spielen?<br>e) Hat Ihnen Ihr Spielverhalten jemals ernste oder wiederholte Probleme bei Ihrer Arbeit oder in Ihrer Beziehung zu Familienmitgliedern und Freunden verursacht?<br><br>*INT : Bitte für jede Linie die entsprechende Antwort ankreuzen!*<br><br>*WEB : Bitte für jede Zeile die entsprechende Antwort auswählen!*<br>---<br>1 - Nie<br>2 - Ja, vor mehr als 12 Monaten<br>3 - Ja, in den letzten 12 Monaten		
S5800<br><br>46 / {32}<br><br>*PAPER*	WIEDER AN ALLE<br>---<br>Leben in Ihrem Haushalt Kinder unter 15 Jahren?<br>---<br>1 - Ja<br>2 - Nein **→Falls in Ihrem Haushalt keine Kinder unter 15 Jahre leben, sind Sie am Ende des Fragebogens angelangt. Wir danken Ihnen herzlich für Ihre wertvollen Auskünfte !**	Kinder<br><br>SKID	Question filtre introductive pour e-survey<br>*SGB17: Filterfrage in Papierfragebogen verwendet.*<br>*Für Onlinefragebogen sind die Fragen S5810 bis S8700 gemäss CATI gefiltert*
S5810<br><br>47 / {33}<br><br>*PAPER*	Welches ist das Geschlecht und das Geburtsdatum dieser Kinder?<br><br>a) Jüngstes Kind Männlich=1 Weiblich= 2 _ _ Tag _ _ Monat _ _ _ _ Jahr<br>b) Zweitjüngstes Kind Männlich=1 Weiblich=2 _ _ Tag _ _ Monat _ _ _ _ Jahr<br>c) Drittjüngstes Kind Männlich=1 Weiblich=2 _ _ Tag _ _ Monat _ _ _ _ Jahr<br><br>*INT : Nur für Kinder unter 15 Jahren*	Kinder<br><br>SKIDNB<br>SKISEXa<br>SKISEXb<br>SKISEXc<br>SKIALTERa<br>SKIALTERb<br>SKIALTERc<br>[SKITT01abc]<br>[SKIMM01abc]<br>[SKIJJ01abc]	IGIP écrit 37+38<br>*SGB17: e-survey mit Info der Register (CATI basis) ausgefüllt*<br>*(SKIDNB=Anzahl Kinder (<15 Jahre))*
S5810_WEB<br><br>47 / {33}<br><br>*ESURVEY*	LINK : if NBKIDS>0<br>---<br>Es folgen nun noch 4 Fragen zur Gesundheit der Kinder in Ihrem Haushalt.<br>Beim Interview am Telefon haben Sie uns gesagt, dass in Ihrem Haushalt die folgenden Kinder unter 15 Jahren leben:<br><br>a) {Vorname} {Alter}<br>b) {Vorname} {Alter}<br>c) {Vorname} {Alter}	Kinder<br><br>SKIDNB<br>SKISEXa<br>SKISEXb<br>SKISEXc<br>SKIALTERa<br>SKIALTERb	IGIP écrit 37+38<br>*SGB17: e-survey mit Info der Register (CATI basis) ausgefüllt*<br>*(SKIDNB=Anzahl Kinder (<15 Jahre))*

	WEB : Bitte mit dem jüngsten Kind anfangen!	SKIALTERc<br>[SKITT01abc]<br>[SKIMM01abc]<br>[SKIJJ01abc]	
S8500<br><br>48 / {34}<br><br>*PAPER*	Wie würden Sie den Gesundheitszustand dieser Kinder im Allgemeinen beschreiben?<br><br>a) Jüngstes Kind<br>b) Zweitjüngstes Kind<br>c) Drittjüngstes Kind<br><br>*INT : Nur für Kinder unter 15 Jahren.*<br>---<br>1 - Sehr gut<br>2 - Gut<br>3 - Mittelmässig<br>4 - Schlecht<br>5 - Sehr schlecht	Kinder<br><br><br>SKSUBG01a<br>SKSUBG01b<br>SKSUBG01c	Eurostat MEHM1 - EHIS 2014
S8500_WEB<br><br>48 / {34}<br><br>*ESURVEY*	LINK : IF NBKIDS>0<br>---<br>Wie würden Sie den Gesundheitszustand dieser Kinder im Allgemeinen beschreiben?<br><br>a) {Vorname} {Alter}<br>b) {Vorname} {Alter}<br>c) {Vorname} {Alter}<br>---<br>1 - Sehr gut<br>2 - Gut<br>3 - Mittelmässig<br>4 - Schlecht<br>5 - Sehr schlecht	Kinder<br><br><br>SKSUBG01a<br>SKSUBG01b<br>SKSUBG01c	Eurostat MEHM1 - EHIS 2014
S8600<br><br>49 / {35}<br><br>*PAPER*	Wie gut trifft folgende Beschreibung auf diese Kinder zu?<br><br>a) Jüngstes Kind<br>b) Zweitjüngstes Kind<br>c) Drittjüngstes Kind<br><br>1) Ist unruhig, überaktiv, kann nicht lange stillsitzen<br>2) Klagt häufig über Kopfschmerzen, Bauchschmerzen oder Übelkeit<br>3) Hat oft Wutanfälle, ist aufbrausend<br><br>*INT : Nur für Kinder unter 15 Jahren. Bitte pro Kind in jeder Linie die entsprechende Antwort ankreuzen!*	Kinder<br><br><br>SKPRO01a<br>SKPRO01b<br>SKPRO01c<br>SKPRO02a<br>SKPRO02b<br>SKPRO02c<br>SKPRO03a<br>SKPRO03b<br>SKPRO03c	Items aus Strengths and Difficulties Questionnaire (SDQ)

	---<br>1 - Nicht zutreffend<br>2 - Teilweise zutreffend<br>3 - Eindeutig zutreffend		
S8600_WEB<br><br>49 / {35}<br><br>*ESURVEY*	LINK : IF NBKIDS>0<br>---<br>Wie gut trifft folgende Beschreibung auf diese Kinder zu?<br><br>a) {Vorname} {Alter}<br>b) {Vorname} {Alter}<br>c) {Vorname} {Alter}<br><br>1) Ist unruhig, überaktiv, kann nicht lange stillsitzen<br>2) Klagt häufig über Kopfschmerzen, Bauchschmerzen oder Übelkeit<br>3) Hat oft Wutanfälle, ist aufbrausend<br><br>*WEB : Bitte pro Kind in jeder Zeile die entsprechende Antwort auswählen!*<br>---<br>1 - Nicht zutreffend<br>2 - Teilweise zutreffend<br>3 - Eindeutig zutreffend	Kinder<br><br>SKPRO01a<br>SKPRO01b<br>SKPRO01c<br>SKPRO02a<br>SKPRO02b<br>SKPRO02c<br>SKPRO03a<br>SKPRO03b<br>SKPRO03c	Items aus Strengths and Difficulties Questionnaire (SDQ)
S6100<br><br>50 / {36}<br><br>*PAPER*	Sind diese Kinder gestillt worden? Und wenn ja, wie lange wurden sie ausschliesslich gestillt?<br><br>a) Jüngstes Kind<br>b) Zweitjüngstes Kind<br>c) Drittjüngstes Kind<br><br>*INT : Nur für Kinder unter 15 Jahren*<br>---<br>1 - Nicht gestillt<br>2 - 0 bis 4 Monate<br>3 - Mehr als 4 bis 6 Monate<br>4 - Mehr als 6 bis 12 Monate<br>5 - Mehr als 12 Monate<br>6 - Gestillt, weiss aber nicht mehr wie lange	Kinder<br>Stillen<br><br>SKSTI05a<br>SKSTI05b<br>SKSTI05c	Gemäss Empfehlungen von EURO-DIET<br>*SGB17: SKSTI02a-c und SKSTI03a-c zusammen (+ bei SKSTI03a-c Werte 1 und 2 zusammen)*
S6100_WEB<br><br>50 / {36}<br><br>*ESURVEY*	LINK : IF NBKIDS>0<br>---<br>Sind diese Kinder gestillt worden? Und wenn ja, wie lange wurden sie ausschliesslich gestillt?<br><br>a) {Vorname} {Alter}<br>b) {Vorname} {Alter}<br>c) {Vorname} {Alter}	Kinder<br>Stillen<br><br>SKSTI05a<br>SKSTI05b<br>SKSTI05c	Gemäss Empfehlungen von EURO-DIET<br>*SGB17: SKSTI02a-c und SKSTI03a-c zusammen (+ bei SKSTI03a-c Werte 1 und 2 zusammen)*

	---<br>1 - Nicht gestillt<br>2 - 0 bis 4 Monate<br>3 - Mehr als 4 bis 6 Monate<br>4 - Mehr als 6 bis 12 Monate<br>5 - Mehr als 12 Monate<br>6 - Gestillt, weiss aber nicht mehr wie lange		
S6300<br><br><br>51 / {37}<br><br><br>*PAPER*	Haben diese Kinder irgendwelche Behinderungen? Falls ja, wie stark beeinflussen diese die Schulungsfähigkeit oder sonstigen Tätigkeiten, die ein Kind in diesem Alter unternimmt?<br><br>a) Jüngstes Kind<br>b) Zweitjüngstes Kind<br>c) Drittjüngstes Kind<br><br>*INT : Nur für Kinder unter 15 Jahren*<br>---<br>0 - Keine Behinderung → **END**<br>1 - Behinderung hat einen kleinen oder gar keinen Einfluss<br>2 - Behinderung hat einen gewissen Einfluss<br>3 - Behinderung hat einen starken Einfluss	Kinder<br>Behinderung<br><br><br>SKBEH04a<br>SKBEH04b<br>SKBEH04c	IGIP schr. 41 + 41.1<br>*SGB12: Antwortskalen 1 und 3 wurden gegeneinander ausgetauscht!*
S6300_WEB<br><br><br>51 / {37}<br><br><br>*ESURVEY*	LINK : IF NBKIDS>0<br>---<br>Haben diese Kinder irgendwelche Behinderungen? Falls ja, wie stark beeinflussen diese die Schulungsfähigkeit oder sonstigen Tätigkeiten, die ein Kind in diesem Alter unternimmt?<br><br>a) {Vorname} {Alter}<br>b) {Vorname} {Alter}<br>c) {Vorname} {Alter}<br><br>*INT : Nur für Kinder unter 15 Jahren*<br>---<br>0 - Keine Behinderung → **END**<br>1 - Behinderung hat einen kleinen oder gar keinen Einfluss<br>2 - Behinderung hat einen gewissen Einfluss<br>3 - Behinderung hat einen starken Einfluss	Kinder<br>Behinderung<br><br><br>SKBEH04a<br>SKBEH04b<br>SKBEH04c	IGIP schr. 41 + 41.1<br>*SGB12: Antwortskalen 1 und 3 wurden gegeneinander ausgetauscht!*
S8700<br><br><br>51.1 / {37.1}<br><br><br>*PAPER*	Falls eine Behinderung vorliegt, was ist die Hauptursache dafür?<br><br>a) Jüngstes Kind<br>b) Zweitjüngstes Kind<br>c) Drittjüngstes Kind<br><br>*INT : Bitte nur ein einziges Kästchen ankreuzen!*	Kinder<br>Behinderung<br><br><br>SKBEH05a<br>SKBEH05b<br>SKBEH05c	BFS - HAND

	---<br>1 - Sensorisch<br>2 - Körperlich<br>3 - Geistig<br>4 - Verhaltensstörungen		
S8700_WEB<br><br>51.1 / {37.1}<br><br>*ESURVEY*	LINK : IF NBKIDS>0<br>---<br>Was ist die Hauptursache für die Behinderung?<br><br>a) {Vorname} {Alter}<br>b) {Vorname} {Alter}<br>c) {Vorname} {Alter}<br><br>*WEB : Bitte nur eine einzige Antwort auswählen!*<br>---<br>1 - Sensorisch<br>2 - Körperlich<br>3 - Geistig<br>4 - Verhaltensstörungen	Kinder<br>Behinderung<br><br>SKBEH05a<br>SKBEH05b<br>SKBEH05c	BFS - HAND
THANKS	Sie sind am Ende des Fragebogens angelangt.<br>Wir danken Ihnen herzlich für Ihre wertvollen Auskünfte !		
